# Expanded Alcohol Screening and Brief Intervention to Address Premature Mortality

**DOI:** 10.1001/jamahealthforum.2026.2348

**Published:** 2026-07-31

**Authors:** Julia M. Lemp, Carolin Kilian, Xinyi Kou, Charlotte Buckley, Laura Llamosas-Falcón, Yachen Zhu, Jürgen Rehm, Nina Mulia, Robin Purshouse, Charlotte Probst

**Affiliations:** 1 Institute for Mental Health Policy Research, Centre for Addiction and Mental Health, Toronto, Canada; 2 Heidelberg Institute of Global Health, Heidelberg University Hospital, Heidelberg, Germany; 3 National Institute of Public Health, University of Southern Denmark, Copenhagen, Denmark; 4 Danish Institute for Advanced Study, University of Southern Denmark, Odense, Denmark; 5 Center for Interdisciplinary Addiction Research, Department of Psychiatry and Psychotherapy, University Medical Center Hamburg-Eppendorf, Hamburg, Germany; 6 School of Electrical and Electronic Engineering, University of Sheffield, Sheffield, UK; 7 Department of Psychology, Institute of Population Health, University of Liverpool, Liverpool, UK; 8 Institute of Medical Science, Faculty of Medicine, University of Toronto, Toronto, Ontario, Canada; 9 Alcohol Research Group, Public Health Institute, Emeryville, California; 10 Campbell Family Mental Health Research Institute, Centre for Addiction and Mental Health, Toronto, Ontario, Canada; 11 Department of Psychiatry, University of Toronto, Toronto, Ontario, Canada; 12 Dalla Lana School of Public Health, University of Toronto, Toronto, Ontario, Canada; 13 Program on Substance Abuse & World Health Organization Collaborating Centre, Public Health Agency of Catalonia, Barcelona, Spain

## Abstract

**Importance:**

Alcohol use is a leading cause of premature mortality in the US. Despite public health recommendations and insurance coverage mandates, alcohol screening and brief intervention (ASBI) in primary care remains underused.

**Objective:**

To estimate the population-level impact of expanded ASBI delivery on alcohol use and potential years of life lost (YLL) from major alcohol-related causes of death in US adults through 2030, by sex, race and ethnicity, and educational attainment as a proxy for socioeconomic status.

**Design, Setting, and Participants:**

This was a decision analytical study that used a dynamic individual-level microsimulation model of nationally representative of US adults (age ≥18 years) from 2000 to 2030 and considered sociodemographic variables, alcohol use, and mortality risks.

**Interventions:**

Four expansion scenarios increasing the annual number of eligible individuals receiving ASBI from 2025 to 2030: scenario 1, expanding alcohol screening (AS) only; scenario 2, expanding brief intervention (BI) by 4 million; scenario 3, expanding BI by 8 million; and scenario 4, universal ASBI delivery. Scenarios were compared with a no-expansion counterfactual assuming historical trends.

**Main Outcomes and Measures:**

Prevalence of hazardous alcohol use (>20 or >40 g of alcohol per day for women and men) and YLL per 100 000 before age 75 years from 5 key alcohol-related causes of death (alcohol use disorder, liver disease and cirrhosis, motor vehicle injuries, other unintentional injuries, and suicide) in 2030.

**Results:**

The analysis of the microsimulation model found that scenario 3 (additional 8 million BI) and scenario 4 (universal ASBI) had the strongest impact on alcohol use and mortality, and were projected to reduce annual alcohol-related YLL per 100 000 by −51.3 (95% credible interval [CrI], −73.2 to −32.8) and −68.9 (CrI, −102.2 to −44.8) among men, respectively, and −34.1 (CrI, −54.5 to −16.1) and −52.0 (CrI, −75.0 to −30.2) among women, respectively, by 2030. In scenario 4, the gap in YLL from causes associated with alcohol use between low and high education narrowed by −3.5% (−64.5 YLL per 100 000; 95% CrI, −106.3 to −35.9) among men and −4.9% (−31.4 YLL per 100 000; 95% CrI, −56.2 to −12.4) among women.

**Conclusions and Relevance:**

In decision analytical model expanding ASBI delivery in primary care was projected to reduce premature mortality and may represent an important strategy for improving population health. Substantial expansion had measurable but modest potential to narrow socioeconomic inequalities in premature mortality from causes of death associated with alcohol use.

## Key Points

**Question:**

What is the potential impact of expanding alcohol screening and brief interventions in primary care on premature mortality from major alcohol-related causes of death in US adults?

**Findings:**

In this decision analytical model and microsimulation study, expanding delivery of alcohol brief interventions to an additional 8 million interventions per year was projected to reduce annual alcohol-related potential years of life lost per 100 000 adults by −51.3 (95% credible interval [CrI], −73.2 to −32.8) in men and −34.1 (CrI, −54.5 to −16.1) in women by 2030 compared with the status quo.

**Meaning:**

These findings suggest that substantially expanding alcohol screening and brief interventions in primary care may reduce premature mortality and help narrow the socioeconomic gap in alcohol-related mortality in the US.

## Teaser

This decision analytical model estimates the population-level impact of expanded alcohol screening and brief intervention delivery on alcohol use and potential years of life lost from major alcohol-related causes of death among US adults.

## Introduction

Screening and brief intervention for alcohol use is a core component of preventive care. The US Preventive Services Task Force recommends that all adults in primary care settings be screened for alcohol use and that those engaging in hazardous or risky drinking receive brief behavioral counseling.^1^ Under the Affordable Care Act, commercial health insurers are required to cover these services at no additional cost to patients, and most Medicare & Medicaid plans cover at least annual provision of alcohol screening and brief intervention (ASBI).^2,3^

Despite these mandates, implementation of ASBI remains limited, with access differing across population groups.^4,5^ Routine health care visits constitute a major entry point to ASBI, yet 15% of US adults had no health care visit in the past 12 months.^6^ Even among those who did, survey data suggest that 4 in 5 adults who attended a “health checkup” were asked about their alcohol use, while only 1 in 5 who engaged in risky drinking received advice to reduce drinking.^4^ This is especially concerning given that alcohol use is among the most important risk factors for premature mortality in the US. In 2021, alcohol was estimated to account for more than 111 500 deaths across all ages, the highest number recorded since 1990.^7^ In addition, deaths from causes associated with alcohol use are concentrated among population groups with lower educational attainment, making alcohol-attributable mortality a major contributor to the widening socioeconomic gap in life expectancy in the US.^8^

Low ASBI rates among individuals with hazardous alcohol use may represent a missed opportunity to mitigate the growing alcohol-related burden. While brief interventions (BIs) are effective at reducing alcohol use at the individual level,^9^ their potential to counteract national trends in premature mortality and variation across sociodemographic groups in the US has received little attention. Our study used an advanced microsimulation to model how expanding ASBI at different stages of the care cascade may affect alcohol use and potential years of life lost (YLL) from key alcohol-related causes of death through 2030 in the adult US population, by sex, race and ethnicity, and educational attainment.

## Methods

This study is part of the Simulation of Alcohol Control Policies for Health Equity (SIMAH) project,^10^ which has been reviewed and approved by the Centre for Addiction and Mental Health Research Ethics Board (#115/2020). The underlying computer model is documented using the Overview, Design Concepts and Details (ODD) protocol available in the eMethods 1 in Supplement 1, a recognized best practice for reporting simulation models.^11^ We followed the Consolidated Health Economic Evaluation Reporting Standards (CHEERS) reporting guideline.

### Model Overview

A dynamic, individual-level microsimulation model was developed, calibrated, and validated to simulate alcohol use and deaths due to alcohol-related causes over 30 years from 2000 through 2030, progressing in 1-year time steps. Individuals in the microsimulation constituted a synthetic population representative of the actual US population aged 18 to 79 years. Individuals were characterized by educational attainment (a proxy for socioeconomic status, denoted “education”), race and ethnicity, age, and sex. Education was categorized as high school degree or less (≤ high school), some college, and college degree or more (≥ college) and synthetic individuals could progress in their education until the age of 34 years. Race and ethnicity were categorized as non-Hispanic White (hereafter, White), non-Hispanic Black (Black), Hispanic, and other (combining American Indian or Alaska Native, Asian, and Native Hawaiian or Other Pacific Islander due to small sample sizes; additional details in eMethods, sections 1.3.3, in Supplement 1). Alcohol use was modeled based on each individual’s characteristics, where individuals had the opportunity to change their alcohol consumption level each year (eMethods, sections 1.1.2 and 1.3.5, in Supplement 1). Four expansion scenarios for ASBI delivery were modeled and compared to a reference scenario. The reference scenario assumed continuation of historical trends in ASBI rates (ie, no expansion beyond observed trends; eMethods, sections 1.3.5, in Supplement 1). Each expansion scenario increased the number of individuals receiving ASBI starting in the year 2025. For each comparison, annual subgroup-specific mean effect estimates and 95% credible intervals (CrIs) were quantified to 2030, reflecting the impact of expansion over a 6-year period.

### Outcomes

Primary outcomes were (1) prevalence of hazardous alcohol use and (2) combined YLL per 100 000 adults from alcohol use disorder (AUD, including alcohol poisonings), liver disease and cirrhosis (including hepatitis C−related liver cirrhosis), motor vehicle injuries, other unintentional injuries, and suicide (eMethods, section 1.1.2, in Supplement 1 for codes from the *International Statistical Classification of Diseases and Related Health Problems, Tenth Revision*). Each of these causes has an alcohol-attributable fraction of 15% or more in the US.^8,12^ YLL was calculated as the difference between a benchmark age of 75 years (following the general practice of most federal and state agencies’ YLL calculations^13,14,15^) and the age at death and standardized per 100 000 adults, and were assessed in absolute and relative terms compared to the reference scenario. Hazardous alcohol use was defined as average consumption exceeding 20 g of pure alcohol per day (GPD) for women or 40 GPD for men, consistent with the World Health Organization’s classification of drinking levels based on sex-specific daily alcohol consumption and associated alcohol-related harm,^16^ thereby defining the population eligible for BI in the model. A secondary outcome was heavy episodic drinking prevalence (HED; defined as 60 g or more of pure alcohol on at least 1 occasion in the past 30 days), which is updated annually and modifies the cause-specific risk functions for injuries (eMethods, section 1.3.5, in Supplement 1).

### Data Input and Model Validation

#### Data Input

Microsimulation parameters were informed by US data sources and existing studies (eMethods, section 1.3, in Supplement 1). In brief, population estimates were based on decennial US Census Bureau data^17^ and the annual American Community Survey (ACS)^18^; transitions between levels of education (by race and ethnicity, age, and sex) were informed by data from the Panel Study of Income Dynamics.^19^ Individual-level data to inform alcohol exposure and changes in alcohol use over time were retrieved from the Behavioral Risk Factor Surveillance System (BRFSS)^20^ and adjusted for underreporting.^21^ Mortality was based on individual death records from the US National Vital Statistics System.^22^ Relative risk functions linking alcohol use to cause-specific mortality risks were informed by published meta-analyses and secondary data analyses. Two distinct cause-specific relative risk functions were applied within the liver disease and cirrhosis category: one for chronic liver disease and cirrhosis, and a second for attenuated function for hepatitis C virus−related liver cirrhosis, reflecting the indirect alcohol-attributable pathway through disease progression (details available in eMethods, section 1.3.6, and eTable 6 in Supplement 1).

Data inputs for projecting forward are detailed in eMethods, section 1.3.4, in Supplement 1. In brief, total population growth and birth rates (from 2022 onward) were informed by the US Census Bureau’s population projections that uses census estimates of the resident population on July 1, 2022, as the base for modeling the US population forward.^23^ Migration rates (from 2022 onward) were projected forward accounting for year, sex, age, and race and ethnicity. Mortality (from 2024 onward) was projected forward using the US Census Bureau’s death projections.^23^

To account for disruptions during the COVID-19 pandemic, education transitions in 2020 to 2022 were parameterized using Panel Study of Income Dynamics 2019 to 2021 data (eMethods, section 1.3.5, in Supplement 1). We applied the same ordinal model to represent alcohol use transitions for all years given that secondary analyses of BRFSS data from 2020 onward did not reveal consistent directional changes in annual consumption. Progression through the ASBI care cascade—routine health care access, AS, and BI for hazardous drinkers—was informed by regression models built using individual-level data from the US National Survey on Drug Use and Health (NSDUH) from 2013 to 2019, accounting for heterogeneity across population subgroups (eMethods, section 1.3.5, in Supplement 1).^24^ Effect estimates are expected to be insensitive to pandemic-era adjustments, as both expansion and reference scenarios share identical adjustment assumptions, such that the projected differences are largely driven by the modeled expansion rather than the changes to baseline trends due to the pandemic.

#### Model Calibration and Validation

We used state-of-the-art calibration and validation techniques to refine and test the modeling of education and alcohol transitions, following a Bayesian probabilistic framework (eMethods, section 1.3.5, in Supplement 1). Calibration used ACS data (2000-2010) for education and BRFSS data (2000-2015) for alcohol use. Model performance was validated against later ACS (2011-2019) and BRFSS (2016-2019) data. Mortality risks are not calibrated; instead, reference mortality rates are adjusted to align mortality outcomes from the model with the US National Vital Statistics System data and US Census Bureau projections.

The validated model was used to estimate changes in hazardous alcohol use and YLL by sex, education, and race and ethnicity, accounting for uncertainty in model parameters. The final simulation comprised 70 parameters sets with 20 stochastic replications each (1400 runs per scenario). Outcomes are summarized as means with 95% CrI. The 95% CrI represents the 2.5th and 97.5th percentile of outcomes across the 70 parameter sets and reflects uncertainty in model inputs following calibration (eMethods, section 1.3.6, in Supplement 1).

### Expansion Scenarios

#### Modeling Flow

BI rates among past-year alcohol users were increased starting in 2025. For each step in the care cascade, individuals who would have received the service according to historical rates were first sampled using empirically informed regression models (refer to the Data Input section) before additional recipients were selected among the remaining individuals using the same model and according to the respective increase in each expansion scenario. Only individuals newly receiving BI due to expanded ASBI were assigned intervention effects because the effects of existing interventions are assumed to be already captured in historical alcohol use data. Both intervention effects are based on the most recent Cochrane review^9^: all newly intervened individuals received a reduction in GPD, sampled from the trial-level effect distribution (mean [SE] reduction: −2.86 [0.58] GPD) and scaled using each individual’s current level of consumption (eMethods, section 1.3.5, in Supplement 1). Among newly intervened individuals classified as heavy episodic drinkers, a proportion corresponding to the pooled risk difference (RD, −0.07; 95% CI, −0.02 to −0.12) were randomly selected to transition to non-HED status. GPD reduction and HED transition were sampled separately, such that HED status change was not determined by the magnitude of GPD reduction. Sampling of individuals who progress through the cascade was independent of the previous year’s sampling process, allowing individuals to be resampled in any year during the expansion period.

#### Expansion Strategies

Four expansion scenarios were modeled, each defined by annual increases in ASBI delivery. Scenario 1 increased screening among all past-year alcohol users by 20 million annually compared with historical rates (ie, an increase in the mean screened proportion from 55.7% to 66.9%). Scenario 1 was included to represent settings in which screening uptake is expanded without a commensurate expansion of BI delivery resources. In this scenario, total BI delivery still slightly increased as a larger pool of screened individuals means more individuals were identified as eligible for BI under existing delivery rates. Scenarios 2 and 3 increased screening by 20 million while simultaneously increasing BIs among screened individuals with hazardous alcohol use by 4 million and 8 million, respectively. Scenario 4 modeled the upper limit of ASBI’s impact by providing universal ASBI to all individuals accessing health care. All scenarios assumed continuation of historical trends of health care use (eMethods, section 1.3.5, in Supplement 1).

### Sensitivity Analyses

We investigated alternative parameter settings in 2 sensitivity analyses. First, we examined the impact of a lower (−1.71 GPD) or higher (−4.01) mean BI effect^9^ to gauge uncertainty in the treatment effect separately from other parameter variation. Second, in scenario 4, we extended eligibility for BI to include alcohol use category I (up to 20 GPD for women and 40 GPD for men, generally not considered hazardous use) to test whether results were sensitive to broader intervention coverage. Third, we conducted a sensitivity analysis to quantify the mortality cost of delaying expansion by comparing the total cumulative YLL averted over 2025 to 2030 under 4 implementation start dates: 2025 (original), 2026, 2027, and 2028.

## Results

### Screening and Brief Intervention Cascade by Subgroup

**Figure 1.**
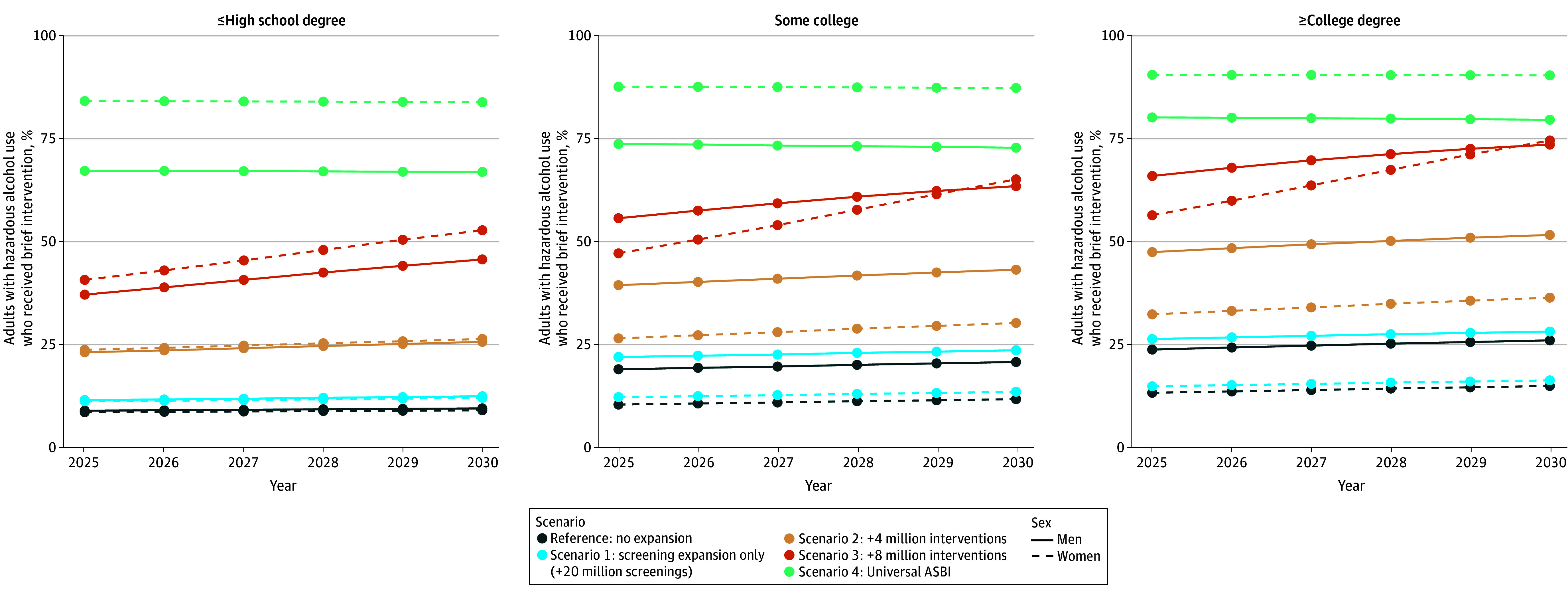
Line Graph of Simulated Mean Percentage of Adults With Hazardous Alcohol Use Who Received a Brief Intervention Each Year, by Sex and Education for Each Modeled Scenario Hazardous use was defined as more than 20 GPD for women and more than 40 GPD for men. Results by sex and race and ethnicity are presented in eFigure 1 in Supplement 1. ASBI indicates alcohol screening and brief intervention; GPD, grams of pure alcohol per day.

In the reference scenario, 18.0% (CrI, 10.4%-26.4%; 2.3 M; CrI, 1.3-3.3) of men and 12.2% (CrI, 5.8%-19.7%; 1.2 M; CrI, 0.5-1.9) of women with hazardous alcohol use were projected to receive a BI annually by 2030. Expanding the annual number of ASBI in primary care was simulated to have differential effects on the share of adults receiving a BI across population subgroups (Figure 1 and eFigure 1 in Supplement 1), consistent with the observed heterogeneity in the ASBI care cascade (eMethods, section 1.3.5, in Supplement 1).

### Changes in Hazardous Alcohol Use

**Figure 2.**
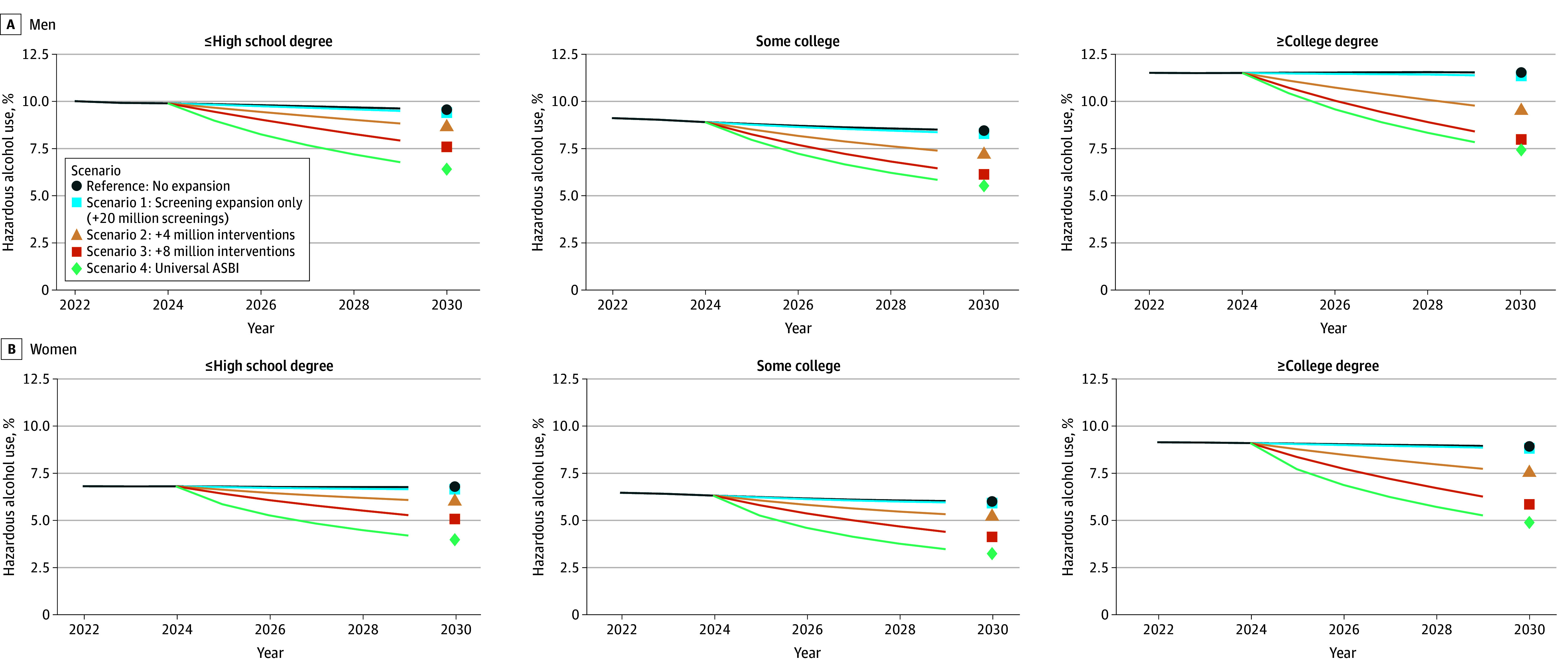
Line Graph of Simulated Mean Prevalence of Hazardous Alcohol Use, by Sex and Education for Each Modeled Scenario Hazardous use was defined as 20 GPD for women and 40 GPD for men. Results by sex and race and ethnicity are presented in eFigure 1, and for heavy episodic drinking prevalence, in eFigure 3 in Supplement 1. ASBI indicates alcohol screening and brief intervention; GPD, grams of pure alcohol per day.

Figure 2 shows simulated changes in the prevalence of hazardous alcohol use from 2022 to 2030 under the different expansion scenarios, by sex and education (see also eTable 1 in Supplement 1). Results by sex and race and ethnicity are presented in eFigure 2 and eTable 2 in Supplement 1. In the reference scenario, hazardous alcohol use is projected to remain relatively stable, with only slight decreases among men with less than or equal to high school or some college education.

Hazardous alcohol use was simulated to be most prevalent among those with college or higher degree (men, 11.6% [CrI, 11.5%-11.7%]; women, 8.9% [CrI, 8.8%-8.9%]) and White adults (men, 12.2% [CrI, 12.1%-12.4%]; women, 9.2% [CrI, 9.1%-9.4%]), and lowest among those with some college (men, 8.5% [CrI, 8.4%-8.6%]; women, 5.9% [CrI, 5.8%-6.0%]) and Black adults (men, 4.4% [CrI, 4.4%-4.5%]; women, 3.2% [CrI, 3.1%-3.3%]).

Across expansion scenarios, the magnitude of reductions in the prevalence of hazardous alcohol use by 2030 increased with the intensity of expansion. Screening expansion only (scenario 1) was simulated to have very small effects, whereas scenarios that include additional BIs (scenarios 2-4) show progressive reductions in hazardous alcohol use. Reductions were somewhat larger among men than women, and among White adults and those with higher education. Heavy episodic drinking followed a similar pattern, with absolute reductions by 2030 ranging from 0.4 (CrI, 0.3-0.5) percentage points among men with some college education to 1.4 (CrI, 1.3-1.5) percentage points among women with a college degree or more (eFigure 3, eTable 3-4 in Supplement 1).

### Changes in Years of Life Lost

In 2030, the annual mortality burden from key alcohol-related causes in the reference scenario was projected to be highest among those with high school or less education (men, 2642 YLL per 100 000 [CrI, 2539-2742]; women, 1068 [CrI, 1035-1112]) and Black adults (men, 2048 [CrI, 1979-2,103]; women, 853 [CrI, 828-876]), and lowest among those with college or more education (men, 825 YLL per 100 000 [CrI, 804-855]; women, 430 YLL per 100 000 [CrI, 414-447]), White men (1786 [CrI, 1755-1808]), and Hispanic women (659 [CrI, 632-693]).

**Figure 3.**
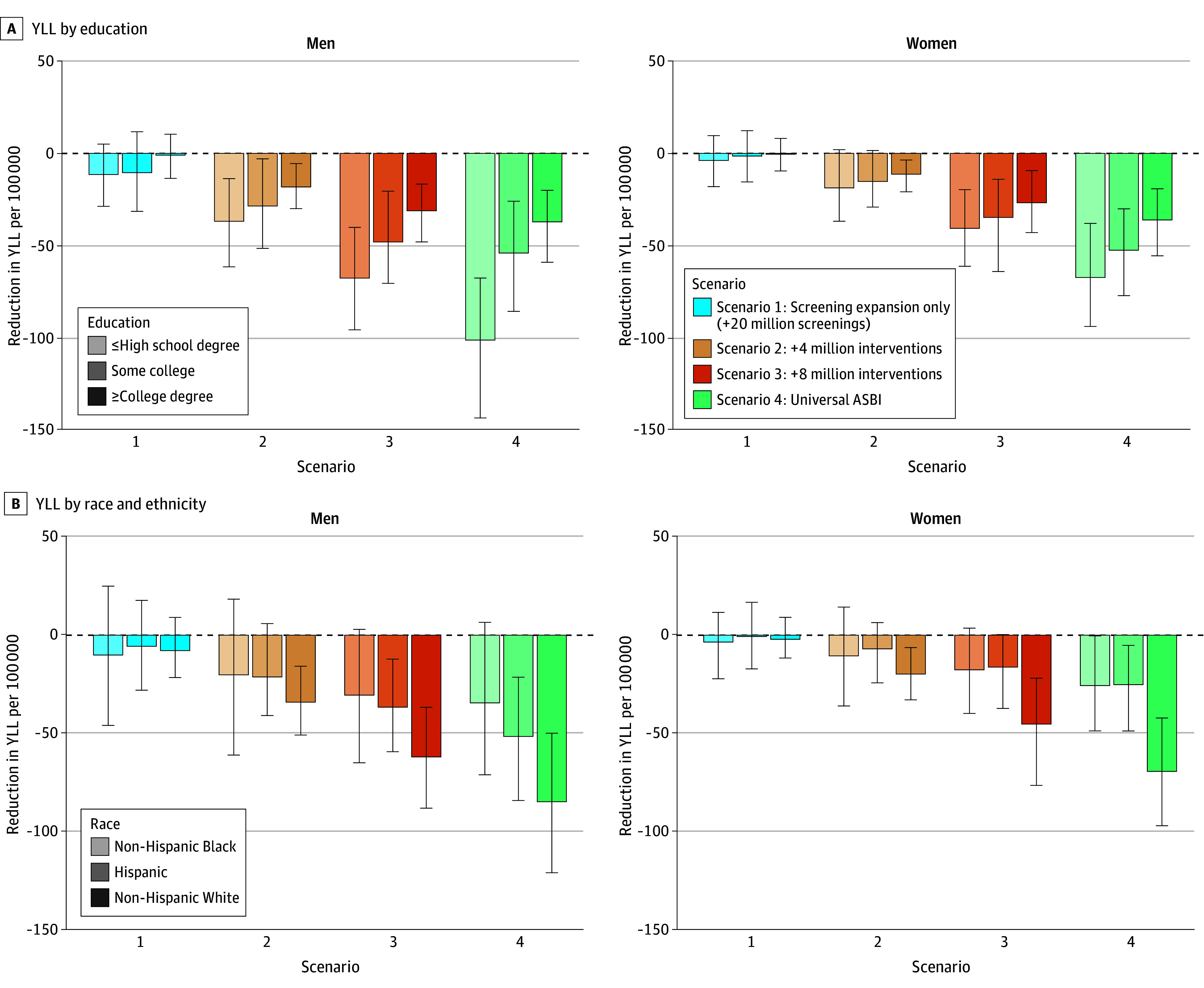
Bar and Whisker Chart of Simulated Mean Change in Annual Potential Years of Life Lost (YLL) per 100 000 From Key Alcohol-Related Causes for Each Modeled Expansion Scenario Compared to the Reference Scenario in 2030, Sex, Education, and Race and Ethnicity Errors bars represent 95% credible intervals across 70 unique parameter combinations. Full results are presented in eTables 6 and 7 in Supplement 1. Results are not reported for the other race and ethnicity category because model parameters could not be reliably informed by empirical data. Key alcohol-related causes of death are alcohol use disorder, liver disease and cirrhosis (including hepatitis C−related liver cirrhosis), motor-vehicle injuries, other unintentional injuries, and suicide. Results considering additional causes of deaths are presented in eTables 8 to 10 in Supplement 1. ASBI indicates alcohol screening and brief intervention.

Changes in annual YLL per 100 000 from alcohol-related causes under the different expansion scenarios by subgroup are shown in eTable 5 to 7 in Supplement 1. In men, the decrease compared with the reference scenario in 2030 was projected to be −8.0 YLL (CrI, −19.6 to 2.4) per 100 000 in scenario 1, −29.0 (CrI, −42.8 to −14.0) in scenario 2, −51.3 (CrI, −73.2 to −32.8) in scenario 3, and −68.9 (CrI, −102.2 to −44.8) in scenario 4. In women, the decrease is projected to be −1.9 YLL per 100 000 (CrI, −8.8 to 4.9) in scenario 1, −15.1 (CrI, −22.9 to −5.8) in scenario 2, −34.1 (CrI, −54.5 to −16.1) in scenario 3, and −52.0 (CrI, −75.0 to −30.2) in scenario 4 (eFigure 4 in Supplement 1). Figure 3 shows the simulated differences in 2030 across scenarios. In all expansion scenarios, those with a high school degree or less education benefit more than those with higher education in absolute terms, whereas the reverse applies in relative terms (eTable 6 in Supplement 1). White adults benefit more than Black and Hispanic adults, among both sexes in absolute and relative terms (eTable 7 in Supplement 1).

### Potential Impact on the Socioeconomic Gap

**Figure 4.**
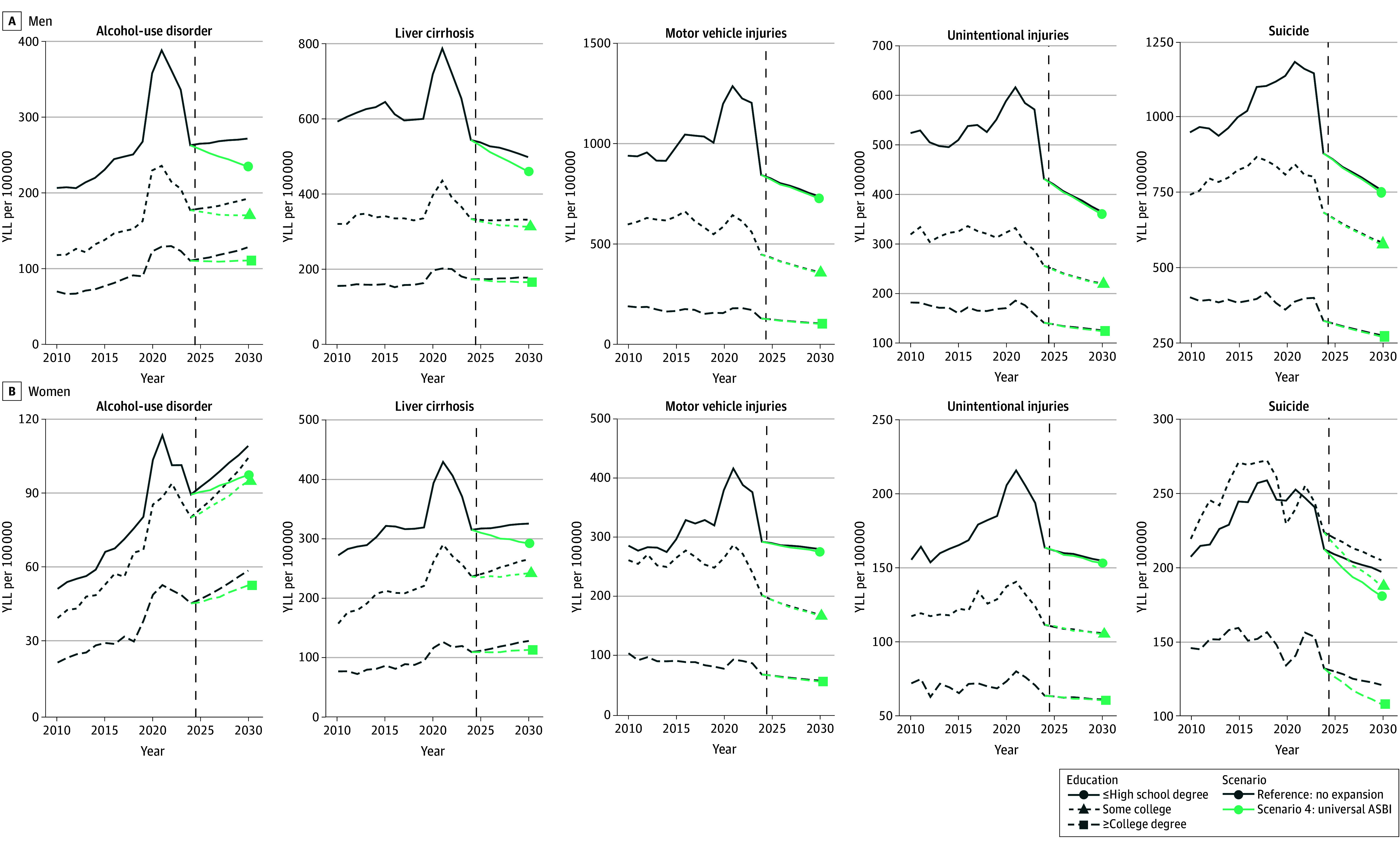
Line Graph of Simulated Mean Potential Years of Life Lost (YLL) per 100 000 by Sex and Education Ranges vary by group and cause of death. Dashed vertical line at 2025 indicates the start of ASBI expansion. Results considering additional causes of deaths are presented in eTables 8 to 12 in Supplement 1. ASBI indicates alcohol screening and brief intervention.

**Figure 5.**
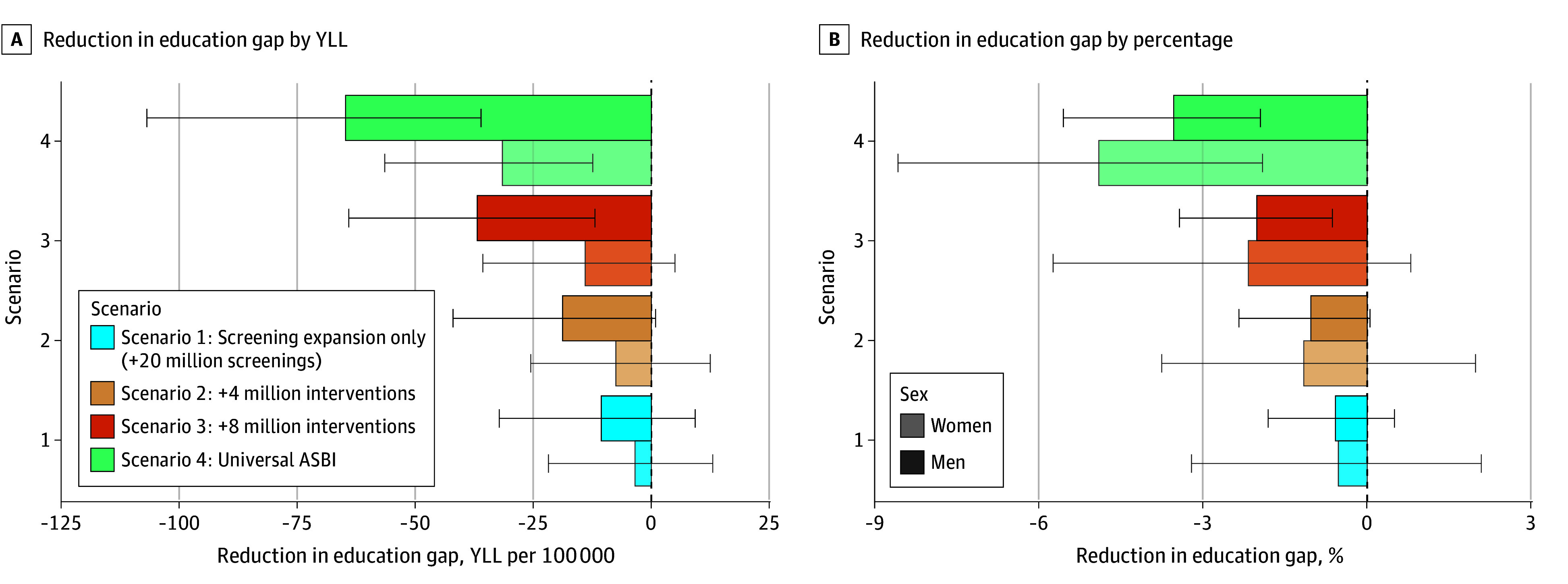
Bar and Whisker Chart of Simulated Mean Reduction in the Gap in Combined Potential Years of Life Lost (YLL) per 100 000 From Key Alcohol-Related Causes Between Adults With High School Degree or Less Compared With Those With a College Degree or More in 2030, Stratified by Sex Errors bars represent 95% credible intervals across 70 unique parameter combinations. ASBI indicates alcohol screening and brief intervention.

The decrease in YLL per 100 000 was projected to be most pronounced for causes with the highest alcohol-attributable fractions, that is, AUD and liver disease and cirrhosis (Figure 4; eTable 8 in Supplement 1). Simulation results further indicate that those with high school or less education will experience the largest declines: In scenario 4, annual YLL per 100 000 attributable to liver disease and cirrhosis in 2030 decreased by −38.5 (CrI, −81.4 to −11.8) for men and by −33.3 (CrI, −53.4 to −16.8) for women with high school or less, but only by −11.9 (CrI, −26.8 to 0.5) for men and by −15.1 (CrI, −25.9 to −6.0) for women with college or more (eTable 10 in Supplement 1). Figure 5 illustrates the potential narrowing of the alcohol-related mortality gap in 2030 between those with low (≤ high school) and high (≥ college) education across all modeled causes. Among men, the gap consistently narrowed in scenario 3 by −2.0% (−36.7 YLL per 100 000 [CrI, −63.8 to −11.9]) and in scenario 4 by 3.5% (−64.5 YLL per 100 000 [CrI, −106.4 to −35.9]). Among women, a consistent narrowing of the gap was only observed in scenario 4 by −4.9% (−31.4 YLL per 100 000 [CrI, −56.2 to −12.4]).

### Sensitivity Analyses

First, varying the mean treatment effect of additional BIs considerably affected the projected impact of expansion. Under scenario 4, the decrease among men with a high school or less ranged between −132.8 YLL per 100 000 (CrI, −194.7 to −87.1) with a higher mean effect and −64.8 YLL per 100 000 (CrI, −91.6 to −35.6) with a lower mean effect (eFigure 5 and eTable 11-14 in Supplement 1). Second, extending universal ASBI in scenario 4 to include alcohol use category I had very little impact on the projected population-level effects (eTable 5 and eTable 15 in Supplement 1). Third, total cumulative YLL averted over 2025 to 2030 decreased with each year of delayed expansion (eFigure 6 and eTable 16 in Supplement 1). Under scenario 3, delaying the start of expansion from 2025 to 2026 (1-year delay), 2027 (2-year delay), or 2028 (3-year delay) was associated with a cumulative loss of 47.0 YLL per 100 000 (CrI, 18.3-75.3), 92.1 (CrI, 55.3-134.4), and 116.8 (CrI, 80.2-178.2) in men and 30.9 (CrI, 5.7-54.0), 63.5 (CrI, 32.6-100.6), and 82.5 (CrI, 41.6-122.8) in women by 2030, which quantifies the mortality cost of deferred action.

## Discussion

This microsimulation study estimated that expanding ASBI in primary care through 2030 could lower hazardous drinking and thereby annually save up to 68.9 (CrI, 44.8-102.2) and 52.0 (CrI, 30.2-75.0) YLL per 100 000 in men and women, respectively, due to key causes of deaths associated with alcohol use. Reductions in hazardous alcohol use were projected to be more pronounced among adults with a college degree or more (than those with lower educational attainment), and among White adults (than Black and Hispanic adults), likely driven by their higher baseline consumption and more regular health care visits.^25^

Overall, the simulations indicated that only substantial investments in BI expansion (eg, 8 million additional annual BI in scenario 3) would yield meaningful declines in YLL. This would correspond to approximately 3.6 times as many BIs as simulated under the reference scenario in 2025, with uncertainty driven by baseline coverage estimates derived from self-report in NSDUH data (reference, 3.1 million [CrI, 1.8-4.8] vs scenario 3: 11.1 million [CrI, 9.8-12.8]; ratio 3.6 [CrI, 2.7-5.4]). However, community implementation of ASBI remains limited due to persistent barriers, including time constraints and competing priorities in primary care, limited training and confidence among clinicians, inadequate reimbursement, and fragmented workflows, as documented by a comprehensive body of existing literature.^26,27,28^ These challenges, while beyond the scope of this analysis, highlight the importance of implementation strategies that integrate ASBI into standard primary care processes. Recent evidence from a multisite Agency for Healthcare Research and Quality initiative^29,30,31^ provides important context for the feasibility of our expansion scenarios, showing that structured practice support increased the proportion of patients screened for unhealthy alcohol use from 23.2% to 35.8% and those receiving brief counseling from 20.1% to 27.6% across project sites.^32^ While these gains are encouraging, the scale of expansion modeled in our scenario 3 would demand broader system-level implementation than has yet been demonstrated in any single initiative.

A sensitivity analysis further illustrates the urgency of action: delaying roll-out of scenario 3 by 3 years (to 2028) was projected to result in 116.8 and 82.5 YLL foregone per 100 000 men and women by 2030, suggesting that the value of timely action is sizeable even within a relatively short implementation window. Another practically relevant finding is that expanding screening alone under stagnant BI delivery rates (scenario 1) produced YLL reductions that were statistically indistinguishable from zero in both men and women. This finding suggests that screening rates alone are an inadequate proxy for the quality and impact of ASBI programs.

With respect to education-based socioeconomic status and mortality, the model projected the largest absolute decreases in YLL per 100 000 among those with high school or less education, a group with a disproportionately high alcohol-attributable burden. This pattern—comparatively larger gains in YLL despite smaller reductions in hazardous drinking—is consistent with the alcohol harm paradox: individuals with lower socioeconomic status experience greater adverse health consequences at comparable or even lower levels of consumption.^33^ This paradox is thought to result from compounding structural and contextual factors including co-occurring risk factors such as smoking or illicit drug use, greater exposure to social and environmental stressors, higher prevalence of comorbid conditions, and lower health care access.^34^

Despite these larger absolute gains, ASBI expansion had measurable but modest equity-correcting potential: under universal ASBI delivery, the YLL gap between those with high school or less and college or more education narrowed by only 3.5% in men and 4.9% in women. ASBI scale-up alone is therefore unlikely to eliminate the socioeconomic inequalities in key causes associated with alcohol use. Structural barriers to health care access and broader social determinants of health remain important constraints, even under ambitious ASBI expansion. Complementary policies that improve primary care access and affordability for disadvantaged populations and address the broader social determinants of health will be needed to substantially narrow these inequalities.

### Limitations

Our modeling is based on several key assumptions; as such, the following limitations need to be acknowledged. First, the ASBI care cascade was informed by regression models built on cross-sectional NSDUH data, where alcohol use and ASBI receipt are self-reported and subject to biases, most importantly underreporting of level and patterns of use. ASBI receipt was operationalized using the survey item on self-reported receipt of advice from a physician to cut down on drinking, which is subject to recall bias and may capture heterogeneous clinical interactions, ranging from brief, directive advice to reduce alcohol use to more structured BIs that incorporate motivational techniques and patient-led goal setting.

Second, socioeconomic status was represented only by educational attainment without considering employment or income due to high missingness in input data. Third, race and ethnicity analyses exclude the other category, which combines American Indian and Alaska Native, Asian, and Native Hawaiian or Other Pacific Islander individuals, due to small sample sizes, high heterogeneity, and high racial misclassification rates for American Indian and Alaska Native on death certificates.^35^ This aggregation can obscure important disparities, particularly given that American Indian and Alaska Native communities experience the highest alcohol-attributable mortality rates of any US population group.^36^ The potential equity implications of ASBI expansion for American Indian and Alaska Native communities therefore could not be examined.

Fourth, modeled causes of death do not include alcohol-related cancers, which contribute substantially to alcohol-attributable mortality. Because these cancers are characterized by substantial latency between exposure and disease manifestation,^37^ they cannot be adequately represented within the present modeling framework based on annual drinking transitions and comparatively short-term follow-up; our estimates therefore capture only short- and intermediate-term impacts of ASBI expansion.

Fifth, projections into the future inherently involve uncertainty. Although we used US Census Bureau population and mortality projections as benchmarks, these do not probabilistically quantify uncertainty, and all projections assume that no external events or interventions alter model parameters. Long-term effects from the COVID-19 pandemic may also not be fully captured.

Sixth, we assumed a homogenous BI effect scaled only by baseline consumption level because evidence was insufficient to parameterize subgroup-specific effects.^9^ While BI effectiveness may vary across demographic groups,^5,38^ differential access to ASBI—which is empirically substantiated and modeled across subgroups—may be at least as important for differential population-level impact as treatment effect heterogeneity.

Lastly, we did not model referral to specialized alcohol treatment or pharmacotherapy for AUD (eg, naltrexone, acamprosate),^39^ both of which address individuals with the heaviest alcohol use (and likely addiction) and are, therefore, expected to have a meaningful impact on mortality. However, evidence that ASBI reliably increases specialized alcohol care is inconsistent,^40,41^ which is why modeling these pathways was beyond the scope of this study and remain important avenues for future work.

## Conclusions

This decision analytical study shows that expanding AS and, particularly, BI in primary care has the potential to decrease mortality from key causes of death associated with alcohol use. Although the largest absolute YLL reductions were projected among lower−socioeconomic status groups, ASBI expansion alone is unlikely to eliminate socioeconomic inequalities in alcohol-attributable mortality. Concerted investment and effort by payers and health care professionals will be needed to routinely integrate evidence-based ASBI into standard primary care workflows. If combined with policies that increase primary care access generally, expanded ASBI would reach even more US residents and likely achieve greater reductions in mortality across diverse population subgroups.

## Notes

Supplement 1. eMethods 1. ODD Protocol eTable 1. Simulated prevalence of alcohol use categories by sex and education (standard effect) eTable 2. Simulated prevalence of alcohol use categories by sex and race and ethnicity (standard effect) eTable 3. Simulated prevalence of heavy episodic drinking by sex and education (standard effect) eTable 4. Simulated prevalence of heavy episodic drinking by sex and race and ethnicity (standard effect) eTable 5. Simulated combined YLL per 100,000 by sex eTable 6. Simulated combined YLL per 100,000 by sex and education (standard effect) eTable 7. Simulated combined YLL per 100,000 by sex and race and ethnicity (standard effect) eTable 8. Simulated YLL per 100,000 by sex and cause (standard effect) eTable 9. Simulated YLL per 100,000 by sex comparing different liver disease aetiologies (standard effect) eTable 10. Simulated YLL per 100,000 by sex, education, and cause (standard effect) eTable 11. Simulated combined YLL per 100,000 by sex and education (sensitivity analysis 1, maximum effect) eTable 12. Simulated combined YLL per 100,000 by sex and education (sensitivity analysis 1, minimum effect) eTable 13. Simulated combined YLL per 100,000 by sex and race and ethnicity (sensitivity analysis 1, maximum effect) eTable 14. Simulated combined YLL per 100,000 by sex and race and ethnicity (sensitivity analysis 1, minimum effect) eTable 15. Simulated combined YLL per 100,000 (sensitivity analysis 2) eTable 16. Cumulative YLL reductions per 100,000 across the 2025–2030 period (sensitivity analysis 3) eFigure 1. Simulated percentage of adults with hazardous alcohol use who received a brief intervention by sex and race and ethnicity eFigure 2. Simulated mean prevalence of hazardous alcohol use by sex and race and ethnicity eFigure 3. Simulated mean change in heavy episodic drinking prevalence by subgroup eFigure 4. Simulated mean change in YLL per 100,000 by sex. eFigure 5. Simulated mean change in combined YLL per 100,000 by sex, education and average brief intervention effect (sensitivity analysis 1) eFigure 6. Cumulative changes in YLL per 100,000 by sex across the 2025–2030 period (sensitivity analysis 3)

Supplement 2. Data Sharing Statement
