# Supplementary material for: Expanded Alcohol Screening and Brief Intervention to Address Premature Mortality
Source: JAMA Health Forum. 2026 Jul 31;7(7):e262348. doi: 10.1001/jamahealthforum.2026.2348 (PMC13428287; doi:10.1001/jamahealthforum.2026.2348)
Supplement: Supplement 1. — eMethods 1. ODD Protocol eTable 1. Simulated prevalence of alcohol use categories by sex and education (standard effect) eTable 2. Simulated prevalence of alcohol use categories by sex and race and ethnicity (standard effect) eTable 3. Simulated prevalence of heavy episodic drinking by sex and education (standard effect) eTable 4. Simulated prevalence of heavy episodic drinking by sex and race and ethnicity (standard effect) eTable 5. Simulated combined YLL per 100,000 by sex eTable 6. Simulated combined YLL per 100,000 by sex and education (standard effect) eTable 7. Simulated combined YLL per 100,000 by sex and race and ethnicity (standard effect) eTable 8. Simulated YLL per 100,000 by sex and cause (standard effect) eTable 9. Simulated YLL per 100,000 by sex comparing different liver disease aetiologies (standard effect) eTable 10. Simulated YLL per 100,000 by sex, education, and cause (standard effect) eTable 11. Simulated combined YLL per 100,000 by sex and education (sensitivity analysis 1, maximum effect) eTable 12. Simulated combined YLL per 100,000 by sex and education (sensitivity analysis 1, minimum effect) eTable 13. Simulated combined YLL per 100,000 by sex and race and ethnicity (sensitivity analysis 1, maximum effect) eTable 14. Simulated combined YLL per 100,000 by sex and race and ethnicity (sensitivity analysis 1, minimum effect) eTable 15. Simulated combined YLL per 100,000 (sensitivity analysis 2) eTable 16. Cumulative YLL reductions per 100,000 across the 2025–2030 period (sensitivity analysis 3) eFigure 1. Simulated percentage of adults with hazardous alcohol use who received a brief intervention by sex and race and ethnicity eFigure 2. Simulated mean prevalence of hazardous alcohol use by sex and race and ethnicity eFigure 3. Simulated mean change in heavy episodic drinking prevalence by subgroup eFigure 4. Simulated mean change in YLL per 100,000 by sex. eFigure 5. Simulated mean change in combined YLL per 100,000 by sex, education and averag [file jamahealthforum-e262348-s001.pdf]

Supplemental Online Content

Lemp JM, Kilian C, Kou X, et al. Expanded alcohol screening and brief intervention to address premature mortality. *JAMA Health Forum.* Published online July 31, 2026. doi:10.1001/jamahealthforum.2026.2348

eMethods 1. ODD Protocol

eTable 1. Simulated prevalence of alcohol use categories by sex and education (standard effect)

eTable 2. Simulated prevalence of alcohol use categories by sex and race and ethnicity (standard effect)

eTable 3. Simulated prevalence of heavy episodic drinking by sex and education (standard effect)

eTable 4. Simulated prevalence of heavy episodic drinking by sex and race and ethnicity (standard effect)

eTable 5. Simulated combined YLL per 100,000 by sex

eTable 6. Simulated combined YLL per 100,000 by sex and education (standard effect)

eTable 7. Simulated combined YLL per 100,000 by sex and race and ethnicity (standard effect)

eTable 8. Simulated YLL per 100,000 by sex and cause (standard effect)

eTable 9. Simulated YLL per 100,000 by sex comparing different liver disease aetiologies (standard effect)

eTable 10. Simulated YLL per 100,000 by sex, education, and cause (standard effect)

eTable 11. Simulated combined YLL per 100,000 by sex and education (sensitivity analysis 1, maximum effect)

eTable 12. Simulated combined YLL per 100,000 by sex and education (sensitivity analysis 1, minimum effect)

eTable 13. Simulated combined YLL per 100,000 by sex and race and ethnicity (sensitivity analysis 1, maximum effect)

© 2026 Lemp, JM et al. JAMA Health Forum.

eTable 14. Simulated combined YLL per 100,000 by sex and race and ethnicity (sensitivity analysis 1, minimum effect)

eTable 15. Simulated combined YLL per 100,000 (sensitivity analysis 2)

eTable 16. Cumulative YLL reductions per 100,000 across the 2025–2030 period (sensitivity analysis 3)

eFigure 1. Simulated percentage of adults with hazardous alcohol use who received a brief intervention by sex and race and ethnicity

eFigure 2. Simulated mean prevalence of hazardous alcohol use by sex and race and ethnicity

eFigure 3. Simulated mean change in heavy episodic drinking prevalence by subgroup

eFigure 4. Simulated mean change in YLL per 100,000 by sex.

eFigure 5. Simulated mean change in combined YLL per 100,000 by sex, education and average brief intervention effect (sensitivity analysis 1)

eFigure 6. Cumulative changes in YLL per 100,000 by sex across the 2025–2030 period (sensitivity analysis 3)

This supplemental material has been provided by the authors to give readers additional information about their work.

© 2026 Lemp, JM et al. JAMA Health Forum.

Table of Contents

eMethods 1. ODD Protocol....3

1.1 Overview....3

1.1.1 Purpose and patterns....3

1.1.2 Entities, state variables and scales....3

1.1.3 Process overview & scheduling....7

1.2 Design concepts....10

1.2.1 Stochasticity....10

1.2.2 Observation....11

1.3 Details....12

1.3.1 Initialisation....12

1.3.2 Input data....12

1.3.3 Race and ethnicity coding in individual data sources....15

1.3.4 Modelling forward in time....16

1.3.5 Sub-models....19

HED....19

Mortality....19

Updating educational attainment....22

Updating alcohol consumption....28

Births and migration....32

Applying screening and brief intervention expansion....33

1.3.6 Uncertainty representation....38

1.3.7 Model assumptions....44

1.4 References....45

eTable 1. Simulated prevalence of alcohol use categories by sex and education (standard effect)....49

eTable 2. Simulated prevalence of alcohol use categories by sex and race and ethnicity (standard effect)....51

eTable 3. Simulated prevalence of heavy episodic drinking by sex and education (standard effect)....53

eTable 4. Simulated prevalence of heavy episodic drinking by sex and race and ethnicity (standard effect)....54

eTable 5. Simulated combined YLL per 100,000 by sex....55

© 2026 Lemp, JM et al. JAMA Health Forum.

eTable 6. Simulated combined YLL per 100,000 by sex and education (standard effect). 56
eTable 7. Simulated combined YLL per 100,000 by sex and race and ethnicity (standard effect). 57
eTable 8. Simulated YLL per 100,000 by sex and cause (standard effect). 58
eTable 9. Simulated YLL per 100,000 by sex comparing different liver disease aetiologies (standard effect). 61
eTable 10. Simulated YLL per 100,000 by sex, education, and cause (standard effect). 62
eTable 11. Simulated combined YLL per 100,000 by sex and education (sensitivity analysis 1, maximum effect). 68
eTable 12. Simulated combined YLL per 100,000 by sex and education (sensitivity analysis 1, minimum effect). 69
eTable 13. Simulated combined YLL per 100,000 by sex and race and ethnicity (sensitivity analysis 1, maximum effect). 70
eTable 14. Simulated combined YLL per 100,000 by sex and race and ethnicity (sensitivity analysis 1, minimum effect). 71
eTable 15. Simulated combined YLL per 100,000 (sensitivity analysis 2). 72
eTable 16. Cumulative YLL reductions per 100,000 across the 2025–2030 period (sensitivity analysis 3). 73
eFigure 1. Simulated percentage of adults with hazardous alcohol use who received a brief intervention by sex and race and ethnicity. 74
eFigure 2. Simulated mean prevalence of hazardous alcohol use by sex and race and ethnicity. 75
eFigure 3. Simulated mean change in heavy episodic drinking prevalence by subgroup. 76
eFigure 4. Simulated mean change in YLL per 100,000 by sex. 77
eFigure 5. Simulated mean change in combined YLL per 100,000 by sex, education and average brief intervention effect (sensitivity analysis 1). 78
eFigure 6. Cumulative changes in YLL per 100,000 by sex across the 2025–2030 period (sensitivity analysis 3). 79

This supplementary material has been provided by the authors to give readers additional information about their work.

© 2026 Lemp, JM et al. JAMA Health Forum.

eMethods 1. ODD Protocol

1.1 Overview

1.1.1 Purpose and patterns

The purpose of the microsimulation is to estimate the effects of alcohol control policies on alcohol consumption levels, mortality rates, and health inequalities within population subgroups. A description of the overall study design for the Simulation of Alcohol Control Policies (SIMAH) project can be found in Probst et al. 2023.[1] In the following, we provide full details using the Overview, Design concepts and Details (ODD) protocol for the description of simulation models.[2]

A description of the full study protocol for the SIMAH project can be found here: Probst, C., Buckley, C., Lasserre, A. M., Kerr, W. C., Mulia, N., Puka, K., ... & Rehm, J. (2023). Simulation of Alcohol Control Policies for Health Equity (SIMAH) project: study design and first results. American journal of epidemiology, 192(5), 690-702.

1.1.2 Entities, state variables and scales

The entities in the model are simulated individuals that are representative of the adult US population. The model is designed to be representative over the time period 2000 to 2021 with model projections from 2022 to 2030. Individuals in the micro-synthetic population are characterised by both static and time-varying attributes, as listed in Table S1. Baseline attributes assigned at the initialisation year (i.e., 2000) include age, sex, race and ethnicity, and educational attainment, as well as information on their alcohol use including drinking status (current drinker, abstainer, former drinker) and average grams of pure alcohol consumed per day (i.e., level of consumption). As the simulation progresses, additional attributes are dynamically assigned. These include policy-relevant variables (e.g., beverage-specific alcohol use), cause-specific mortality relative risk, and an indicator identifying heavy episodic drinking status (HED). Each time step of the model represents one year.

© 2026 Lemp, JM et al. JAMA Health Forum.

Table S1. Properties of synthetic individuals.

Parameter	Values	Data source(s)	Update rules
Age	18-79 (individual years)	The age, sex, race and ethnicity and education distributions were assigned based on the Census and American Community Survey (2000-2021)[3]	Age increases by +1 year at each time step
Sex	Male, Female[a]		Static properties
Race and ethnicity	non-Hispanic White, non-Hispanic Black, Hispanic, Other[b]		
Educational attainment	High school degree or less, Some college, College degree or more		Individuals aged 18-34 years have the opportunity to update their educational attainment level at each time step. Markov model, transition probabilities conditional on age, sex, and race and ethnicity (described in 1.3.5 Sub-models)[4]
Drinking status	Current drinker, Former drinker, Abstainer	2005 National Alcohol Survey data on lifetime abstainer, past year abstainer, past 30-day abstainer and current drinker were used to adjust past 30-day drinking from annual Behavioural Risk Factor Surveillance System (BRFSS) data (2000 to 2019) to past 12 months drinking and to add the category of former drinkers, using distributions from subgroups based on age, sex, race and ethnicity. Further details are described in a published report.[5]	Current drinker and abstainer are assigned based on alcohol updating functions. Former drinker is assigned separately by sampling from within abstainer category.

© 2026 Lemp, JM et al. JAMA Health Forum.

Alcohol use category	Non-drinker, Category 1, Category 2, Category 3	BRFSS 2000 to 2019 data on quantity and frequency of alcohol use in past 30 days were used to calculate average grams of pure alcohol consumed per day (g per day). National- and state-level alcohol per capita consumption data were used to correct for underreporting of alcohol use in survey data, see [6] for further details. Discrete alcohol use categories are non-drinker, category 1 with up to 20 g per day for women and up to 40 g per day for men, category 2 with more than 20 g per day up to 40 g per day for women and more than 40 g per day up to 60 g per day for men, and category 3 with more than 40 g per day for women and more than 60 g per day for men.[6]	All individuals have the opportunity to update at each time step. This is based on an ordinal logistic regression conditional on age, sex, race and ethnicity, educational attainment, and previous drinking (see 1.3.5 Sub-models). For our preliminary work on alcohol transitions, see [7].
Alcohol consumption level	Average grams of pure alcohol consumed per day, 0-200 (capped)		Re-assigned every year in individuals who changed their alcohol use category based on distributions of alcohol consumption levels by alcohol use category and individual characteristics in the BRFSS data (see 1.3.5 Sub-models).
Cause-specific relative mortality risk	Continuous	Systematic reviews, meta-analyses and secondary analyses (see Table SX).	Reassigned every year based on alcohol use and other variables as applicable (see 1.3.5. Sub-models).
Binary indicator for HED	0 = non-HED; 1= HED	BRFSS 2011 to 2022 data on past 30-day heavy episodic drinking.	Individuals consuming ≥60 g per day are classified as HED (value = 1). Individuals consuming <1 g per day are classified as non-HED (value = 0). Individuals consuming 1-60 g per day are probabilistically assigned to either HED or non-HED status at each time step based on three XGBoost-based classification models conditional on average alcohol consumption (g/day), age, sex, race and ethnicity, and educational attainment (see 1.3.5 Sub-models).

© 2026 Lemp, JM et al. JAMA Health Forum.

Note: [a] The variable labeled sex in data sources (e.g. male/female in ACS and in BRFSS data) is self-reported and at least partially corresponds to gender identity; therefore, we refer to these groups as men and women throughout, consistent with prior publications and common public health reporting conventions. [b] The coding of race and ethnicity differs between different data sources and is summarised in section 1.3.3.

Mortality is accounted for in two ways in the simulation. Alcohol-related causes (Table S2) are modelled through risk functions describing the relationship between alcohol-use and cause-specific risk of mortality. The remaining causes ("all-cause mortality") are based on mortality data from CDC's National Vital Statistics System (NVSS).[9] The process on how individuals are assigned mortality risks is described below (see Table S3 and section 1.3.4. Sub-models for details).

Table S2. Causes of death modelled through alcohol-related risk factors.

**Variable name**	**Cause of death**	**ICD-10 codes**
AUD	Alcohol use disorder	E24.4, F10-F10.9, G62.1, G31.2, G72.1, I42.6, K29.2, K85.2, K86.0, R78.0, X45-X45.9[a], Y15-Y15.9, Y90, Y91
DM	Diabetes	E10-E14
HLVDC	Hepatitis-related liver cirrhosis	B18, B17.1, B92.4[b]
LVDC	Liver cirrhosis ("main pathway")	K70, K73, K74, K76.0, K76.6
HYPHD	Hypertensive heart disease	I11
IHD	Ischemic heart disease	I20-I25
ISTR	Ischemic stroke	G45-G46.8, I63-I63.9, I65-I66.9, I67.2-I67.848, I69.3-I69.4
IJ	Intended injury (Suicide)	X60-X84, Y87.0
MVACC	Injuries from motor vehicle accidents	V02-V04, V09.0, V09.2, V12-V14, V19.0-V19.2, V19.4-V19.6, V20-V79, V80.3-V80.5, V81.0-V81.1, V82.0-V82.1, V83-V86, V87.0-V87.8, V88.0-V88.8, V89.0, V89.2
UIJ	Other unintentional Injury	All codes in V01-X59, Y40-Y86, Y88-Y89 excluding motor vehicle accidents

Note: [a] Alcohol poisoning is included as part of Alcohol Use Disorder (instead of unintentional injury). [b] B17.1 and B92.4 were included based on a technicality rather than a content-level decision. Around 2004-2005, coding practices shifted, and deaths involving Hepatitis C began to be coded as chronic hepatitis (B18.2) rather than acute hepatitis (B17.1) or sequelae of viral hepatitis (B92.4). Death from acute hepatitis is very rare, as the immune system usually clears the virus; infections present for six months or more are coded as chronic. Although the duration of infection cannot be determined with certainty at death, antibody patterns offer some indication of chronicity.

© 2026 Lemp, JM et al. JAMA Health Forum.

1.1.3 Process overview & scheduling

Figure S1 depicts the process overview. The model proceeds in discrete annual time steps. The micro-synthetic population is initialised in the year 2000. Each year, the following process takes place:

1) Update HED status conditional on average daily alcohol consumption, age, sex, race and ethnicity, and education. For individuals whose alcohol consumption falls between 1 and 60 g per day, HED status is probabilistically assigned using three XGBoost models, whereas assignment for all other individuals is deterministic based on drinking status and/or average alcohol consumption.
2) If the policy is active in a given year, apply the policy effect according to its policy-specific mechanism (see below);
3) Record the current micro-synthetic population and its properties, including alcohol consumption (i.e., alcohol use category, alcohol consumption level);
4) Individuals are removed from the population in two steps: (i) for causes that are not explicitly being modelled through alcohol-related risk functions (“all-cause mortality”), individuals are randomly removed in proportion to mortality rates by age, sex, race and ethnicity, and educational attainment. (ii) for explicitly modelled alcohol-related causes, individuals are randomly removed in proportion to mortality rates by age, sex, race and ethnicity, educational attainment and cause-specific relative risks conditional on their alcohol use;
5) Record the number of deaths per cause, calculate Years of Potential Life Lost (YLL);
6) Update educational attainment conditional on age, sex, race and ethnicity, this happens for all individuals aged 18-34 and is controlled through a Markov Model;
7) Update alcohol use category conditional on age, sex, race and ethnicity, and education, this happens for all individuals and is controlled through an ordinal logistic regression;
8) If individuals have changed their alcohol use category, update alcohol consumption level (g per day) based on empirical distributions estimated from the Behavioral Risk Factor Surveillance System (BRFSS) data conditional on alcohol use category and age, sex, race and ethnicity, and educational attainment;
9) Age all individuals by +1 year, remove all individuals that are older than 79 years of age; and
10) Add and remove individuals due to birth (18-year-olds) and migration based on BRFSS data.

© 2026 Lemp, JM et al. JAMA Health Forum.

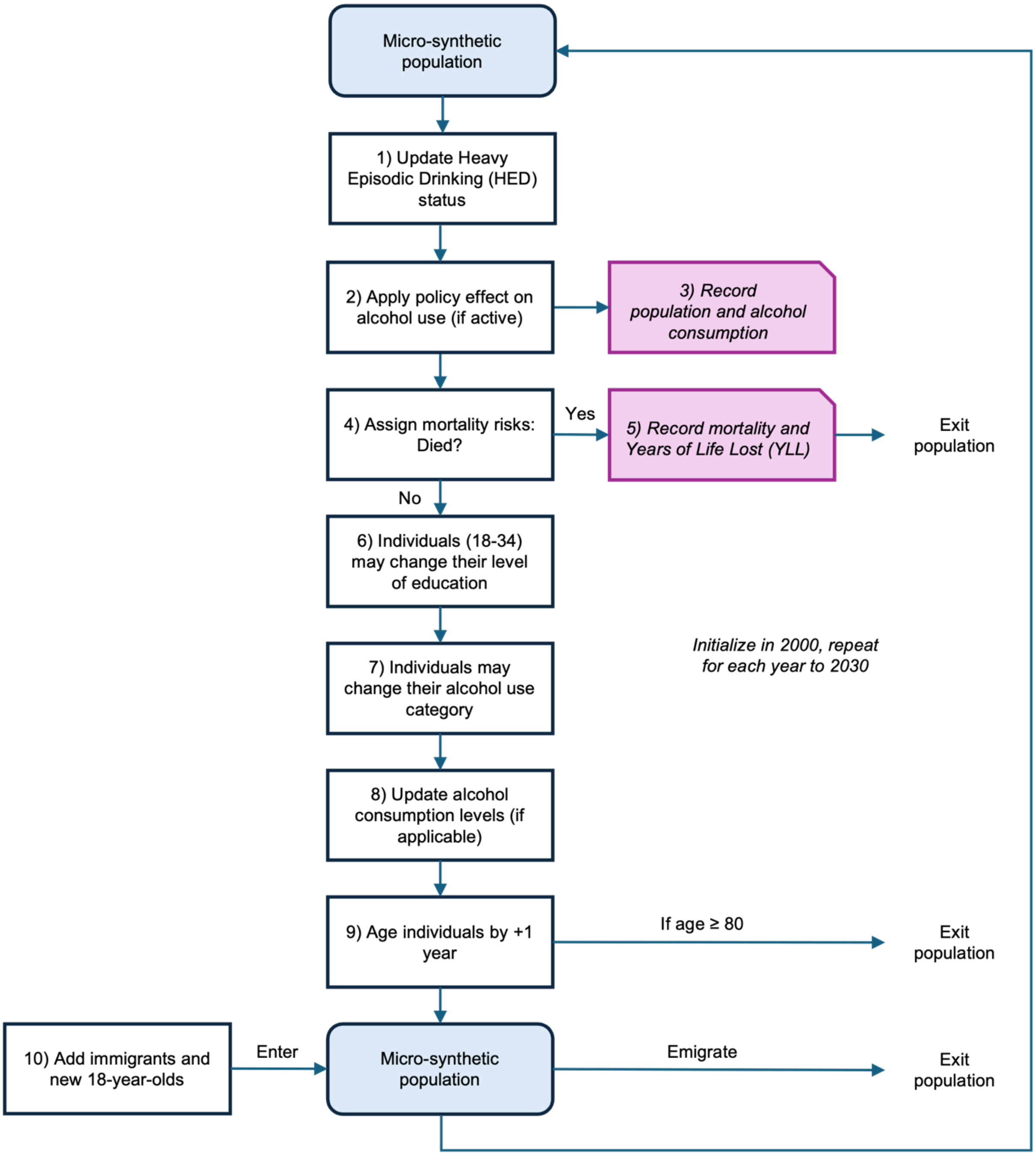

Figure S1. Process overview.

In each year that a given policy is active, the policy effect is applied before recording alcohol consumption, so that the policy effect is measurable within the same year (i.e., immediate policy effect). The screening and brief intervention expansion modelling process comprises six steps:

a) Assign empirically informed probabilities of having a routine health visit to all individuals who currently drink alcohol, based on National Survey on Drug Use and Health (NSDUH) data and conditional on alcohol use category and age, sex, race and ethnicity, and educational attainment;
b) Assign empirically informed probabilities of being screened for hazardous alcohol use to all individuals who were sampled as having had a routine health visit, based on

© 2026 Lemp, JM et al. JAMA Health Forum.

NSDUH data and conditional on alcohol use category and age, sex, race and ethnicity, and educational attainment;
c) Assign empirically informed probabilities of being screened for alcohol use to additional individuals (N determined by expansion scenario) who had a routine health visit but were not yet sampled as being screened at historical rates;
d) Assign empirically informed probabilities of receiving alcohol brief intervention to all individuals who were sampled as having been screened for hazardous alcohol use, based on NSDUH data and conditional on alcohol use category and age, sex, race and ethnicity, and educational attainment;
e) Assign empirically informed probabilities of receiving alcohol brief intervention to additional individuals (N determined by expansion scenario) who were screened for alcohol use but were not yet sampled as receiving a brief intervention at historical rates;
f) Apply reduction in alcohol consumption (g per day) to those that were sampled to receive brief intervention due to *expanded* rates (i.e., expanded screening and/or expanded brief intervention contingent), accounting for each individual's baseline alcohol consumption levels; sample and transition individuals classified as heavy episodic drinkers to non-HED status; and update alcohol use categories given new continuous alcohol consumption levels.

The process for modelling the screening and brief intervention expansion is further detailed in section *1.3.5 Sub-models*.

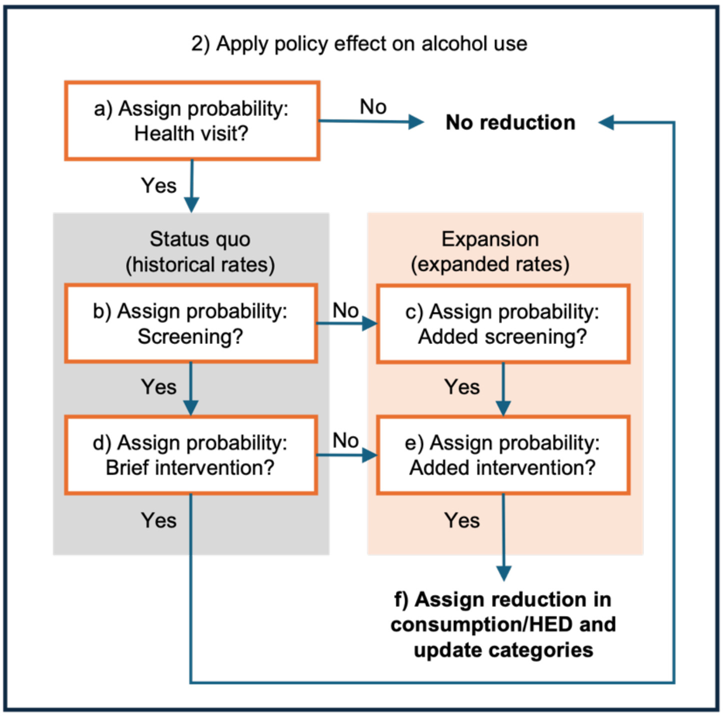

Figure S3. Process for applying changes in alcohol consumption due to screening and brief intervention expansion.

© 2026 Lemp, JM et al. JAMA Health Forum.

1.2 Design concepts

1.2.1 Stochasticity

Pseudo-random number (PRN) sampling is used throughout the simulation to assign simulated individuals with characteristics and behaviour. Stochasticity is introduced by varying the seed for the PRN generator. There are different ways in which stochasticity is introduced in the model.

In a first set of mechanisms, individuals have a probability of changing their characteristics or behaviours. A PRN between 0 and 1 is sampled for each individual and their individual probability (e.g., to die) is compared to this number. This applies to:

- *Remove individuals due to death (not explicitly modelled causes):* Stochasticity is introduced by comparing cumulative probabilities (i.e., mortality rates) of causes of death that are not explicitly being modelled through alcohol-related risk functions to a PRN from a uniform distribution at each time step. The cumulative probability of dying, conditional on each individual's characteristics (i.e., age, sex, race and ethnicity, and educational attainment), was generated using NVSS data (see 1.3.2 Input data). Individuals are assigned as dead if the cumulative probability exceeds the PRN and are consequently removed from the simulation.
- *Remove individuals due to death (explicitly modelled alcohol-related causes):* Stochasticity is introduced by comparing each individual's cumulated absolute risk for specific diseases to a PRN from a uniform distribution at each time step. Each individual's absolute risk for each cause is calculated by multiplying the baseline mortality rate with their individual relative risk. Individuals are assigned as dead if the cumulative probability exceeds the PRN and consequently removed from the simulation. Additional sources of stochasticity in this process are introduced in subsequent paragraph.
- *Updating educational attainment:* Education transitions incorporate stochasticity to determine which individuals younger than 35 transition to higher levels of education. The function uses a PRN drawn at each time step to determine the new education level for each individual. The education level is updated based on where the PRN falls within cumulative transition probability thresholds conditional on age, sex, race and ethnicity based on Panel Study of Income Dynamics (PSID) data (see 1.3.2 Input data).
- *Updating alcohol use categories:* Stochasticity is introduced by the ordinal logistic regression model using pseudo-longitudinal BRFSS data assigning individuals a probability of moving to a different alcohol use category conditional on age, sex, race and ethnicity, and educational attainment. A PRN drawn for each individual at each time step is compared to that probability to decide which categories people may move to.

Stochasticity is also introduced in:

- *Updating alcohol consumption levels:* There is a beta distribution for each subgroup (defined by age, sex, race and ethnicity, educational attainment, and alcohol use category) that is fitted to the BRFSS data (see 1.3.2 Input data). Individuals who change their alcohol use category are re-assigned a new value indicating their consumption level in g per day based on sampling from these beta distributions.
- *Adding and removing individuals due to birth and migration:* There is a migration (18-79 years of age) and birth rate (18-year-olds) for individuals that need to enter the model

© 2026 Lemp, JM et al. JAMA Health Forum.

at each time step that is based on American Community Survey (ACS) data (see 1.3.2 Input data). The number of individuals that need to enter are randomly sampled (by age, sex, race and ethnicity) from each corresponding year of the BRFSS data. There is also an outward migration rate for individuals that need to leave the model in each year by age, sex, race and ethnicity. The number of individuals to leave are randomly sampled from the simulation and removed.

· *Remove individuals due to death (explicitly modelled alcohol-related causes):* There are risk functions required for each explicitly modelled alcohol-related cause (for details, see 1.3.5 Sub-models). The parameters of these risk functions are sampled to generate base rates and the corresponding mortality rates and YLL.

Screening and brief intervention policy:

· *Assigning probabilities to move through the screening and brief intervention cascade:* Each individual's probability to have a health visit, receive alcohol screening, and brief intervention is progressively calculated from the logistic regression model conditional on age, sex, race and ethnicity, education, and alcohol use category using data from NSDUH (see 1.3.2 Input data).

· *Estimation an individual-level brief intervention effect on consumption:* Individuals are randomly assigned a reduction in g per day from the population effect distribution. Each individual's reduction is scaled conditional on their baseline consumption, which is in turn randomly sampled from the population baseline effect distribution (for details, see 1.3.5 Sub-models).

1.2.2 Observation

The observations are separated into (1) within the simulation loop, observed at each time step (i.e., year), and (2) the results outside the simulation loop, summarised post simulation.

For (1): Population data is stored and updated at each time step in the *data* variable. This contains basic general information on each individual; a unique id, age, sex, race and ethnicity, educational attainment, binary drinking status, alcohol consumption level (in g per day), alcohol use category, simulation entry year, and age in categorical form. Alcohol consumption data is stored at each time step and as discrete alcohol use categories and consumption levels of all individuals, alongside sociodemographic data (i.e., age, sex, race and ethnicity, and educational attainment). HED prevalence is also recorded at each time step with the same sociodemographic stratification (i.e., age, sex, race and ethnicity, and educational attainment). Similarly, disease-specific mortality rates and years of life lost (YLL) of the disease shown in table S2, as well as mortality rates and YLL for disease not explicitly modelled are stored at each time step, stratified by the same sociodemographic characteristics (i.e., age, sex, race and ethnicity, and educational attainment).

For (2): Summary data, depending on the output specified, is processed and provided at the end of the simulation. Demographics as an output provides a more in-depth view of the population output, giving year by year information of the simulated population. Alcohol can be specified as the observed output, which provides stratified information of alcohol consumption at each time step. Three different output formats are available, providing alcohol consumption data by stratifying variables, which can include sex, age, educational attainment and race and ethnicity: *alcoholcont* provides mean alcohol consumption levels; *alcoholcat* gives the proportion of individuals within each alcohol use category; and *hed* provides HED prevalence within each demographic group. Likewise, mortality can be the

© 2026 Lemp, JM et al. JAMA Health Forum.

specified output, which provides a yearly summary of stratified mortality rates by sex, age, race and ethnicity, and educational attainment (i.e., deaths for each modelled alcohol-related cause split by deaths through remaining all-cause mortality and alcohol-related deaths in a given subgroup). Alongside mortality rates, the output provides simulated YLL (with 75 years of age as the reference life expectancy) as well as observed mortality rates and YLL.

1.3 Details

1.3.1 Initialisation

Individuals are initialised to form a representative population of the resident US adult population (18 to 79 years of age). Default population size is set to 1,000,000 individuals but can be altered, generally by factors of 10.

There are three stages of model initialisation:

1) Retrieve population constraints: This is the raw data processing of the population data provided by the ACS, and corresponds to the US census in decennial years (2000, 2010). It removes all individuals older than 79 and converts variables for age categories, sex, race and ethnicity, and educational attainment into new categorical values. It then groups the data by these variables and an encoded state identification number to extract the population weights of each sociodemographic group. The script outputs a summary CSV-file, showing the weights of each sociodemographic group at the national level, with both numerical and name identifiers. These weights are used as the population constraints.
2) Generate weights for iterative proportional fitting[11] (IPF): Data from the population constraints generated and the BRFSS to capture alcohol consumption are used. The data is processed to select the required variables in the specified year and ensure the data is ready for IPF. The weights are then generated for the dataset. Within this, it filters the population constraints and the BRFSS data to include only the regional level (US), creates a binary matrix of demographic categories, and aligns the individual data with the population constraints. The IPF algorithm adjusts initial weights to ensure the weighted individual data matches the population constraints. Finally, it converts these weights to integers and returns a data frame with individual IDs, and their corresponding weights.
3) Generate the baseline population: The synthetic baseline population is generated using the weights calculated above. The weights are initially normalised, scaled, and rounded to the nearest integer to match the population size. The synthetic population is created by expanding the population data according to the new weights, and then sampling *n* rows of the expanded population, where *n* is the desired population size, to create a representative synthetic population. To ensure that the data is in the correct format for future use, the data is post processed. It creates a flag for former drinkers, standardising sex coding, selecting and renaming columns for microsimulation compatibility, and adding a spawn year, and age categories. It then saves the processed data to a CSV-file named according to the selected region (US) and population size.

1.3.2 Input data

© 2026 Lemp, JM et al. JAMA Health Forum.

The population size in each cross-tabulated sociodemographic category (e.g., Black, non-Hispanic men aged 18-24 with low education) is determined using data from the ACS and US census, which provide the share of individuals in each category. Individuals with specific attributes, including alcohol consumption, are then sampled based on the BRFSS data to create the synthetic baseline population with the default population size of a million individuals. An overview of all individual-level data sources used to inform the microsimulation is provided in Table S3.

© 2026 Lemp, JM et al. JAMA Health Forum.

Table S3. Overview of individual-level data sources used in the microsimulation.

Data source	Years	Design	Sampling	Purpose
US Census[12]	2000, 2010	Cross-sectional	Full assessment of the US population	To obtain population counts by population subgroup for baseline population and modelling birth and migration, downloaded from IPUMS[3]
American Community Survey (ACS)[3]	Annual, 2000-2021	Cross-sectional	US civilian population, representative on the national and the state level; institutionalised populations and people living in grouped quarters included since 2006	
Panel Study of Income Dynamics (PSID)[13]	Biennial, 1999-2021	Cohort	US civilian, non-institutionalised population, nationally representative	To inform prior beliefs for the educational attainment transition model
Behavioral Risk Factor Surveillance System (BRFSS)[14]	Annual, 2000-2019	Cross-sectional	US civilian, non-institutionalised population, representative on the national and the state level	To derive individual's characteristics, serve as training data for HED models, and inform prior beliefs for alcohol consumption transition model; data corrected for underreporting of alcohol use in survey studies (see [5])
National Epidemiologic Survey on Alcohol and Related Conditions (NESARC)	2001-2002 (NESARC wave I), 2012-2013 (NESARC wave III)	Cross-sectional	US civilian, non-institutionalised population; nationally representative	To obtain distribution of beverage-specific alcohol consumption by population subgroup and alcohol use category
National Survey on Drug Use and Health (NSDUH)[15]	2013-2019	Cross-sectional	US civilian, non-institutionalised population; nationally representative	To inform individual's progress through screening and brief intervention cascade (health visit, screening, brief intervention)
National Vital Statistics System (NVSS)[9]	Annual, 2000-2023	Registry	Full assessment of individual death records	To inform mortality rates

Note: IPUMS: Integrated Public Use Microdata Series. All data sources are restricted to individuals aged 18 years and older.

© 2026 Lemp, JM et al. JAMA Health Forum.

1.3.3 Race and ethnicity coding in individual data sources

Table S4. Overview of race and ethnicity coding in individual data sources used in the microsimulation.

Data source	Assessment of Hispanic ethnicity	Imputation of race and ethnicity in this study	Handling of multiple-race groups
American Community Survey (ACS) and US Census	Separate question on Hispanic origin	N/A	Historically compatible race variable *RACHSING* which classifies into 1. White, 2. Black, 3. American Indian/Alaska Native, 4. Asian or Pacific Islander, 5. Hispanic (based on the question on Hispanic origin). 3-4 are classified as "other". All people who reported Hispanic origins are classified as Hispanic regardless of their race response. Data owners assign non-Hispanic people to a single race/ethnicity group, using methods similar to those described in [16] but with updated source data (1997-2018 NHIS data).
Panel Study of Income Dynamics (PSID)	Separate question on Hispanic origin since 2005; an option to report a race that may be considered 'Hispanic' was included between 1990 and 2003 (e.g., "Spanish-American", "Puerto Rican, Mexican")	A complex imputation method based on hierarchical decision tree (e.g. taking self-report over reported by a spouse) was developed; see Buckley et al. 2025.[4]	'First response' race was considered only, i.e., multiple-race category was not included
National Vital Statistics System (NVSS)	Separate question on Hispanic origin; identical question since 2003, between 2000 and 2002 similar question but with fewer answering options	All deaths with unknown race and/or ethnicity are assigned to "other" race and ethnicity group	Bridged race variable provided (*488 Hispanic Origin/Race Recode*); single race assigned by data owners using regression models and 1997-2000 census data[17]
Behavioral Risk Factor Surveillance System (BRFSS)	Separate question on Hispanic origin	N/A	No reporting of multiple race
National Epidemiologic Survey on Alcohol and Related Conditions (NESARC)	Separate question on Hispanic origin	N/A	Individuals with multiple races were assigned to "other" race group; for NESARC wave III this was done by the data owners

Note: In 2020, the Census Bureau updated the questionnaire text, processing, and coding of the race and Hispanic origin questions, resulting in notable changes to the distribution of race and Hispanic origin categories. We therefore we unable to use the variable RACE.

© 2026 Lemp, JM et al. JAMA Health Forum.

1.3.4 Modelling forward in time

The synthetic population of the microsimulation relies on four main types of demographic input data that must be projected forward in time:

1) *Population counts* that serve as demographic subgroup constraints to align the synthetic population with the US adult population
2) *“Births” or 18-year-olds* that enter the synthetic population each year
3) *Death counts* tabulated by demographic subgroup and cause of death to determine how many individuals need to be removed from the synthetic population each year
4) *Inward migrants* that enter the synthetic population each year

Future synthetic population constraints relevant to items 1-3 are informed by population projections by the US Census Bureau that uses estimates of the resident population on July 1, 2022, as the base for modelling the US population and all-cause mortality forward.[18] These estimates are based on the cohort-component method, a standard demographic approach that projects the base resident population by estimating the three components of population change: births, deaths, and net international migration.[18] In brief, each year, existing cohorts are aged using age-specific survival probabilities (mortality rates) and net migration. New birth cohorts are generated by applying age-specific fertility rates to the existing female population, adjusted for infant mortality and migration. Note that while CDC’s base population on July 1, 2022, incorporates observed increased mortality in 2020 from the COVID-19 pandemic, this was assumed to be an isolated event. Therefore, 2020 mortality data were not included in formulation of the mortality assumptions and long-term fertility, mortality, and migration in subsequent years were modelled as if demographic trends return to pre-pandemic trajectories.

Different methods were used to adapt and integrate future projections, ensuring a smooth transition between observed data and projected values, and to characterize individuals entering the synthetic population (Table S5).

For 1) and 2), US Census population projections did not align perfectly with historical population constraints derived from ACS data (2000-2021). To bridge this, we calculated year- and group-specific growth rates (by sex, age, and race and ethnicity) from the Census projections and applied them to ACS counts starting in 2021.

For 2), we constructed a projected population pool wherein education, former drinking status, and average daily alcohol consumption were drawn from 2017-2019 BRFSS distributions. Given some modest upward trends in current drinking status among non-Hispanic White women and Hispanic women, and downward trends in Non-Hispanic Black and Hispanic men observed from 2010-2019, we projected these trends forward using a regression model to reflect likely changes in drinking prevalence among new 18-year-olds.

For 3), while the US Census Bureau provides projected all-cause mortality by sex and age, it does not break these down by cause, race and ethnicity or education. To address this, we applied Joint Dirichlet regression models, to compute fractions of relevant cause-specific mortality within the mortality envelope. Figure S4 showcases projections for selected causes of death.

© 2026 Lemp, JM et al. JAMA Health Forum.

For 4), US Census projections do not provide disaggregated data on inward migration. Therefore, we estimated future migration using a linear regression model that predicted the log rate of inward migration based on 2010-2019 ACS data.

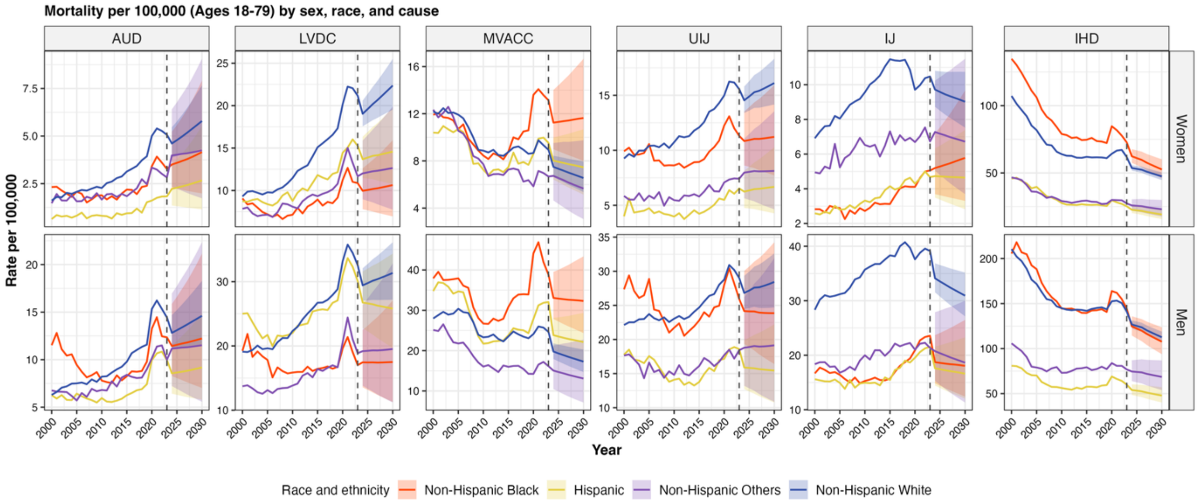

Figure S4. AUD = Alcohol use disorder. LVDC = Liver disease and cirrhosis. MVACC = Injuries from motor vehicle-related accidents. UIJ = Unintentional injuries. IJ = Intentional injuries (suicide). IHD = Ischaemic heart disease. Projected mortality rate per 100,000 by sex and race for selected alcohol-related causes of death. Projections and 95% confidence intervals are derived from sex-specific Joint Dirichlet regression models using NVSS data to train the model and CDC Census Bureau’s death projections as the mortality envelope.

© 2026 Lemp, JM et al. JAMA Health Forum.

Table S5. Overview of data that has been projected forward.

Output	Years	Data source	Variables/Stratification	Method
1) Population counts by sex, age, and race and ethnicity	2022-2030	US Census - Projected Population by Single Year of Age, Sex, Race, and Hispanic Origin for the United States: 2022 to 2100: Main Series[19]	Sex, age category, race and ethnicity	Calculation of year-specific and group-specific growth rates based on the Census projections that were then applied to ACS population counts starting in 2021
2) Birth count (18-year-olds) by sex and race and ethnicity	2022-2030	US Census - National Population by Characteristics, 2021 (July, 1), Residential Population by Age, Sex, Race and Hispanic[20]	Sex, race and ethnicity	
2) Characteristics of new 18-year-olds	2023-2030	BRFSS, 2010-2019[14]	Education, current and former drinking status, average g per day	Regression models predicting individual's characteristics based on historical data
3) Death counts, by cause and sex, age, education, and race/ethnicity	2024-2030	National Vital Statistics System (NVSS), 2010-2019, 2023[9]<br><br>US Census' total death projections, 2023-2030, by sex and age[19]	Sex, education, race and ethnicity, cause of death	Joint Dirichlet models, estimated separately for women and men, predict the education-, race- and cause-specific response matrix of fractions that sum up to 1 for each age group per year based on historical NVSS death data.<br><br>These fractions are then multiplied by the total death counts (by sex and age) as projected by the US Census Bureau.
4) Inward migration rates by sex, age, and race and ethnicity	2022-2030	American Community Survey (ACS), 2010-2019[3]	Sex, age category, race and ethnicity	Linear regression model predicting the log rate of historical migration rates from year, sex, age category, race and ethnicity

© 2026 Lemp, JM et al. JAMA Health Forum.

1.3.5 Sub-models

HED

The microsimulation uses annual BRFSS survey data from 2011 to 2022 to train machine learning models to estimate the probability of an individual engaging in HED as a function of average daily alcohol consumption and demographic characteristics. These probabilities are generated using three XGBoost-based classification models: one trained on men aged <35 years, one trained on men aged ≥35 years, and one trained on women and men aged ≥35 years. Predictions from the first two models are applied to their respective male subgroups, whereas predictions from the third model are applied only to women.

Training data was restricted to current drinkers with average alcohol consumption between 1 and <60 g/day, as modelled probabilities are generated for and assigned only to this population while non-drinkers, former drinkers, and current drinkers consuming <1 g/day are manually classified as non-HED and current drinkers consuming ≥60 g/day are manually classified as HED. Model predictors include average alcohol consumption (g/day) and age as continuous variables, as well as race and ethnicity (Non-Hispanic White, Non-Hispanic Black, Hispanic, Others) and educational attainment (high school degree or less, some college, college degree or more). Sex is included as a categorical predictor in only the third model, as the first two models are trained only on men.

Hyperparameters for each XGBoost model were optimized and set to nrounds = 600 (number of iterations), eta = 0.3 (learning rate), max_depth = 6 (maximum depth of decision trees), min_child_weight = 1 (minimum sum of instance weight needed in a child node), and gamma = 0 (minimum loss reduction needed to make a further partition on a leaf node of the tree).[21] To convert probabilities into HED status classifications, a threshold of 0.5 is used where individuals with a probability ≥0.5 are classified as HED and those with a probability <0.5 are classified as non-HED.

Mortality

The microsimulation distinguishes between (1) “all-cause mortality”, i.e., causes that are not being modelled in relation to alcohol use, and (2) causes that are being modelled in relation to alcohol use (Table S2).

For (1), mortality is modelled using death counts in each population subgroup, taken directly from the observed or projected NVSS data and scaled to the population size that is being modelled, calculating a corresponding mortality rate. Here, mortality is sampled using a cumulative probability vector that simultaneously accounts for all-cause mortality while setting aside mortality modelled in relation to alcohol use (see 2). This is to give each person a simultaneous chance to die from each cause that is proportional to the observed mortality rates in the population. At this stage, only individuals who die from “all-cause mortality” are removed from the simulation.

For (2), risk functions linking alcohol use to cause-specific mortality risks, that were informed by published meta-analyses and secondary data analyses, are used to calculate relative risk of mortality from each cause for each individual conditional on their alcohol use. A summary of the methods and sources for each cause of death used can be found in Table S6. Note that where applicable SES-specific risk functions were specified as an alternative to the main

© 2026 Lemp, JM et al. JAMA Health Forum.

risk functions; however, in this study we present results using the default setting ('main version') only.

Table S6. Risk functions implemented in the main version.

Main version					SES-specific version available[a]
**Outcome**	**Method**	**Publication**	**Sex-specific**	**US-specific**	
Liver cirrhosis	Systematic review	Llamosas-Falcón et al. 2022[22]; Llamosas-Falcón et al. 2024[23]	Yes	No[b]	Yes
Hepatitis C liver cirrhosis	Systematic review	Llamosas-Falcón et al. 2024[23]	Yes	No	No
Alcohol use disorder	Systematic review	Carr et al. 2024[24]	Partially	No	Yes
Suicide	Systematic review	Lange et al. 2024[25]	Yes	No	No
Diabetes	Systematic review	Llamosas-Falcón et al. 2023[26]	Yes	No[b]	No
Ischemic heart disease	Systematic review	Zhao et al. 2017[27]	Yes	No	Yes
Ischemic stroke	Systematic review	Larsson et al. 2016[28]	No	No	No
Hypertensive heart disease	Systematic review	Liu et al. 2020[29]	Yes	No	No
Motor vehicle injuries	Secondary data analysis	WHO Global status report 2018[30]	No	No	No
Other unintentional injuries	Secondary data analysis	WHO Global status report 2018[30]	No	No	No

Note: NHIS = National Health Interview Survey. SES = Socio-economic status. [a] Results for SES-specific risk functions were obtained from secondary data analyses of mortality-linked NHIS data. [b] US-specific version is available for potential sensitivity analysis.

For our main version, the team conducted systematic reviews and meta-analyses of the international literature for the following outcomes: liver cirrhosis, AUD, suicide, and type 2 diabetes, following the approach proposed by Jiang et al.[31] The number of papers identified for the liver cirrhosis and diabetes systematic reviews allowed us to obtain US-specific results for these two outcomes, which can be used for sensitivity-analyses. However, in the case of AUD and suicide, there was not enough data to generate US-specific estimates. In addition, for liver cirrhosis, we found evidence that the risk varied by etiology, with a higher overall risk observed for liver cirrhosis ("main pathway") compared to hepatitis C-related liver cirrhosis.[23] This difference is consistent with evidence indicating that alcohol acts as a primary causal agent in non-viral liver cirrhosis, whereas in HCV-related cirrhosis alcohol primarily modifies disease progression.[32] To account for this variation, we implemented a specific risk function for hepatitis C-related liver cirrhosis, ensuring that the model more accurately reflects the differential risk profiles associated with distinct etiology. For type 2 diabetes, we cap the relative risk at 30 and alcohol intake at 100 g/day, consistent with the systematic review, as no data points very identify beyond these levels. Moreover, empirical evidence indicates that the risk is unlikely to exceed this relative risk limit even at very high levels of alcohol use. For cardiac outcomes (ischemic heart disease, ischemic stroke, and

© 2026 Lemp, JM et al. JAMA Health Forum.

hypertensive heart disease) we selected the most recent and comprehensive systematic reviews available on the topic. For motor vehicle injuries and other unintentional injuries, the risk function was extracted from the WHO Global Status Report 2018.[30] For these injury outcomes, distinct risk functions were applied to individuals who engage in heavy episodic drinking and those who do not. Individuals drinking more than 60 grams per day were also assigned the heavy episodic drinking risk function.

For the SES-specific version, we conducted secondary data analyses using the 1997-2018 NHIS data linked to mortality in the 2019 National Death Index. In brief, we found additive interactions of low educational attainment (high school degree or less) with Category III alcohol use (>40 g/day for females and >60 g/day for males) for 100% alcohol-attributable mortality (AAM) and liver diseases and cirrhosis (LVDC) mortality.[33] These additive interactions were supported by the relative excess risk due to interactions (RERI) from Cox proportional hazards models as well as the interaction terms from Aalen's additive hazards models with family income as the alternative indicator for SES. For ischemic heart disease (IHD) mortality, only multiplicative interactions were found, with the protective associations of drinking <20 g/day with IHD mortality being stronger in high SES groups compared with low SES groups, among both sexes.[34] As such, we derived education-specific risk functions for these outcomes to be used as sensitivity analysis. The method used to derive the SES-specific risk function is as follows: we assumed that the sex-specific risk functions from the meta-analysis applied to men and women with middle educational attainment. We also assumed that the ratios of derivatives (or slopes) of risk functions (curves) for low vs. middle, and for high vs. middle education groups are constant and equal to the ratios of slopes obtained from secondary NHIS analysis with interaction of alcohol use and education on liver disease and cirrhosis, AUD and ischemic heart disease.

After calculating relative risk using the risk functions and each individual's level of alcohol use, the relative risks are converted into absolute probabilities of mortality. Specifically, each individual's absolute risk for a particular cause of death is obtained by scaling their relative risk with a base rate, which represents the probability of death in a population without additional risk due to alcohol use:

$$absolute\ risk_{i,t} = relative\ risk_{i,t} \times base\ rate_{j,t}\ ,\ i\ \in\ j\ ,$$

where base rate $base\ rate_{j,t}$ is defined as:

$$base\ rate_{j,t} = \frac{observed\ mortality_{j,t}}{\sum_{i \in j} relative\ risk_{i,t}},$$

with i denoting individuals, j the population subgroups and t the year.

For AUD, all observed deaths are, by definition, attributable to alcohol use. This implies that lifetime abstainers have zero risk of dying from AUD, reflecting 100% alcohol-attributable mortality. To model this, we calculate the base mortality rate for AUD among only the drinking population (i.e., both current and former drinkers) in each subgroup:

$$base\ rate_{j,t}(AUD) = \frac{observed\ mortality_{j,t}(AUD)}{\sum\ relative\ risk_{i,t}},\ i\ \in\ drinking\ population\ of\ j$$

This base rate for AUD is applied only to non-abstainers, while lifetime abstainers are assigned an absolute risk of 0 directly.

The absolute risks of mortality across all modelled causes are then stacked to yield the cumulative probability, which is used to sample cause-specific mortality. Because certain

© 2026 Lemp, JM et al. JAMA Health Forum.

causes of death are very rare in some population subgroups, we apply an inflation adjustment factor to the observed mortality of those subgroups (defined by age categories). This adjustment factor is moderated to ensure that cumulative risks do not exceed 1. After simulating mortality, this inflation factor is deflated back when reporting simulated mortality outputs. The overall process enables the reproduction of the observed mortality rates, as shown in Figure S5.

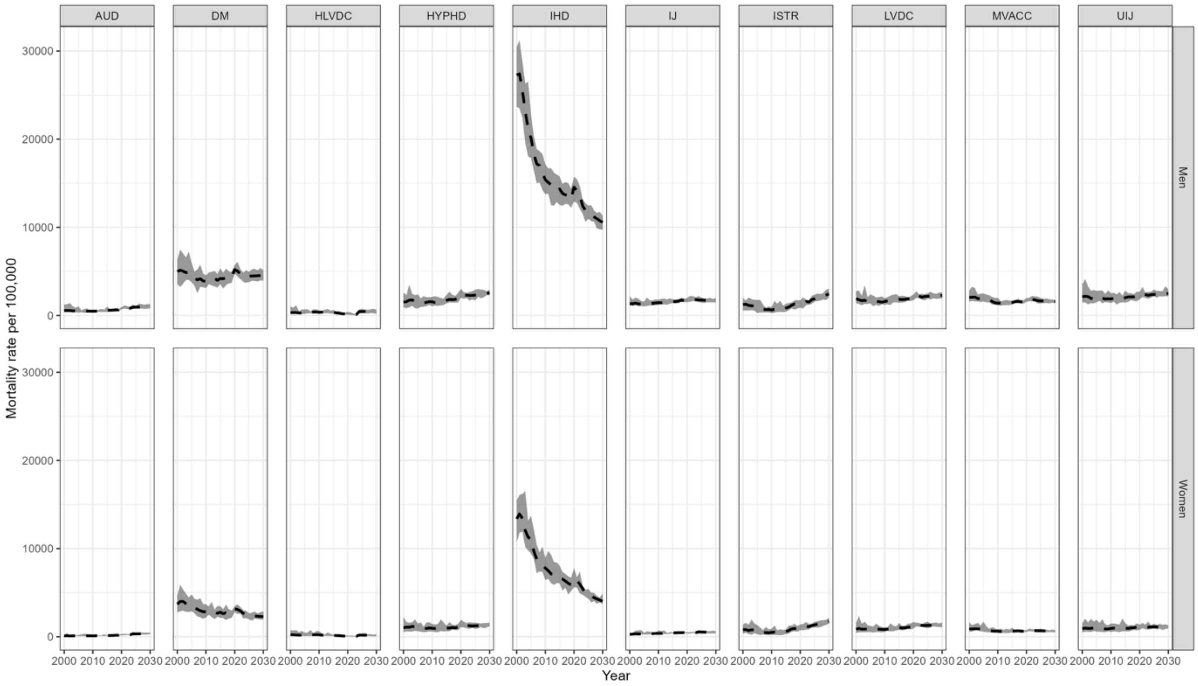

Figure S5. Observed mortality rates per 100,000 for all ten modelled causes of death by sex (dashed black line; Table S2) compared with simulated rates across 20 seed runs (grey shaded area).

Updating educational attainment

The microsimulation used data from the US Census to form the baseline estimates and data from PSID to update individual's educational attainment categories over time. In the US Census data, educational attainment is reported in terms of grades of school completed in the following categories: (1) Less than 9th grade, (2) 9th to 12th grade, no diploma, (3) High school graduate, (4) Some college, no degree, (5) Associate degree, (6) Bachelor's degree and (7) Graduate or professional degree. This data was used to inform the education distribution in the microsimulation at baseline, where category (3) and below represents "High school degree or less", categories (4) and (5) will be classified as "Some college' and categories (6) and above will be classified as "College degree or more". In the American Community Survey data, educational attainment is reported in terms of years of education completed, and ranges from no schooling, through to grade 4-12 and then 1-5+ years of college. We categorized ACS data into the following categories: Grade 12 and below will be classified as "High school degree or less", 1-3 years of college will be classified as "Some college" and 4 and 5+ years of college will be categorized as "College degree or more". In the PSID data, educational attainment is also classified in terms of years of education completed and possible values range from 1-17 years. We classified 12 years or less as "High school degree or less" and 13-15 years as "Some college" (representing, 1, 2 and 3

© 2026 Lemp, JM et al. JAMA Health Forum.

years of college, respectively). Education of 16 years or over was considered as “College degree or more”.

Pre-COVID-19 model

Education transitions are modelled using multi-state Markov models with a homogeneous, continuous-time process[11] and 2005-2019 longitudinal PSID data, restricting changes in educational attainment to individuals aged 18 to 34 years.[4] Educational attainment is therefore assumed to be fixed after age 34, as post-graduate degrees are assumed to have little impact on alcohol use.

The model estimates transitions between 5 states: (1) High school degree or less, (2) Some college (1 year), (3) Some college (2 years), (4) Some college (3 years), (5) College degree or more. The model is estimated conditional on age category (18, 19, 20, 21 as individual years of age, 22-24, 25-29, 30+ as age categories), sex, and race and ethnicity. Allowed instantaneous transitions are restricted to sequential forward transitions to the next higher level of education (see *Q* matrix below). We estimate three separate years in the “some college” category as tunnel states, which prevents individuals from achieving higher educational attainment categories too early (e.g., this would prevent individuals from reaching the “College degree or more” category in 1 year). There is no time component to the model, i.e., education transition probabilities are the same throughout the simulation 2000 to 2019.

To calculate the transition probabilities for a specific sociodemographic subgroup, we start with the baseline transition intensities (Table S7) and adjust these values using Hazard Ratios (HRs) for the relevant sociodemographic properties of that subgroup (Table S8). The allowed transitions are defined in the *Q* matrix:

$$\begin{pmatrix} -q_{12} & q_{12} & 0 & 0 & 0 \\ 0 & -q_{23} & q_{23} & 0 & 0 \\ 0 & 0 & -q_{34} & q_{34} & 0 \\ 0 & 0 & 0 & -q_{45} & q_{45} \\ 0 & 0 & 0 & 0 & 0 \end{pmatrix}$$

The baseline transition intensity matrix (*Q*) defines the instantaneous rates of transitioning between states under baseline conditions (Table S7)—i.e., the values of the covariates are at the reference values. Each element q_{ij} represents the baseline intensity of transitioning from state *i* to state *j* and the diagonal elements q_{ii} are calculated to ensure that the rows sum to zero.

$$q_{ii} = -\sum_{j \neq i} q_{ij}$$

© 2026 Lemp, JM et al. JAMA Health Forum.

Table S7. Baseline (top) and calibrated baseline (bottom) transition intensities (before adjusting for covariates).

State T	State T+1<br>High school degree or less	Some college (1 year)	Some college (2 years)	Some college (3 years)	College degree or more
			Intensity (95% CI)		
High school degree or less	·	0.29 (0.28,0.29)<br>0.29 (0.29,0.29)	·	·	·
Some college (1 year)	·	·	1.01 (0.99,1.01)<br>1.18 (1.17,1.19)	·	·
Some college (2 years)	·	·	·	0.51 (0.50,0.51)<br>0.61 (0.60,0.62)	·
Some college (3 years)	·	·	·	·	1.10 (1.08,1.11)<br>1.45 (1.43,1.46)
College degree or more	·	·	·	·	·

Using the values from Table S7, the baseline *Q* matrix is:

$$\begin{pmatrix} -0.29 & 0.29 & 0 & 0 & 0 \\ 0 & -1.01 & 1.01 & 0 & 0 \\ 0 & 0 & -0.51 & 0.51 & 0 \\ 0 & 0 & 0 & -1.10 & 1.10 \\ 0 & 0 & 0 & 0 & 0 \end{pmatrix}$$

The baseline intensities are adjusted for subgroup specific characteristics using the HRs from Table S5. For a given transition *i j*, the adjusted transition intensity is calculated as:

$$\lambda_{ij} = q_{ij} \cdot HR_{ij}$$

For example, for a white, 19-year-old men transitioning between states ≤High school (high school degree or less) SomeC1 (some college, 1 year):

$$\lambda_{\leq High\,school \to SomeC1} = (-0.29) \times 0.62 = -0.1798$$

The adjusted Q_{group} matrix for a 19-year-old White man is therefore:

$$Q_{group} = \begin{pmatrix} -0.1798 & 0.1798 & 0 & 0 & 0 \\ 0 & -0.7878 & 0.7878 & 0 & 0 \\ 0 & 0 & -0.6477 & 0.6477 & 0 \\ 0 & 0 & 0 & -0.8140 & 0.8140 \\ 0 & 0 & 0 & 0 & 0 \end{pmatrix}$$

© 2026 Lemp, JM et al. JAMA Health Forum.

The transition probability matrix *P(t)* is calculated using the matrix exponential of the adjusted intensity matrix:

$$P(t) = \exp(Q_{group} \cdot t)$$

This must be calculated using statistical software such as R; the full transition probabilities used in our model are available online: https://figshare.com/s/1355ee5cf3c8bd81b0dd.

© 2026 Lemp, JM et al. JAMA Health Forum.

Table S8. Hazard ratios (HR) for each sociodemographic factors' influence on the transition intensities, calibrated model version.

		Original model (estimated using PSID data)				Calibrated model (calibrated to ACS 2000-2010, validated to ACS 2011-2019)			
		≤High school → SomeC1	SomeC1 → SomeC2	SomeC2 → SomeC3	SomeC3 → ≥College	≤High school → SomeC1	SomeC1 → SomeC2	SomeC2 → SomeC3	SomeC3 → ≥College
Age	18	1	1	1	1	1	1	1	1
	19	0.62 (0.61,0.62)	0.78 (0.77,0.79)	1.27 (1.25,1.28)	0.74 (0.73,0.75)	0.60 (0.59,0.61)	0.71 (0.70,0.71)	1.09 (1.08,1.11)	0.67 (0.66,0.69)
	20	0.23 (0.23,0.23)	0.50 (0.50,0.51)	1.01 (1.00,1.02)	0.73 (0.72,0.75)	0.24 (0.23,0.24)	0.44 (0.44,0.45)	0.86 (0.84,0.87)	0.55 (0.55,0.56)
	21	0.16 (0.16,0.17)	0.32 (0.31,0.32)	0.65 (0.64,0.66)	0.59 (0.58,0.6)	0.15 (0.15,0.15)	0.33 (0.32,0.33)	0.56 (0.56,0.57)	0.42 (0.41,0.42)
	22-24	0.12 (0.12,0.12)	0.19 (0.19,0.20)	0.31 (0.31,0.31)	0.39 (0.38,0.39)	0.13 (0.13,0.13)	0.18 (0.17,0.18)	0.33 (0.32,0.33)	0.30 (0.29,0.30)
	25-29	0.11 (0.11,0.12)	0.18 (0.18,0.18)	0.19 (0.19,0.19)	0.20 (0.20,0.20)	0.12 (0.12,0.12)	0.17 (0.17,0.17)	0.17 (0.17,0.18)	0.17 (0.16,0.17)
	30-34	0.10 (0.10,0.11)	0.14 (0.14,0.14)	0.15 (0.15,0.15)	0.22 (0.21,0.22)	0.12 (0.11,0.12)	0.12 (0.12,0.13)	0.14 (0.14,0.15)	0.16 (0.16,0.16)
Sex	Men	1	1	1	1	1	1	1	1
	Women	1.34 (1.33,1.34)	1.18 (1.18,1.19)	1.07 (1.07,1.08)	1.14 (1.13,1.14)	1.39 (1.38,1.40)	1.07 (1.06,1.08)	1.00 (0.99,1.01)	1.06 (1.05,1.07)
Race and ethnicity	Non-Hispanic White	1	1	1	1	1	1	1	1
	Non-Hispanic Black	0.71 (0.70,0.72)	0.73 (0.73,0.74)	0.85 (0.84,0.86)	0.58 (0.57,0.58)	0.77 (0.76,0.78)	0.74 (0.73,0.75)	0.97 (0.96,0.99)	0.61 (0.60,0.62)
	Hispanic	0.89 (0.88,0.90)	0.76 (0.75,0.77)	0.89 (0.88,0.90)	0.54 (0.54,0.55)	0.82 (0.81,0.83)	0.85 (0.84,0.87)	0.74 (0.73,0.75)	0.57 (0.56,0.59)
	Other	1.19 (1.18,1.21)	1.04 (1.03,1.05)	1.16 (1.15,1.18)	0.82 (0.81,0.84)	1.29 (1.27,1.31)	1.16 (1.13,1.18)	1.18 (1.16,1.21)	0.91 (0.90,0.92)

Notes: All values are Hazard Ratios (HRs), 95% confidence intervals in brackets. ACS: American Community Survey, ≤High school: High school degree or less, SomeC1: some college 1 year, SomeC2: some college 2 years, SomeC3: some college 3 years, ≥College: College degree or more.

© 2026 Lemp, JM et al. JAMA Health Forum.

COVID-19 model

Education transitions during the COVID period are modelled using 2019-2021 longitudinal PSID data, applying the same multi-state Markov model framework as for the pre-COVID analysis. The model estimates transitions across five educational states: (1) High school degree or less, (2) Some college (1 year), (3) Some college (2 years), (4) Some college (3 years), (5) College degree or more. The model is estimated conditional on age category (18, 19, 20 as individual years of age, 21-25 and 26+ as age categories), sex, and race and ethnicity. Baseline transition intensities and hazard ratios for each sociodemographic factor during the COVID period are reported in Tables S9 and S10.

Table S9. Baseline transition intensities (before adjusting for covariates).

State T	State T+1<br>High school degree or less	Some college (1 year)	Some college (2 years)	Some college (3 years)	College degree or more
			Intensity (95% CI)		
High school degree or less	·	0.20<br>(0.20,0.20)	·	·	·
Some college (1 year)	·	·	1.23<br>(1.20,1.26)	·	·
Some college (2 years)	·	·	·	0.41<br>(0.40,0.43)	·
Some college (3 years)	·	·	·	·	0.22<br>(0.20,0.23))
College degree or more	·	·	·	·	·

Table S10. Hazard ratios (HR) for each sociodemographic factors' influence on the transition intensities.

		Original model (estimated using 2019-2021 PSID data)			
		≤High school → SomeC1	SomeC1 → SomeC2	SomeC2 → SomeC3	SomeC3 → ≥College
Age	18	1	1	1	1
	19	0.65<br>(0.64,0.67)	0.51<br>(0.50,0.53)	1.24<br>(1.20,1.29)	1.18<br>(1.09,1.29)
	20	0.23<br>(0.22,0.24)	0.32<br>(0.31,0.33)	1.40<br>(1.35,1.45)	4.41<br>(4.09,4.77)
	21-25	0.11<br>(0.10,0.11)	0.09<br>(0.09,0.09)	0.39<br>(0.38,0.41)	1.54<br>(1.43,1.65)
	26-34	0.05<br>(0.05,0.06)	0.07<br>(0.06,0.07)	0.11<br>(0.11,0.12)	0.40<br>(0.37,0.43)
Sex	Men	1	1	1	1
	Women	1.65<br>(1.61,1.68)	0.94<br>(0.92,0.96)	1.66<br>(1.63,1.69)	1.32<br>(1.29,1.35)
Race and ethnicity	Non-Hispanic White	1	1	1	1
	Non-Hispanic Black	0.63<br>(0.61,0.64)	0.63<br>(0.61,0.65)	0.50<br>(0.49,0.52)	0.79<br>(0.76,0.82)
	Other	0.94<br>(0.90,0.98)	1.28<br>(1.23,1.34)	1.54<br>(1.49,1.60)	1.04<br>(1.00,1.08)

© 2026 Lemp, JM et al. JAMA Health Forum.

Hispanic	0.83 (0.80,0.85)	1.30 (1.27,1.34)	0.69 (0.67,0.71)	0.87 (0.84,0.90)

Note: All values are Hazard Ratios (HRs), 95% confidence intervals in brackets. ACS: American Community Survey, ≤High school: High school degree or less, SomeC1: some college 1 year, SomeC2: some college 2 years, SomeC3: some college 3 years, ≥College: College degree or more.

Calibration and validation

Education transition probabilities for the pre-COVID-19 period are calibrated to match the observed proportions of individuals in each educational attainment category by age, sex, and race and ethnicity over time according to the observed target data using 2000-2010 and validated using reserved data from 2011-2019 ACS.[35] The education transition probabilities were a good fit to the data and the calibration process converged after 4 waves of calibration with a mean implausibility metric of 2.51 across all retained parameter sets. All calibrated parameter sets passed the validation criteria (mean implausibility: 2.94) for the validation period 2011-2019.

For each wave, the implausibility metric for each candidate parameter set i, aggregated over all simulator outputs j, is calculated as:

$$I_i = \frac{1}{J}\sum_{j=1}^{J} \frac{|\hat{z}_{i,j} - x_j|}{\sqrt{V_{s,j} + V_{o,j} + V_{m,j}}}, \quad (1)$$

where $\hat{z}_{i,j}$ denote the jth simulated mean output and x_j represents the corresponding observed target data. The denominator incorporates three sources of uncertainty: observation uncertainty $V_{o,j}$, represented by the measurement error of the observed targets; ensemble variability $V_{s,j}$, quantified as the 90th percentile of the sample simulated variance; and model discrepancy $V_{m,j}$, measured as 10% of the variance of the simulator outputs for the present wave.[36] We retain all candidate parameter sets with a mean implausibility of three or less. The reported mean implausible value is the mean of the implausibility metric across all retained parameter sets.

Education transition probabilities for the COVID-19 period are not calibrated due to a lack of available data.

Updating alcohol consumption

Transitions between alcohol use categories (i.e., non-drinker, category 1 [low risk, up to 20 g per day for women and up to 40 g per day for men], category 2 [medium risk, more than 20 g per day up to 40 g per day for women and more than 40 g per day up to 60 g per day for men], category 3 [high risk, more than 40 g per day for women and more than 60 g per day for men]) conditional on previous alcohol use category, age category (i.e., 18-24, 25-64, 65+), sex, race and ethnicity, and educational attainment were obtained from ordinal logistic regression. The regression model was fitted to pseudo-longitudinal data that had been generated using the simulation and the 2000-2010 BRFSS data. This approach was chosen due to difficulties fitting the simulation to observed data (BRFSS 2015-2019) using continuous-time Markov models, as proposed in the study protocol.[1,7]

The pseudo-longitudinal population was generated by deterministically assigning daily alcohol consumption levels (in g per day) to each synthetic individual using a rank-matching function

© 2026 Lemp, JM et al. JAMA Health Forum.

based on the BRFSS data. The function ranks synthetic individuals (within age, sex, race and ethnicity, and education categories) and matches them with a BRFSS individual by rank, assigning alcohol values accordingly. This process resulted in a pseudo-longitudinal population that mirrors BRFSS alcohol consumption data for the synthetic population, providing data for ordinal logistic regression model estimation (see Figure S6).

An ordinal logistic regression model was fitted to this pseudo-longitudinal BRFSS data using alcohol use category in year *t* as outcome variable and age, sex, race and ethnicity, and educational attainment in year *t* – 1 as predictors. Interaction effects between educational attainment X alcohol use category were included.

The fitted ordinal logistic regression is used to calculate a probability of each individual belonging to each alcohol use category, conditional on age, sex, race and ethnicity, and educational attainment. A PRN is sampled for each individual and compared to these probabilities to assign each individual a new alcohol use category in each year of the simulation. In the ordinal logistic regression model, the non-drinker category includes both former drinkers and lifetime abstainers. Each year within the simulation, a random subset of non-drinkers is selected and reclassified as former drinkers based on the proportion of former drinkers observed in the BRFSS data. The remaining non-drinkers are classified as lifetime abstainers.

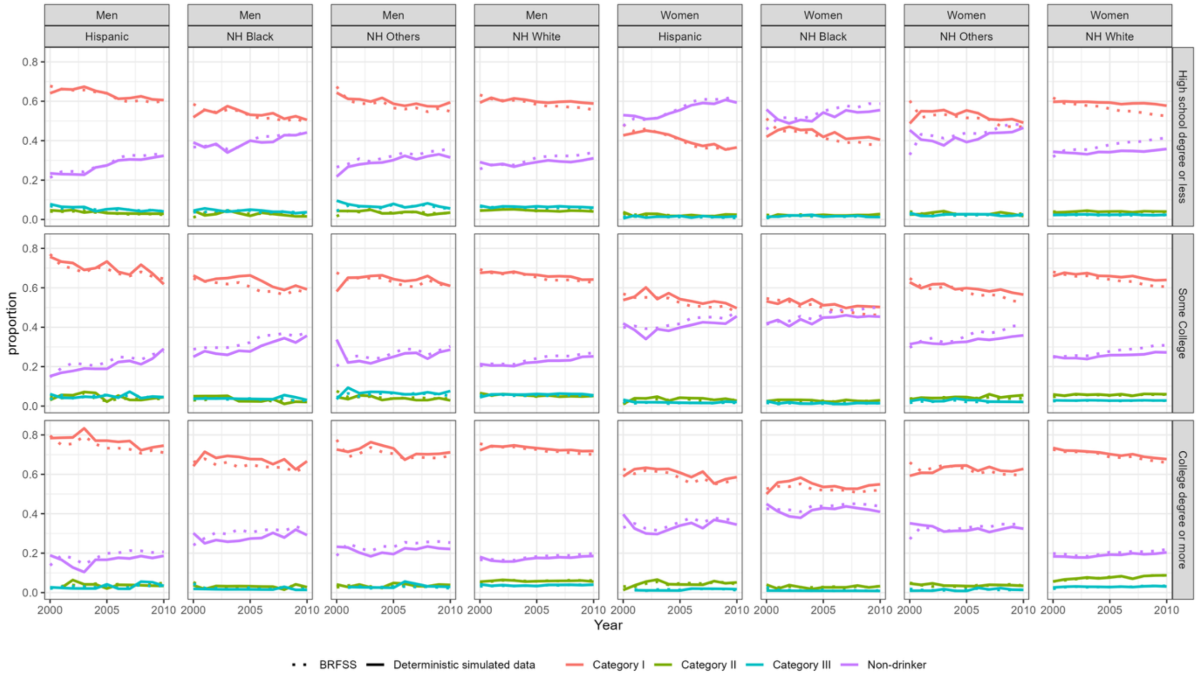

Figure S6. Proportion of individuals in each alcohol use category by sex, race and ethnicity and educational attainment in Behavioural Risk Factor Surveillance System (BRFSS) data compared to pseudo-longitudinal dataset used to fit ordinal logistic regression models. NH = non-Hispanic.

Calibration and validation

© 2026 Lemp, JM et al. JAMA Health Forum.

The ordinal logistic regression model was calibrated to observed target data from the BRFSS (2011-2015). The calibration process converged after 4 waves of calibration with a mean implausibility metric of 3.27 across all samples. All calibrated samples had a mean implausibility value of 3.34 over the validation period 2016-2019. Table S11 depicts the full specification of the ordinal logistic regression model before and after calibration.

© 2026 Lemp, JM et al. JAMA Health Forum.

Table S11. Original and calibrated Ordinal Logistic Regression model for modelling alcohol use transitions.

	Original model (estimated using pseudo-longitudinal BRFSS data)			Calibrated model (calibrated to BRFSS 2011-2015, validated to 2016-2019)		
	OR	Lower	Upper	OR	Lower	Upper
Alcohol use category 1[a]	0.00	0.00	0.00	0.00	0.00	0.00
Alcohol use category 2[a]	0.00	0.00	0.00	0.00	0.00	0.00
Alcohol use Non-drinker[a]	0.00	0.00	0.00	0.00	0.00	0.00
Women[b]	0.87	0.84	0.91	0.87	0.87	0.87
Age 25-64[c]	0.58	0.54	0.62	0.58	0.58	0.58
Age 65+[c]	0.37	0.35	0.41	0.38	0.38	0.38
≤High school[d]	0.83	0.60	1.16	0.95	0.95	0.95
Some college[d]	0.64	0.46	0.90	0.63	0.63	0.63
Non-Hispanic Black[e]	0.64	0.·60	0.68	0.64	0.64	0.64
Hispanic[e]	0.71	0.60	0.77	0.71	0.71	0.71
Non-Hispanic Others[e]	0.80	0.74	0.86	0.80	0.80	0.80
Category 1[a] * ≤High school	1.06	0.75	1.48	1.08	1.08	1.08
Category 1[a] * Some college	1.35	0.95	1.90	1.37	1.37	1.37
Category 2[a] * ≤High school	0.99	0.67	1.45	1.06	1.06	1.06
Category 2[a] * Some college	1.37	0.93	2.03	1.34	1.34	1.34
Non-drinker * ≤High school	0.54	0.39	0.76	0.55	0.55	0.55
Non-drinker * Some college	1.15	0.81	1.63	1.14	1.14	1.14
Non-drinker | Category 1	0.00	0.00	0.00	0.00	0.00	0.00
Category 1 | Category 2	0.00	0.00	0.00	0.00	0.00	0.00
Category 2 | Category 3	0.03	0.02	0.04	0.03	0.03	0.03

Note: BRFSS: Behavioral Risk Factor Surveillance System, ≤High school: High school degree or less, ≥College: College degree or more. All dynamic variables (i.e., age, educational attainment, alcohol use category) use previous year's values (*t-1*) to predict the current year (*t*). [a] ref = category 3, [b] ref=age 18-24, [c] ref = Men, [d] ref = ≥College, [e] ref = non-Hispanic White

A separate process is used to assign individuals a continuous g per day value from within their alcohol use category. Individuals are only assigned a new continuous g per day value when they change their alcohol use category. This is controlled by beta distributions fitted to the 2000-2014 BRFSS data for each alcohol use category, age (18-24, 25-64, 65+), sex, race and ethnicity, and educational attainment category. For the 18-24 age group, individuals in the college and some college groups were combined into 'some college', as there were too few

© 2026 Lemp, JM et al. JAMA Health Forum.

college graduates within this age range to estimate a separate distribution. This resulted in a total of 92 distinct groups for which beta distributions were fitted.

Alcohol consumption values are scaled to fall within the range 0 to 1 using the below transformation, where *GPD* = g per day, GPD_{min} = minimum g per day for the alcohol use category and GPD_{max} = maximum g per day for the alcohol use category. The small constants 10^{-10} and 10^{-9} are included to ensure numerical stability.

$$scaledGPD = \frac{(GPD - GPD_{min}) + 10^{-10}}{(GPD_{max} - GPD_{min}) + 10^{-9}}$$

In the microsimulation, individuals are allocated sampled values from the beta distributions corresponding to their subgroup. Values are initially sampled between 0 and 1 and rescaled to reflect the actual range of alcohol consumption (in g per day) for the relevant alcohol use category. The transformation used to rescale the values, where *sampledvalue* is the original value sampled between 0 and 1 is:

$$sampledGPD = \left((GPD_{max} - GPD_{min} + 10^{-10}) \cdot sampledvalue\right) + (GPD_{min} - 10^{-9})$$

To assess how well the beta distributions fit the data, the mean alcohol consumption values from the BRFSS (2000-2019) are compared with those produced by the simulation for each group to see if there are any large differences using a mean implausibility threshold of 3. This comparison indicated that adjustments were needed only for the group 'Alcohol use category 1 men aged 65 and over' across all race and ethnicity groups and within the high school degree or less and some college education groups. For these six groups, the shape and scale parameters of the beta distributions were refined by resampling and rerunning the model to identify parameters that best aligned with the target data. The full specification of beta distributions, including original values derived from BRFSS data and adjusted values for the refined groups is available online: https://figshare.com/s/1355ee5cf3c8bd81b0dd.

In secondary data analyses, we did not reliably identify distinct annual transitions between alcohol use categories during the COVID-19 pandemic compared to before; therefore, we applied the same validated ordinal alcohol use model to represent transitions for all years from 2000 to 2030.

Births and migration

Individuals aged 18 are added to the model in each year based on 2000-2021 ACS data and 2022-2030 projections. This is based on the number of 18-year-olds that are in the population in each corresponding year, subtracting the number of 18-year-olds that reported to have migrated into the US within the last year (based on ACS data), as migration was modelled separately.

Inward and outward migration rates are specified for each year. As no data on outward migration is available (i.e., the US does not routinely collect data on how many residents leave the country), migration rates are estimated through simulation in a pre-modelling step that adjusts the population counts to match those in the 2000-2021 ACS data and 2022-2030 projections and calculates the corresponding migration rates that would be required to achieve these counts. Migration rates are specific to 18-year-olds and 5 years age categories, sex, and race and ethnicity. Prior beliefs for inward migration are calculated based on 2000-2021 ACS

© 2026 Lemp, JM et al. JAMA Health Forum.

data and survey questions that asked individuals whether their place of residence was different one year prior to the survey. Outward migration rates are estimated as part of the pre-modelling simulation given the lack of data.

Applying screening and brief intervention expansion

In each year specified as an expansion year, all current alcohol users have the chance to progress through the alcohol screening and brief intervention (ASBI) care cascade, and—contingent on having attended a health check-up and being screening positive for exceeding the threshold for hazardous drinking—to be selected for a brief alcohol intervention.

Expansion scenarios are defined by the total number of alcohol screenings and brief interventions added. Since the microsimulation describes a synthetic cohort of 1 million adults in 2000, representing approximately 0.5% of the U.S. adult population, national expansion numbers were scaled to the simulated population. For example, 8M additional annual brief interventions nationally correspond to 40,000 additional interventions in the simulated cohort. This simulated number of additional interventions was held constant over time. Because the synthetic population grows proportionally with the underlying U.S. population, the scaling factor remains approximately constant, such that each simulated intervention represents a similar number of real-world individuals over time.

If a synthetic individual is sampled to receive a brief intervention *due to expansion of the screening and brief intervention contingent*, they change their alcohol consumption and heavy episodic drinking status based on an effect distribution from the literature.[40] Table S13 gives an overview of the brief intervention effect estimates implemented in the model. In the main analysis, changes in alcohol consumption were only applied to those with hazardous alcohol consumption (>20/>40 g per day for women/men).

Table S13. Policy effect estimates used in the microsimulation model.

Analysis	Brief intervention effect estimate (in g per day)[a]	Baseline consumption adjustment (in g per day)[b]	Brief intervention effect on heavy episodic drinking prevalence
Main analysis	M = -2.86, SE = 0.58	M = 0.023, SE = 0.005	RD = 0.07, SE = 0.026
Sensitivity analysis 1 (minimum, 2.5th percentile)	M = -1.72, SE = 0.35	M = 0.014, SE = 0.003	RD = 0.021, SE = 0.008
Sensitivity analysis 1 (maximum, 97.5th percentile)	M = -4.00, SE = 0.82	M = 0.032, SE = 0.007	RD = 0.120, SE = 0.044

Note: g per day = average grams of pure alcohol consumed per day. [a] The effect estimate distributions were calculated based on the primary meta-analysis conducted by Kaner et al. (2018),[40] which found that, after 12 months, participants receiving a brief intervention drank a mean 20 g alcohol a week less than those with minimal or no intervention (95% CI, 12 to 28; moderate-quality evidence; I^2 = 73%). [b] Meta-

© 2026 Lemp, JM et al. JAMA Health Forum.

regression analysis further showed that for every g/week increase in baseline consumption, the mean difference in consumption between brief intervention and minimal or no intervention participants increased by 0.16 g per week (95% CI, 0.10 to 0.23). Average baseline consumption was 244 g per week (i.e., 34.85 g per day).

All individuals newly receiving a brief intervention due to ASBI expansion were assigned a reduction in average alcohol consumption based on the effect distribution. In addition, among the subset of newly intervened individuals classified as engaging in heavy episodic drinking (HED) at baseline, a proportion corresponding to the pooled risk difference for HED reduction was randomly selected to transition to non-HED status. Changes in average alcohol consumption and HED status were sampled separately, such that transition out of HED was not deterministically linked to the magnitude of individual-level consumption reduction. Effects of existing interventions (i.e., if an individual was sampled to receive a brief intervention due to historical rates) are assumed to be already captured in historical alcohol use data.

Sampling for each step in ASBI care cascade (routine health visit, alcohol screening, and alcohol brief intervention) is based on empirical data from the National Survey on Drug Use and Health (NSDUH)[15] in the years 2013-2019. Specifically, three logistic regression models were fitted conditional on alcohol use category and age, sex, race and ethnicity, educational attainment, and if applicable, year (Table S14). Figure S9 shows the historical rates for each step in the cascade by sex and educational attainment (NSDUH data) as well as the simulated rates from the microsimulation that interpolated and projected trends into the future using regression-based sampling based on logistic regression models. Uncertainty around the regression coefficients was propagated across microsimulation runs (see *1.3.6. Uncertainty representation*).

When modelling the cascade, we first assigned probabilities of having a routine health visit to all individuals who currently drink alcohol. For those who were sampled as having had a routine health visit, we then proceeded to assign probabilities of being screened for hazardous alcohol use and selected individuals at historical coverage rates. In addition, in expansion scenarios that expanded the number of individuals being screened for alcohol use, we drew additional individuals and tracked whether they were selected for screening under “historical” or “expanded” rates. As the last step of the cascade, we assigned probabilities of receiving alcohol brief intervention to those that were screened and exhibited hazardous alcohol consumption levels (>20/>40 g per day for women/men), similarly, first, at historical coverage rates and additionally at expanded coverage rates. We assumed perfect sensitivity and specificity by allocating additional brief interventions only to those with hazardous drinking, whereas those with a higher alcohol consumption category were more likely to receive a brief intervention (Table S14).

As mentioned above, only individuals that received brief intervention either due to *expanded* screening or *expanded* brief intervention rates were then assigned a reduction in alcohol consumption (g per day) based on the empirical brief intervention effect distribution (Table S13). We accounted for each individual’s baseline alcohol consumption level by scaling the reduction

© 2026 Lemp, JM et al. JAMA Health Forum.

by how much one's baseline consumption deviated from the average baseline consumption, i.e., synthetic individuals with higher daily consumption levels experience larger absolute reductions in their alcohol use (Figure S10). This adjustment is based on meta-regression results that showed that for every g/week increase in baseline consumption, the mean difference in consumption between brief intervention and minimal or no intervention participants increased by 0.16 g/week (95% CI 0.10 to 0.23).[40] Additionally, among individuals who are selected to receive a brief intervention due to expanded rates and who also exhibit heavy episodic drinking (HED), we randomly select individuals to change their HED status corresponding to the number determined by the empirical effect distribution (Table S13). For sensitivity analyses, we sampled the mean difference at the 2.5th and 97.5th rank from the population effect distribution and renormalized the standard error (i.e., the standard error of the population was rescaled to reflect the standard error of the selected mean difference).

Table S14. Ordinal Logistic Regression model for assigning individual health visit, alcohol screening, and brief intervention probabilities, informed by NSDUH data (2013-2019).

	**Model**		
**Coefficient**	**Health visit (n = 159,539)**	**Alcohol screening (n = 129,163)**	**Brief intervention (n = 7,529)**
Men[a]	0.33 (0.31, 0.35)	0.82 (0.78, 0.86)	2.57 (1.88, 3.52)
Age 25-64[b]	1.25 (1.14, 1.37)	0.66 (0.62, 0.71)	3.04 (1.93, 4.81)
Age 65+[b]	3.02 (2.21, 4.11)	0.36 (0.32, 0.4)	2.79 (1.53, 5.09)
Some college[c]	0.9 (0.83, 0.97)	0.59 (0.55, 0.64)	1.4 (0.82, 2.39)
≤High school[c]	0.64 (0.58, 0.7)	0.36 (0.34, 0.39)	1.98 (1.14, 3.46)
Hispanic[d]	0.72 (0.67, 0.78)	1.12 (1.05, 1.2)	0.96 (0.6, 1.55)
Non-Hispanic Other[d]	0.75 (0.68, 0.83)	0.97 (0.89, 1.06)	1.12 (0.64, 1.96)
Non-Hispanic White[d]	1.12 (1.05, 1.2)	1.3 (1.23, 1.38)	0.85 (0.56, 1.29)
Alcohol cat. II[e]	0.82 (0.75, 0.9)	1.48 (1.37, 1.59)	·
Alcohol cat. III[e]	0.72 (0.65, 0.79)	1.29 (1.17, 1.42)	1.74 (1.41, 2.16)
Age 25-64* Some college	0.84 (0.76, 0.93)	1.31 (1.2, 1.43)	0.61 (0.36, 1.06)
Age 65+* Some college	1.02 (0.73, 1.44)	1.54 (1.34, 1.76)	0.86 (0.38, 1.95)
Age 25-64* ≤High school	0.85 (0.76, 0.94)	1.66 (1.52, 1.81)	0.5 (0.28, 0.87)
Age 65+* ≤High school	1.03 (0.76, 1.39)	1.9 (1.65, 2.19)	0.57 (0.23, 1.4)
Men*Age 25-64	1.18 (1.09, 1.27)	1.35 (1.27, 1.44)	·
Men*Age 65+	2.28 (1.76, 2.95)	1.61 (1.45, 1.79)	·
Year	·	1.08 (1.06, 1.09)	1.01 (0.97, 1.06)
Year*Some college	·	0.98 (0.96, 1)	·
Year* ≤High school	·	0.93 (0.91, 0.95)	·
Men*Some college	·	·	1.09 (0.69, 1.72)

© 2026 Lemp, JM et al. JAMA Health Forum.

Men * ≤High school	·	·	0.5 (0.31, 0.79)

Note: ≤High school: High school degree or less, ≥College: College degree or more. [a] ref = women, [b] ref=age 18-24, [c] ref = ≥College, [d] ref = non-Hispanic Black, [e] ref = Alcohol category I. For alcohol screening model, the sample is restricted to those who attended a routine health visit. For brief intervention model, the sample is restricted to those who have been screened and exhibit hazardous alcohol consumption (alcohol category II and III).

© 2026 Lemp, JM et al. JAMA Health Forum.

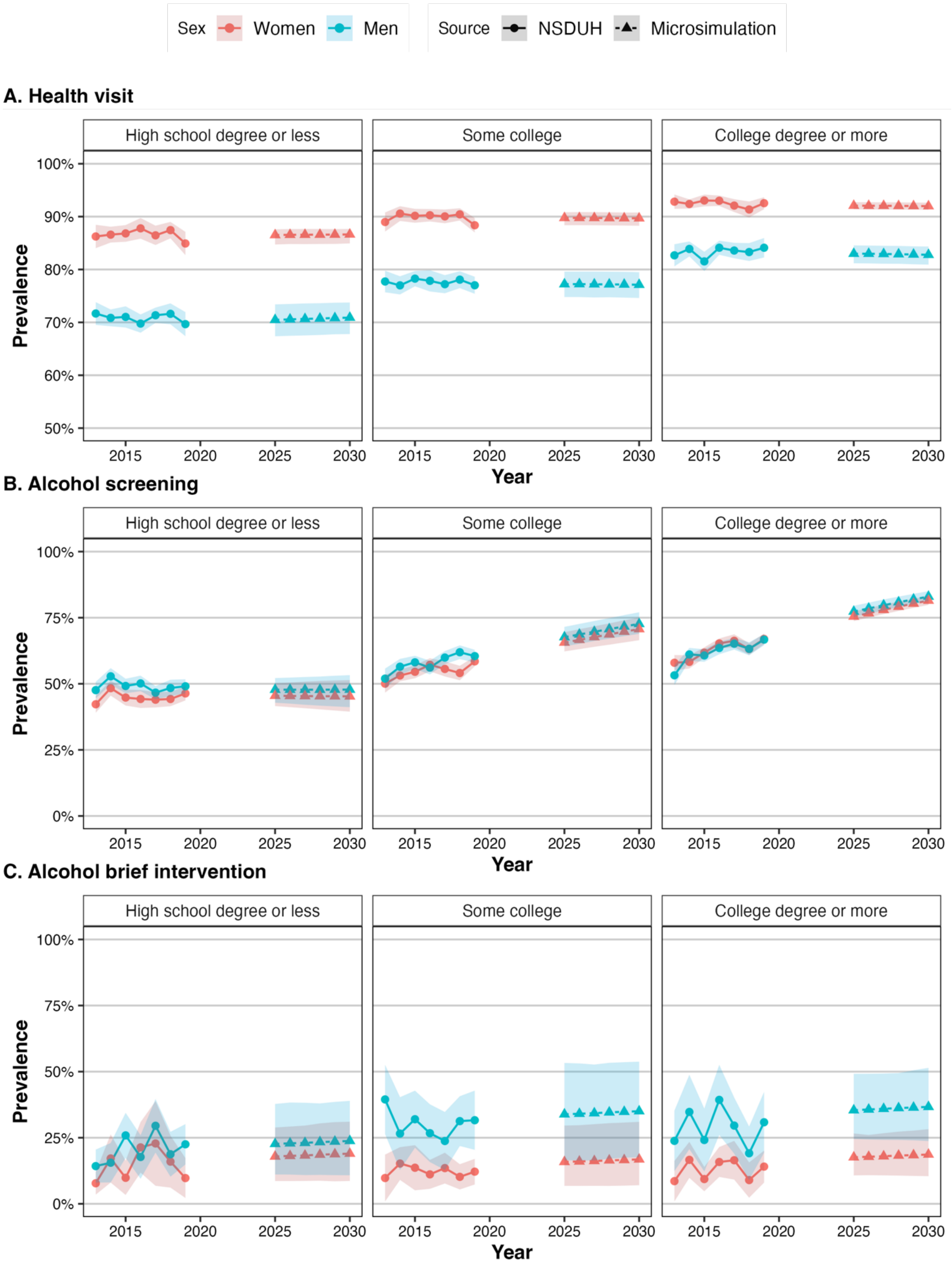

Figure S9. Proportion of individuals who progress through the ASBI care cascade, i.e., rates for annual routine health visits (A), alcohol screening (B; among those with a health visit), and brief intervention (C; among those with a positive screening outcome) based on empirical NSDUH

© 2026 Lemp, JM et al. JAMA Health Forum.

data (round shape, solid line) and across 70 microsimulation runs (triangular shape, dotted line; reference scenario) using regression models described in Table S14. The shaded area represents 95% credibility intervals which were propagated in our analyses (see 1.3.6 Uncertainty representation).

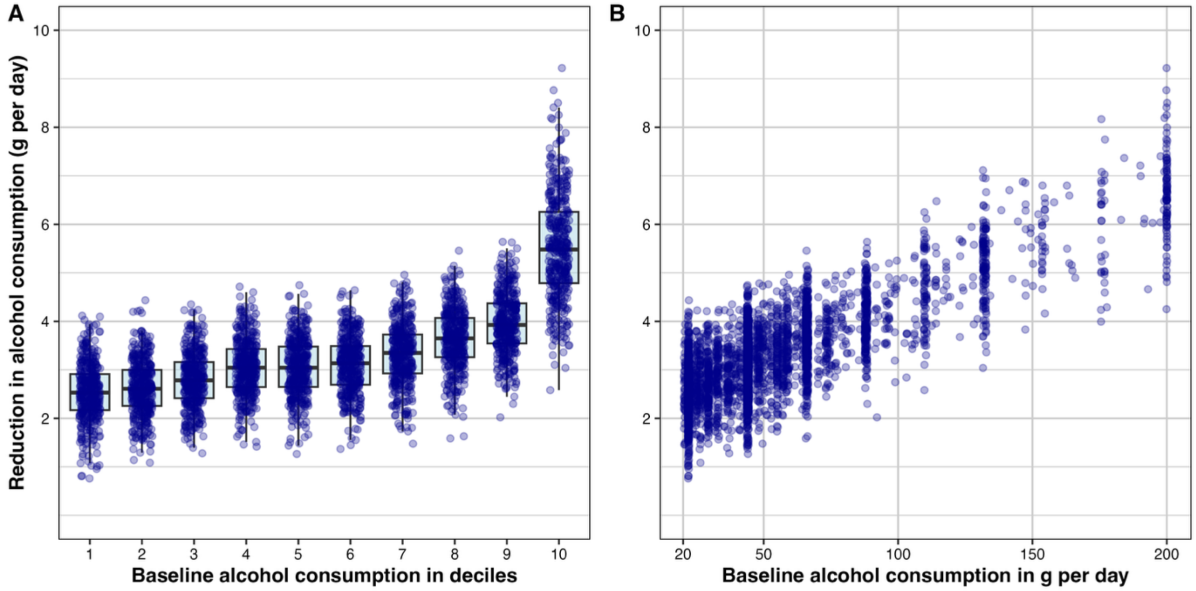

Figure S10. N = 4,000. Visualization of individual-level reduction in level of alcohol consumption (in average grams of pure alcohol consumed per day, i.e., g per day) applying the mean treatment effect per the main analysis (M = -2.86, SE = 0.58) to those with hazardous alcohol consumption. The lower limit for hazardous alcohol consumption for women is 20 g per day. Scatterplots are displayed by baseline consumption in g per day (A) and deciles of baseline consumption (B).

1.3.6 Uncertainty representation

Two different types of uncertainty are accounted for: 1) uncertainty through stochasticity (see 1.2.1 Stochasticity) and 2) parameter uncertainty. Parameter uncertainty refers to different posterior beliefs about the parameters of the calibrated education and alcohol models, mortality base rates, draws from the COVID-19-specific education transition, risk function and Dirichlet function model parameters, as well as uncertainty of any parameters that determine the policy effect estimate.

To identify the number of simulation runs required to obtain robust model estimates in the primary model outcome of interest (i.e., YLL per 100,000) for both types of uncertainty, we investigated the impact of running multiple iterations of the microsimulation on model precision. Our aim was to eliminate most of the noise that originates from random number generation and quantify the uncertainty that stems from different posterior distributions of the calibrated education and alcohol models, COVID-19-specific education transition, parameters used for mortality estimation and policy effect estimation.

First, uncertainty through stochasticity was explored by running 20 iterations of the microsimulation with unique random number seeds but identical parameter setting. We then

© 2026 Lemp, JM et al. JAMA Health Forum.

calculated the mean YLL per 100,000 by group (i.e., education and sex, and race and ethnicity and sex) across one, two, three etc. up to 20 simulation runs. Figure S11 illustrates the deviation of the calculated mean YLL in the year 2030 by the number of simulation runs taken into account compared to the mean alcohol consumption level across all 20 iterations. Across education categories, deviations varied between -26 to 36 YLL per 100,000 in men and between to -28 to 38 YLL per 100,000 in women. Across race and ethnicity categories, deviations varied between -67 and 88 YLL per 100,000 in men and -57 and 93 YLL per 100,000 in women. For most groups, mean YLL per 100,000 estimates stabilised after about 15 iterations with variation within +/-5 YLL per 100,000. Given fluctuations in selected groups such as men with some college education, we chose 20 iterations as the default modelling choice to obtain robust model estimates.

When presenting results from the microsimulation in the manuscript (i.e., changes in alcohol use categories and YLL per 100,000 by education and sex over time and by race and ethnicity and sex over time), uncertainty through stochasticity is therefore controlled for by using the average across n = 20 iterations with unique random number seeds.

Second, parameter uncertainty was explored by running 100 iterations of the microsimulation, each with a unique combination of samples drawn from the calibrated posterior distributions of the education and alcohol transition models, the COVID-19-specific education transitions, the risk functions, the Dirichlet function model, and the mortality base rates, as well as policy-specific parameters drawn from their population distributions.

Mortality base rates samples were generated based on the same set of education, alcohol transition, COVID-19-specific education, risk functions and the Dirichlet models. Specifically, for each iteration, one education model, one alcohol transition model, and one mortality base rate were randomly sampled from their respective posterior distributions, while COVID-19-specific education transition model, risk functions and Dirichlet function model were drawn from their respective model parameter distributions. Lastly, to incorporate uncertainty for each modelled policy, policy-specific parameters that influence the change in alcohol use (following policy implementation) were likewise systematically varied across unique combinations.

For the screening and brief intervention model, the regression coefficients used to determine individual probabilities at each step of the screening and brief intervention cascade were drawn from their estimated distributions using Latin Hypercube Sampling (Table S14). Each Latin Hypercube sample represented a unique combination of regression coefficients, reflecting joint uncertainty across regression logistic regression model parameters. We did not sample a new mean brief intervention effect or redefine its distribution for each parameter combination. Instead, we conducted separate sensitivity analyses to illustrate uncertainty in the brief intervention effect size independently from other parameter uncertainties (Table S13).

We calculated the mean YLL per 100,000 by group (i.e., education and sex, and race and ethnicity and sex) across one, two, three etc. up to 100 simulation runs. Figure S12 illustrates the deviation of the calculated mean YLL per 100,000 and Figure S13 illustrates the deviation of the mean reduction in YLL per 100,000 (in scenario 3 compared to the reference scenario) from the mean across all 100 iterations, by the number of simulation runs taken into account. Across groups, mean YLL estimates and reduction in YLL stabilised after about 70 iterations with a

© 2026 Lemp, JM et al. JAMA Health Forum.

variation within +/-3.2 YLL per 100,000 and +/-2.2 YLL per 100,000, respectively. Therefore, 70 iterations were chosen to be sufficient for obtaining robust model estimates. We acknowledge that this approach does not account for the total uncertainty associated with the posterior distributions of the calibrated education and alcohol transition models. However, given the overall limited variation observed, we expect that this approach still reflects a reasonable representation of uncertainty.

When presenting results from the microsimulation in the manuscript (i.e., changes in alcohol use categories and YLL per 100,000 by education and sex and by race and ethnicity and sex over time), uncertainty through parameter uncertainty is displayed in credible intervals (CrI) by presenting the 2.5th and 97.5th ranked values across n = 70 iterations using unique combinations of the calibrated versions of the education and alcohol models, mortality base rates, draws from the COVID-19-specific education transitions, risk functions and Dirichlet function model parameters, and randomly sampled policy effect parameters. Mean effect estimates were calculated by averaging across these 70 parameter combinations. In combination with n = 20 iterations with unique random number seeds to control for stochasticity (see above), we run n = 1,400 model iterations for each policy scenario in total.

© 2026 Lemp, JM et al. JAMA Health Forum.

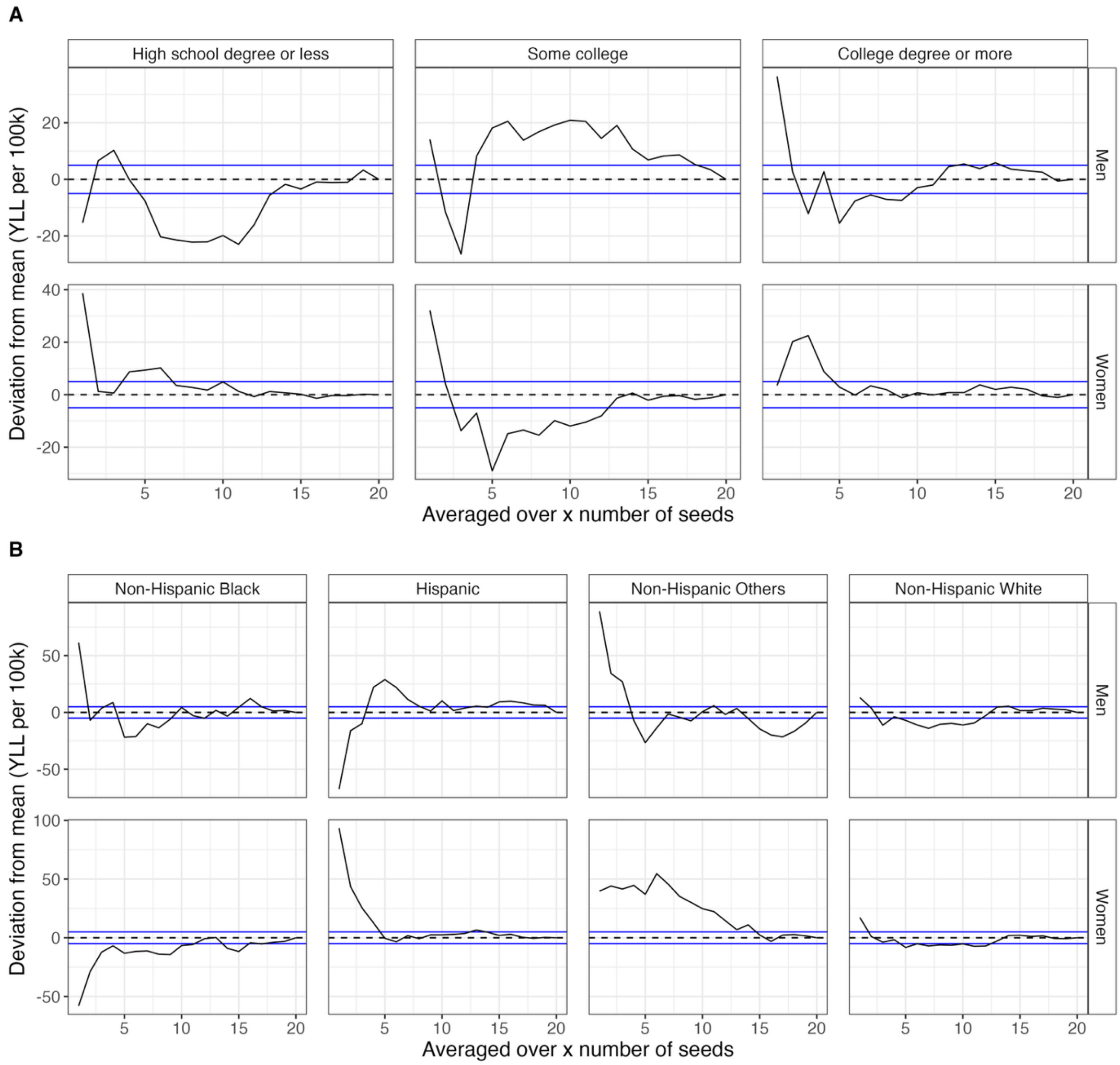

Figure S11. Deviation in mean YLL per 100,000 in 2030 by number of simulation runs with unique random number seed compared to the mean YLL per 100,000 across 20 simulation runs by sex and education (A) and sex and race and ethnicity (B). Model configuration: main policy setting, scenario 3 (+8M brief interventions). Parameters (i.e., calibrated posterior distributions of the education and alcohol transition models, the COVID-19-specific education transition, the risk function, the Dirichlet function model, the mortality base rates, and the screening and brief intervention cascade regression models) held constant across simulation runs.

© 2026 Lemp, JM et al. JAMA Health Forum.

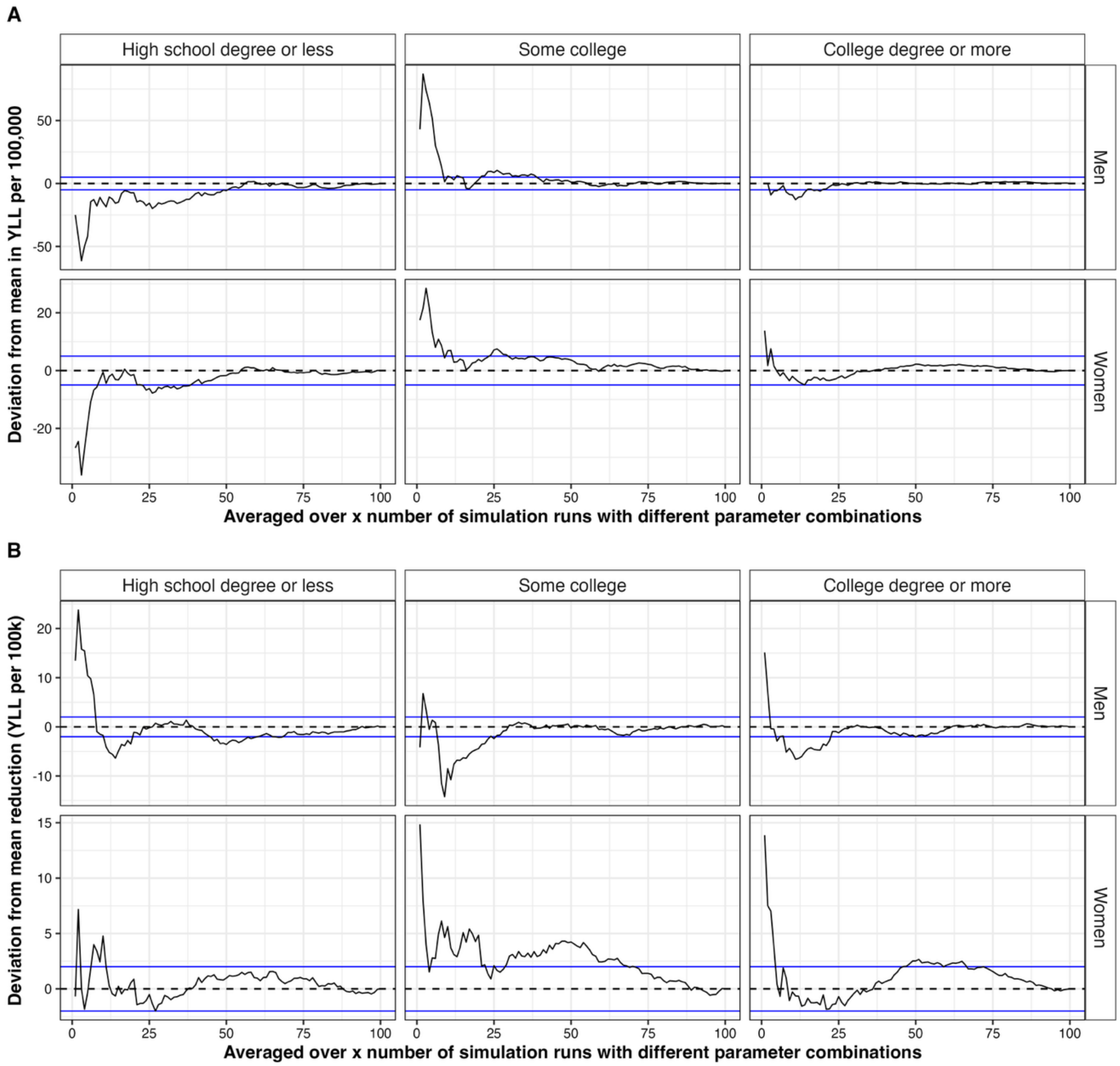

Figure S12. Deviation in mean YLL per 100,000 (A) and deviation in mean reduction in YLL per 100,000 (B) in 2030 by number of simulation runs with unique combinations of parameter settings (i.e., calibrated posterior distributions of the education and alcohol transition models, the COVID-19-specific education transition, the risk function, the Dirichlet function model, the mortality base rates, and the screening and brief intervention cascade regression models) compared to the mean YLL per 100,000 across 100 simulation runs, stratified by education. Model configuration: main policy setting, scenario 3 (+8M brief interventions), reduction calculated compared to no expansion scenario. Each unique parameter combination is averaged across 20 random number seeds.

© 2026 Lemp, JM et al. JAMA Health Forum.

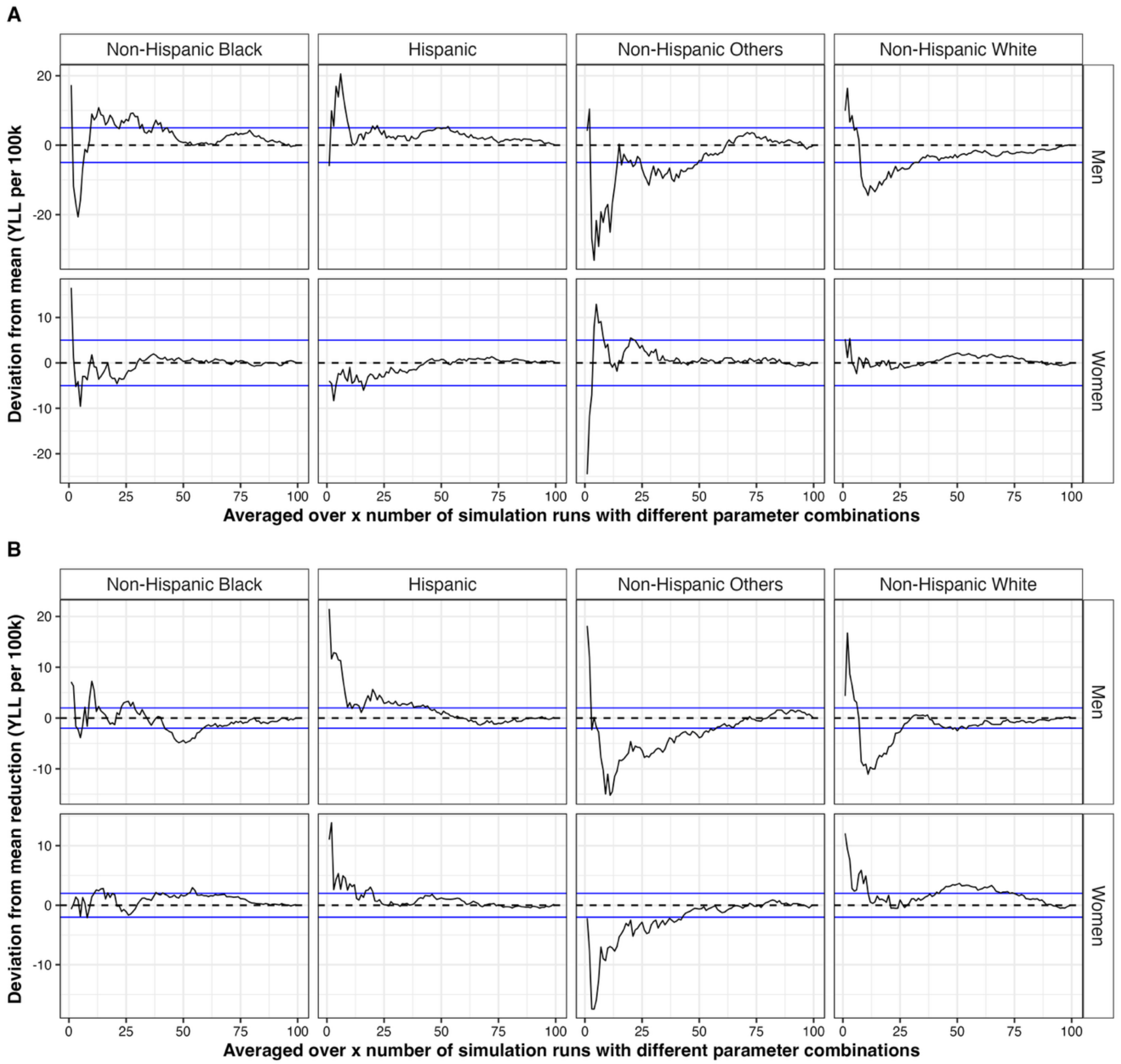

Figure S13. Deviation in mean YLL per 100,000 (A) and deviation in mean reduction in YLL per 100,000 (B) in 2030 by number of simulation runs with unique combinations of parameter settings (i.e., calibrated posterior distributions of the education and alcohol transition models, the COVID-19-specific education transition, the risk function, the Dirichlet function model, the mortality base rates, and the screening and brief intervention cascade regression models) compared to the mean YLL per 100,000 across 100 simulation runs, stratified by race and ethnicity. Model configuration: main policy setting, scenario 3 (+8M brief interventions), reduction calculated compared to the reference scenario. Each unique parameter combination is averaged across 20 random number seeds.

© 2026 Lemp, JM et al. JAMA Health Forum.

1.3.7 Model assumptions

The microsimulation model is based on four global assumptions needed to emulate and reduce real-world complexity: i) the data used to inform synthetic population and model parameters are valid and generalizable to the adult US population and its subgroups across time; ii) the structure of the Markov and ordinal model used to inform transition processes in education and alcohol use, respectively, are reasonable representations of the corresponding population dynamics;[4,7] iii) education is a sufficient indicator of socioeconomic status (SES) in the United States, and iv) the implemented policy mechanisms provide a valid approximation of real-world policy or expansion effects.

Within i) the global assumption of generalizability we deem the following as most critical to the microsimulation: i.i) Census and ACS data used to inform the baseline population, fertility and inward migration are representative of the general population of the US within subgroups defined by age, sex, education, and race and ethnicity; i.ii) alcohol consumption informed by BRFSS data captures alcohol use adequately and equally well across all subgroups after applying an individual-level up-shifting procedure to match BRFSS data to APC data;[5] i.iii) risk functions, which are based on available data from meta-analyses that may incorporate data from other countries, are an accurate representation of the relationship between alcohol consumption and mortality in the United States; and i.iv) policy effect parameters derived from international or non-U.S. studies are assumed to be generalizable to policy effects within the US context.

Regarding ii) the representation of population dynamics, the following assumptions are deemed as most critical: ii.i) drinking behaviours are relatively stable over time as represented in the pseudo-panel generated to inform the ordinal model of alcohol use transitions; ii.ii) the distribution of alcohol consumption in g per day within alcohol use categories, which is used to reassign consumption after individuals transition between alcohol use categories, is relatively stable over time; ii.iii) transition probabilities between levels of education attainment were reasonably stable for the modelled period and the structure of the Markov model used to inform transition processes in education is a reasonable representation of the corresponding population dynamics.

Regarding iii) education as an accurate and sufficient representation of SES in the US we assume iii.i) little to no changes in the socioeconomic interpretation of each education category for the modelled period; and iii.ii) education is stable after the age of 34 and constitutes an equally precise indicator of SES across the life trajectory.

For the screening and brief intervention model, we additionally deem the following as critical to the presented results: iv.ii.i) Coverage rates for health visits, alcohol screening, and brief intervention, as derived from NSDUH data,[16] are assumed to be a valid and generalizable representation of the screening and brief intervention cascade across US adult population subgroups, iv.ii.ii) the regression models used to assign probabilities of receiving of screening and brief intervention in the synthetic population can be accurately informed by cross-sectional NSDUH data that measures receipt of brief intervention and post-intervention alcohol consumption levels concurrently; iv.ii.iii) the status quo (no expansion scenario) accurately reflects the US population that has received brief intervention according to historical rates;

© 2026 Lemp, JM et al. JAMA Health Forum.

iv.ii.iv) brief intervention effect parameters derived in part from international and non-U.S. studies are assumed to be generalizable to brief intervention effects within the US context; and iv.ii.v) individual-level brief intervention effects are assumed to systematically vary solely conditional on baseline alcohol consumption level, and are independent of sociodemographic characteristics and from whether someone has received an intervention previously[37].

1.4 References

1. Probst C, Buckley C, Lasserre AM, et al. Simulation of Alcohol Control Policies for Health Equity (SIMAH) Project: Study Design and First Results. *Am J Epidemiol*. 2023;192(5):690-702. doi:10.1093/aje/kwad018

2. Grimm V, Railsback SF, Vincenot CE, et al. The ODD Protocol for Describing Agent-Based and Other Simulation Models: A Second Update to Improve Clarity, Replication, and Structural Realism. *J Artif Soc Soc Simul*. 2020;23(2):7. doi:10.18564/jasss.4259

3. Ruggles S, Flood S, Goeken R, Schouweiler M, Sobek M. IPUMS USA: Version 12.0. Published online 2022. doi:10.18128/D010.V12.0

4. Buckley C, Bright S, Purshouse R, et al. Disparities in educational mobility in the US by race and ethnicity, sex, and household income: results from a prospective cohort study 1999 to 2019. *SocArXiv*. Preprint posted online March 13, 2025. doi:10.31235/osf.io/rjx92_v1

5. Buckley C, Brennan A, Kerr WC, et al. Improved estimates for individual and population-level alcohol use in the United States, 1984-2020. *Int J Alcohol Drug Res*. 2022;10(1):24-33. doi:10.7895/ijadr.383

6. World Health Organization. *International Guide for Monitoring Alcohol Consumption and Related Harm*. World Health Organization; 2001.

7. Puka K, Buckley C, Mulia N, et al. Behavioral stability of alcohol consumption and socio-demographic correlates of change among a nationally representative cohort of US adults. *Addiction*. Published online August 28, 2022:add.16024. doi:10.1111/add.16024

8. Cook WK, Kerr WC, Zhu Y, et al. Alcoholic beverage types consumed by population subgroups in the United States: Implications for alcohol policy to address health disparities. *Drug Alcohol Rev*. 2024;43(4):946-955. doi:10.1111/dar.13819

9. National Center for Health Statistics UC for DC and P. National Vital Statistics System. Accessed June 30, 2023. https://www.cdc.gov/nchs/nvss/index.htm

10. Manning WG, Blumberg L, Moulton LH. The demand for alcohol: The differential response to price. *J Health Econ*. 1995;14(2):123-148. doi:10.1016/0167-6296(94)00042-3

11. Lovelace R, Birkin M, Ballas D, Van Leeuwen E. Evaluating the Performance of Iterative Proportional Fitting for Spatial Microsimulation: New Tests for an Established Technique. *J Artif Soc Soc Simul*. 2015;18(2):21. doi:10.18564/jasss.2768

© 2026 Lemp, JM et al. JAMA Health Forum.

12. US Census Bureau. US Census. https://www.census.gov/programs-surveys/decennial-census.html

13. Institute for Social Research, University of Michigan, Ann Arbor Survey Research Center. Panel Study of Income Dynamics, public use dataset. Accessed June 30, 2023. https://psidonline.isr.umich.edu

14. Centers for Disease Control. Behavioral Risk Factor Surveillance System (BRFSS). Accessed December 28, 2022. https://www.cdc.gov/brfss/

15. Substance Abuse and Mental Health Services Administration. National Survey on Drug Use and Health (NSDUH). Accessed October 9, 2024. https://www.samhsa.gov/data/data-we-collect/nsduh-national-survey-drug-use-and-health

16. Ingram DD, Parker JD, Schenker N, et al. United States Census 2000 population with bridged race categories. *Vital Health Stat 2*. 2003;(135):1-55.

17. National Center for Health Statistics. Documentation for Vintage 2020 Bridged-Race Postcensal Population Estimates for Calculating Vital Rates. Accessed October 7, 2024. https://www.cdc.gov/nchs/data/nvss/bridged_race/Documentation-Bridged-PostcenV2020.pdf

18. US Census Bureau. *Methodology, Assumptions, and Inputs for the 2023 National Population Projections*. U.S. Census Bureau; 2023. https://www2.census.gov/programs-surveys/popproj/technical-documentation/methodology/methodstatement23.pdf

19. US Census Bureau. 2023 National Population Projections. Accessed October 28, 2024. https://www.census.gov/data/datasets/2023/demo/popproj/2023-popproj.html

20. US Census Bureau. National Population by Characteristics: 2020-2024. Accessed October 28, 2024. https://www.census.gov/data/datasets/time-series/demo/popest/2020s-national-detail.html

21. XGBoost Developers. XGBoost Parameters — xgboost 3.2.0 documentation. 2025. Accessed May 11, 2026. https://xgboost.readthedocs.io/en/stable/parameter.html#parameters-for-tree-booster

22. Llamosas-Falcón L, Probst C, Buckley C, et al. Sex-specific association between alcohol consumption and liver cirrhosis: An updated systematic review and meta-analysis. *Front Gastroenterol*. 2022;1:1005729. doi:10.3389/fgstr.2022.1005729

23. Llamosas-Falcón L, Probst C, Buckley C, et al. How does alcohol use impact morbidity and mortality of liver cirrhosis? A systematic review and dose–response meta-analysis. *Hepatol Int*. 2024;18(1):216-224. doi:10.1007/s12072-023-10584-z

24. Carr T, Kilian C, Llamosas-Falcón L, et al. The risk relationships between alcohol consumption, alcohol use disorder and alcohol use disorder mortality: A systematic review and meta-analysis. *Addiction*. 2024;119(7):1174-1187. doi:10.1111/add.16456

© 2026 Lemp, JM et al. JAMA Health Forum.

25. Lange S, Kim KV, Lasserre AM, et al. Sex-Specific Association of Alcohol Use Disorder With Suicide Mortality: A Systematic Review and Meta-Analysis. *JAMA Netw Open*. 2024;7(3):e241941. doi:10.1001/jamanetworkopen.2024.1941

26. Llamosas-Falcón L, Rehm J, Bright S, et al. The Relationship Between Alcohol Consumption, BMI, and Type 2 Diabetes: A Systematic Review and Dose-Response Meta-analysis. *Diabetes Care*. 2023;46(11):2076-2083. doi:10.2337/dc23-1015

27. Zhao J, Stockwell T, Roemer A, Naimi T, Chikritzhs T. Alcohol Consumption and Mortality From Coronary Heart Disease: An Updated Meta-Analysis of Cohort Studies. *J Stud Alcohol Drugs*. 2017;78(3):375-386. doi:10.15288/jsad.2017.78.375

28. Larsson SC, Wallin A, Wolk A, Markus HS. Differing association of alcohol consumption with different stroke types: a systematic review and meta-analysis. *BMC Med*. 2016;14(1):178. doi:10.1186/s12916-016-0721-4

29. Liu F, Liu Y, Sun X, et al. Race- and sex-specific association between alcohol consumption and hypertension in 22 cohort studies: A systematic review and meta-analysis. *Nutr Metab Cardiovasc Dis*. 2020;30(8):1249-1259. doi:10.1016/j.numecd.2020.03.018

30. World Health Organization. Global Status Report on Alcohol and Health. 2018. Accessed March 18, 2022. https://apps.who.int/iris/rest/bitstreams/1151838/retrieve

31. Jiang H, Rehm J, Probst C, Tran A, Lange S, Llamosas-Falcón L. A practical guide to conducting dose-response meta-analyses in epidemiology. *Methodology*. 2025;21(2):144-160. doi:10.5964/meth.14733

32. Llamosas-Falcón L, Shield KD, Gelovany M, et al. Impact of alcohol on the progression of HCV-related liver disease: A systematic review and meta-analysis. *J Hepatol*. 2021;75(3):536-546. doi:10.1016/j.jhep.2021.04.018

33. Probst C, Zhu Y, Kilian C, Kerr W, Rehm J. Educational attainment as a potential effect modifier of alcohol use and 100% alcohol-attributable mortality in the United States—A longitudinal analysis of mortality linked survey data from 1997 to 2018. *Addiction*. 2025;120(6):1143-1155. doi:10.1111/add.16774

34. Zhu Y, Llamosas-Falcón L, Kerr W, Puka K, Probst C. Differential Associations of Alcohol Use With Ischemic Heart Disease Mortality by Socioeconomic Status in the US, 1997-2018. *JAMA Netw Open*. 2024;7(2):e2354270. doi:10.1001/jamanetworkopen.2023.54270

35. Jabot F, Faure T, Dumoulin N. EasyABC: performing efficient approximate Bayesian computation sampling schemes using R. *Methods Ecol Evol*. 2013;4(7):684-687. doi:10.1111/2041-210X.12050

36. Andrianakis I, Vernon IR, McCreesh N, et al. Bayesian History Matching of Complex Infectious Disease Models Using Emulation: A Tutorial and a Case Study on HIV in Uganda. Wu H, ed. *PLoS Comput Biol*. 2015;11(1):e1003968. doi:10.1371/journal.pcbi.1003968

37. Ruhm CJ, Jones AS, McGeary KA, et al. What U.S. data should be used to measure the price elasticity of demand for alcohol? *J Health Econ*. 2012;31(6):851-862. doi:10.1016/j.jhealeco.2012.08.002

© 2026 Lemp, JM et al. JAMA Health Forum.

38. Fogarty J. The demand of beer, wine and spirits: a survey of the literature. *J Econ Surv*. 2010;24(3):428-478. doi:10.1111/j.1467-6419.2009.00591.x

39. Wagenaar AC, Salois MJ, Komro KA. Effects of beverage alcohol price and tax levels on drinking: a meta-analysis of 1003 estimates from 112 studies. *Addiction*. 2009;104(2):179-190. doi:10.1111/j.1360-0443.2008.02438.x

40. Kaner EF, Beyer FR, Muirhead C, et al. Effectiveness of brief alcohol interventions in primary care populations. Cochrane Drugs and Alcohol Group, ed. *Cochrane Database Syst Rev*. 2018;2018(6). doi:10.1002/14651858.CD004148.pub4

41. Kilian C, Lemp JM, Llamosas-Falcón L, et al. Reducing alcohol use through alcohol control policies in the general population and population subgroups: a systematic review and meta-analysis. *eClinicalMedicine*. 2023;59:101996. doi:10.1016/j.eclinm.2023.101996

42. Guindon GE, Zhao K, Fatima T, et al. Prices, taxes and alcohol use: a systematic umbrella review. *Addiction*. 2022;117(12):3004-3023. doi:10.1111/add.15966

© 2026 Lemp, JM et al. JAMA Health Forum.

eTable 1. Simulated prevalence of alcohol use categories by sex and education (standard effect).

			Category I alcohol use		Category II alcohol use		Category III alcohol use		Hazardous alcohol use (combined Category II and III)	
**Year**	**Sex**	**Education**	**Prevalence in % (CrI)**	**Change in PP (CrI)**	**Prevalence in % (CrI)**	**Change in PP (CrI)**	**Prevalence in % (CrI)**	**Change in PP (CrI)**	**Prevalence in % (CrI)**	**Change in PP (CrI)**
Reference scenario (no expansion)										
2000	M	≤High school	61.5	.	4.7	.	6.3	.	10.9	.
2000	M	Some college	67.6	.	5.3	.	5.9	.	11.2	.
2000	M	≥College	74.3	.	5.3	.	3.2	.	8.5	.
2000	W	≤High school	55.3	.	3.3	.	2.0	.	5.3	.
2000	W	Some college	64.8	.	4.9	.	2.6	.	7.5	.
2000	W	≥College	69.0	.	5.9	.	2.6	.	8.5	.
2030	M	≤High school	58.4 (58.3, 58.6)	.	5.1 (5.1, 5.2)	.	4.5 (4.4, 4.7)	.	9.6 (9.5, 9.9)	.
2030	M	Some college	62.8 (62.6, 62.9)	.	5.1 (5.1, 5.1)	.	3.4 (3.4, 3.5)	.	8.5 (8.4, 8.6)	.
2030	M	≥College	66.4 (66.4, 66.5)	.	6.1 (6.1, 6.2)	.	5.5 (5.4, 5.6)	.	11.6 (11.5, 11.7)	.
2030	W	≤High school	54.9 (54.7, 55.1)	.	4.0 (3.9, 4.1)	.	2.7 (2.6, 2.9)	.	6.7 (6.5, 6.9)	.
2030	W	Some college	59.9 (59.8, 60.1)	.	3.9 (3.8, 4.0)	.	2.0 (2.0, 2.1)	.	5.9 (5.8, 6.0)	.
2030	W	≥College	64.5 (64.4, 64.6)	.	5.1 (5.1, 5.1)	.	3.8 (3.7, 3.8)	.	8.9 (8.8, 8.9)	.
Scenario 1: Screening expansion by +20M										
2030	M	≤High school	58.6 (58.4, 58.8)	0.1 (0.0, 0.3)	5.0 (4.9, 5.1)	-0.1 (-0.2, 0.0)	4.4 (4.3, 4.6)	-0.1 (-0.2, 0.0)	9.5 (9.2, 9.7)	-0.2 (-0.3, 0.0)
2030	M	Some college	62.9 (62.8, 63.1)	0.1 (0.0, 0.3)	5.0 (4.9, 5.1)	-0.1 (-0.2, 0.0)	3.4 (3.3, 3.4)	-0.1 (-0.1, 0.0)	8.4 (8.2, 8.5)	-0.2 (-0.3, 0.0)
2030	M	≥College	66.6 (66.5, 66.7)	0.2 (0.0, 0.3)	6.1 (6.0, 6.1)	-0.1 (-0.1, 0.0)	5.4 (5.3, 5.5)	-0.1 (-0.2, -0.1)	11.4 (11.3, 11.6)	-0.2 (-0.3, -0.1)
2030	W	≤High school	55.0 (54.7, 55.3)	0.1 (0.0, 0.3)	3.9 (3.9, 4.0)	-0.1 (-0.2, 0.0)	2.7 (2.5, 2.8)	-0.1 (-0.2, 0.0)	6.6 (6.4, 6.8)	-0.1 (-0.4, 0.0)
2030	W	Some college	60.0 (59.9, 60.2)	0.1 (0.0, 0.2)	3.9 (3.8, 3.9)	0.0 (-0.1, 0.0)	2.0 (1.9, 2.1)	0.0 (-0.1, 0.0)	5.9 (5.7, 6.0)	-0.1 (-0.2, 0.0)
2030	W	≥College	64.6 (64.4, 64.7)	0.1 (0.0, 0.2)	5.1 (5.0, 5.1)	0.0 (-0.1, 0.0)	3.7 (3.6, 3.8)	-0.1 (-0.2, 0.0)	8.8 (8.7, 8.9)	-0.1 (-0.2, 0.0)
Scenario 2: Screening expansion by +20M, brief intervention expansion by +4M										
2030	M	≤High school	59.2 (58.9, 59.5)	0.8 (0.5, 1.0)	4.6 (4.4, 4.9)	-0.5 (-0.7, -0.3)	4.1 (4.0, 4.3)	-0.4 (-0.5, -0.3)	8.7 (8.4, 9.1)	-0.9 (-1.2, -0.6)
2030	M	Some college	63.8 (63.7, 64.1)	1.1 (0.9, 1.4)	4.3 (4.1, 4.5)	-0.8 (-1.0, -0.6)	3.0 (2.9, 3.1)	-0.5 (-0.6, -0.4)	7.3 (6.9, 7.5)	-1.3 (-1.6, -1.1)
2030	M	≥College	68.2 (67.9, 68.4)	1.8 (1.5, 2.0)	5.1 (5.0, 5.2)	-1.0 (-1.2, -0.9)	4.5 (4.2, 4.7)	-1.0 (-1.2, -0.8)	9.6 (9.3, 9.9)	-2.0 (-2.4, -1.7)
2030	W	≤High school	55.5 (55.2, 55.9)	0.7 (0.4, 0.9)	3.6 (3.4, 3.8)	-0.4 (-0.6, -0.2)	2.3 (2.2, 2.5)	-0.4 (-0.5, -0.2)	5.9 (5.7, 6.3)	-0.8 (-1.1, -0.4)
2030	W	Some college	60.6 (60.4, 60.8)	0.7 (0.4, 1.0)	3.4 (3.2, 3.7)	-0.5 (-0.7, -0.2)	1.7 (1.6, 1.8)	-0.3 (-0.4, -0.2)	5.1 (4.8, 5.5)	-0.8 (-1.2, -0.5)
2030	W	≥College	65.7 (65.4, 65.9)	1.2 (1.0, 1.4)	4.6 (4.4, 4.7)	-0.5 (-0.7, -0.4)	2.9 (2.8, 3.1)	-0.9 (-1.0, -0.7)	7.5 (7.2, 7.8)	-1.4 (-1.7, -1.1)
Scenario 3: Screening expansion by +20M, brief intervention expansion by +8M										
2030	M	≤High school	60.1 (59.8, 60.4)	1.7 (1.4, 1.9)	3.9 (3.8, 4.2)	-1.2 (-1.4, -0.9)	3.7 (3.6, 3.9)	-0.8 (-0.9, -0.7)	7.7 (7.4, 8.0)	-2.0 (-2.2, -1.7)
2030	M	Some college	64.7 (64.2, 64.9)	1.9 (1.5, 2.2)	3.5 (3.4, 3.9)	-1.6 (-1.7, -1.2)	2.7 (2.6, 2.9)	-0.8 (-0.9, -0.5)	6.2 (6.0, 6.7)	-2.3 (-2.5, -1.8)
2030	M	≥College	69.5 (68.9, 69.8)	3.1 (2.5, 3.4)	4.2 (4.0, 4.5)	-2.0 (-2.1, -1.7)	3.9 (3.7, 4.3)	-1.6 (-1.8, -1.2)	8.1 (7.7, 8.7)	-3.6 (-3.9, -2.9)
2030	W	≤High school	56.3 (55.9, 56.6)	1.4 (1.0, 1.7)	3.0 (2.8, 3.4)	-1.0 (-1.2, -0.6)	2.0 (1.8, 2.1)	-0.7 (-0.8, -0.6)	5.0 (4.6, 5.5)	-1.7 (-2.0, -1.3)

© 2026 Lemp, JM et al. JAMA Health Forum.

			Category I alcohol use		Category II alcohol use		Category III alcohol use		Hazardous alcohol use (combined Category II and III)	
**Year**	**Sex**	**Education**	**Prevalence in % (CrI)**	**Change in PP (CrI)**	**Prevalence in % (CrI)**	**Change in PP (CrI)**	**Prevalence in % (CrI)**	**Change in PP (CrI)**	**Prevalence in % (CrI)**	**Change in PP (CrI)**
2030	W	Some college	61.5 (61.1, 61.8)	1.5 (1.1, 1.8)	2.7 (2.4, 3.1)	-1.2 (-1.5, -0.8)	1.4 (1.3, 1.5)	-0.7 (-0.7, -0.5)	4.1 (3.7, 4.6)	-1.9 (-2.2, -1.3)
2030	W	≥College	67.1 (66.6, 67.3)	2.6 (2.1, 2.9)	3.6 (3.4, 4.0)	-1.5 (-1.7, -1.1)	2.2 (2.1, 2.3)	-1.6 (-1.7, -1.5)	5.8 (5.5, 6.3)	-3.1 (-3.4, -2.5)
Scenario 4: Universal screening and brief intervention										
2030	M	≤High school	61.1 (60.7, 61.3)	2.6 (2.4, 2.9)	3.2 (3.1, 3.4)	-1.9 (-2.1, -1.7)	3.2 (3.0, 3.4)	-1.3 (-1.4, -1.1)	6.5 (6.1, 6.8)	-3.2 (-3.4, -2.8)
2030	M	Some college	65.2 (64.6, 65.6)	2.4 (1.8, 2.8)	3.1 (2.8, 3.6)	-2.0 (-2.3, -1.5)	2.5 (2.3, 2.7)	-1.0 (-1.1, -0.7)	5.6 (5.2, 6.3)	-2.9 (-3.4, -2.2)
2030	M	≥College	69.9 (69.2, 70.4)	3.5 (2.8, 4.0)	3.8 (3.6, 4.3)	-2.3 (-2.5, -1.8)	3.7 (3.4, 4.1)	-1.8 (-2.1, -1.4)	7.5 (7.0, 8.3)	-4.1 (-4.6, -3.3)
2030	W	≤High school	57.2 (56.9, 57.4)	2.3 (2.1, 2.4)	2.3 (2.2, 2.5)	-1.7 (-1.8, -1.5)	1.6 (1.5, 1.8)	-1.1 (-1.2, -1.0)	3.9 (3.7, 4.3)	-2.8 (-3.0, -2.5)
2030	W	Some college	62.2 (61.9, 62.3)	2.2 (2.0, 2.4)	2.1 (2.0, 2.3)	-1.8 (-1.9, -1.7)	1.1 (1.0, 1.3)	-0.9 (-1.0, -0.8)	3.2 (3.0, 3.5)	-2.8 (-2.9, -2.5)
2030	W	≥College	67.8 (67.5, 68.0)	3.3 (3.0, 3.5)	3.0 (2.9, 3.2)	-2.1 (-2.2, -1.9)	1.8 (1.7, 2.0)	-2.0 (-2.1, -1.8)	4.8 (4.6, 5.2)	-4.1 (-4.3, -3.7)

Note: CrI: Credible interval, 2.5th and 97.5th rank of the simulated expansion effect across 70 model iterations, M: Men, W: Women, ≤High school: High school degree or less, ≥College: College degree or more, PP: percentage points. Standard brief intervention effect: M = -2.86, SE = 0.58.

© 2026 Lemp, JM et al. JAMA Health Forum.

eTable 2. Simulated prevalence of alcohol use categories by sex and race and ethnicity (standard effect).

			Category I alcohol use		Category II alcohol use		Category III alcohol use		Hazardous alcohol use (combined Category II and III)	
Year	Sex	Education	Prevalence in % (CrI)	Change in PP (CrI)	Prevalence in % (CrI)	Change in PP (CrI)	Prevalence in % (CrI)	Change in PP (CrI)	Prevalence in % (CrI)	Change in PP (CrI)
Reference scenario (no expansion)										
2000	M	Non-Hispanic Black	58.5	.	3.4	.	4.1	.	7.5	.
2000	M	Hispanic	68.1	.	4.0	.	5.7	.	9.7	.
2000	M	Non-Hispanic White	66.8	.	5.4	.	5.6	.	11.0	.
2000	W	Non-Hispanic Black	48.9	.	2.2	.	1.5	.	3.7	.
2000	W	Hispanic	49.1	.	2.8	.	1.3	.	4.1	.
2000	W	Non-Hispanic White	64.2	.	4.9	.	2.6	.	7.4	.
2030	M	Non-Hispanic Black	53.8 (53.7, 54.0)	.	2.9 (2.9, 3.0)	.	1.5 (1.4, 1.5)	.	4.4 (4.4, 4.5)	.
2030	M	Hispanic	58.5 (58.4, 58.6)	.	4.1 (4.1, 4.2)	.	3.0 (2.9, 3.1)	.	7.1 (7.0, 7.2)	.
2030	M	Non-Hispanic White	65.0 (64.9, 65.1)	.	6.5 (6.4, 6.5)	.	5.8 (5.7, 5.9)	.	12.2 (12.1, 12.4)	.
2030	W	Non-Hispanic Black	50.4 (50.3, 50.5)	.	2.3 (2.2, 2.3)	.	0.9 (0.8, 1.0)	.	3.2 (3.1, 3.3)	.
2030	W	Hispanic	54.5 (54.4, 54.6)	.	3.0 (2.9, 3.0)	.	1.6 (1.6, 1.7)	.	4.6 (4.5, 4.7)	.
2030	W	Non-Hispanic White	63.6 (63.4, 63.7)	.	5.4 (5.3, 5.4)	.	3.9 (3.7, 4.0)	.	9.2 (9.1, 9.4)	.
Scenario 1: Screening expansion by +20M										
2030	M	Non-Hispanic Black	53.9 (53.8, 54.1)	0.1 (-0.1, 0.2)	2.9 (2.8, 2.9)	-0.1 (-0.1, 0.0)	1.4 (1.4, 1.5)	0.0 (-0.1, 0.0)	4.3 (4.2, 4.4)	-0.1 (-0.2, 0.0)
2030	M	Hispanic	58.6 (58.5, 58.8)	0.1 (0.0, 0.2)	4.1 (4.0, 4.1)	-0.1 (-0.1, 0.0)	3.0 (2.9, 3.0)	0.0 (-0.1, 0.0)	7.0 (6.9, 7.1)	-0.1 (-0.2, 0.0)
2030	M	Non-Hispanic White	65.2 (65.0, 65.4)	0.2 (0.0, 0.3)	6.4 (6.3, 6.4)	-0.1 (-0.2, 0.0)	5.7 (5.5, 5.8)	-0.1 (-0.2, -0.1)	12.1 (11.8, 12.2)	-0.2 (-0.4, -0.1)
2030	W	Non-Hispanic Black	50.5 (50.4, 50.6)	0.1 (-0.1, 0.2)	2.2 (2.2, 2.3)	0.0 (-0.1, 0.0)	0.9 (0.8, 0.9)	0.0 (-0.1, 0.0)	3.1 (3.0, 3.2)	-0.1 (-0.1, 0.0)
2030	W	Hispanic	54.6 (54.4, 54.7)	0.1 (0.0, 0.2)	2.9 (2.9, 3.0)	0.0 (-0.1, 0.0)	1.6 (1.5, 1.6)	0.0 (-0.1, 0.0)	4.5 (4.4, 4.6)	-0.1 (-0.2, 0.0)
2030	W	Non-Hispanic White	63.7 (63.6, 63.9)	0.1 (0.0, 0.3)	5.3 (5.3, 5.4)	0.0 (-0.1, 0.0)	3.8 (3.6, 3.9)	-0.1 (-0.2, 0.0)	9.1 (8.9, 9.3)	-0.1 (-0.3, -0.1)
Scenario 2: Screening expansion by +20M, brief intervention expansion by +4M										
2030	M	Non-Hispanic Black	54.4 (54.3, 54.5)	0.6 (0.4, 0.7)	2.5 (2.4, 2.6)	-0.5 (-0.6, -0.4)	1.3 (1.2, 1.3)	-0.2 (-0.2, -0.2)	3.7 (3.6, 3.9)	-0.7 (-0.8, -0.6)
2030	M	Hispanic	59.2 (59.0, 59.3)	0.7 (0.5, 0.8)	3.6 (3.5, 3.7)	-0.5 (-0.6, -0.4)	2.7 (2.7, 2.8)	-0.3 (-0.3, -0.2)	6.3 (6.2, 6.5)	-0.8 (-0.9, -0.7)
2030	M	Non-Hispanic White	66.5 (66.3, 66.7)	1.5 (1.3, 1.6)	5.6 (5.5, 5.7)	-0.9 (-1.0, -0.8)	4.9 (4.8, 5.1)	-0.8 (-0.9, -0.7)	10.5 (10.3, 10.7)	-1.7 (-1.8, -1.6)
2030	W	Non-Hispanic Black	50.8 (50.6, 51.0)	0.4 (0.3, 0.5)	1.9 (1.9, 2.0)	-0.3 (-0.4, -0.3)	0.7 (0.7, 0.8)	-0.2 (-0.2, -0.1)	2.7 (2.5, 2.8)	-0.5 (-0.6, -0.4)
2030	W	Hispanic	55.0 (54.8, 55.1)	0.5 (0.3, 0.6)	2.6 (2.5, 2.7)	-0.4 (-0.5, -0.2)	1.4 (1.3, 1.5)	-0.2 (-0.2, -0.2)	4.0 (3.9, 4.2)	-0.6 (-0.7, -0.4)

© 2026 Lemp, JM et al. JAMA Health Forum.

			Category I alcohol use		Category II alcohol use		Category III alcohol use		Hazardous alcohol use (combined Category II and III)	
**Year**	**Sex**	**Education**	**Prevalence in % (CrI)**	**Change in PP (CrI)**	**Prevalence in % (CrI)**	**Change in PP (CrI)**	**Prevalence in % (CrI)**	**Change in PP (CrI)**	**Prevalence in % (CrI)**	**Change in PP (CrI)**
2030	W	Non-Hispanic White	64.7 (64.5, 65.0)	1.1 (0.9, 1.3)	4.8 (4.7, 5.0)	-0.5 (-0.7, -0.4)	3.1 (2.9, 3.3)	-0.8 (-0.8, -0.7)	7.9 (7.6, 8.2)	-1.3 (-1.5, -1.1)
Scenario 3: Screening expansion by +20M, brief intervention expansion by +8M										
2030	M	Non-Hispanic Black	54.8 (54.6, 55.0)	1.0 (0.9, 1.1)	2.0 (1.9, 2.1)	-0.9 (-1.0, -0.8)	1.1 (1.1, 1.2)	-0.3 (-0.4, -0.3)	3.2 (3.1, 3.3)	-1.3 (-1.4, -1.1)
2030	M	Hispanic	59.8 (59.5, 59.9)	1.3 (1.0, 1.4)	3.1 (3.0, 3.3)	-1.0 (-1.1, -0.8)	2.5 (2.4, 2.6)	-0.5 (-0.5, -0.4)	5.6 (5.4, 5.9)	-1.5 (-1.7, -1.2)
2030	M	Non-Hispanic White	67.8 (67.4, 68.0)	2.8 (2.4, 2.9)	4.6 (4.5, 4.8)	-1.8 (-1.9, -1.7)	4.4 (4.2, 4.6)	-1.4 (-1.6, -1.1)	9.0 (8.8, 9.3)	-3.2 (-3.4, -2.8)
2030	W	Non-Hispanic Black	51.2 (51.1, 51.4)	0.8 (0.6, 1.0)	1.5 (1.4, 1.6)	-0.8 (-0.9, -0.6)	0.6 (0.5, 0.7)	-0.3 (-0.3, -0.3)	2.1 (1.9, 2.3)	-1.1 (-1.2, -0.9)
2030	W	Hispanic	55.5 (55.3, 55.7)	1.0 (0.9, 1.2)	2.1 (1.9, 2.3)	-0.9 (-1.0, -0.7)	1.2 (1.1, 1.3)	-0.4 (-0.4, -0.4)	3.3 (3.0, 3.5)	-1.3 (-1.5, -1.1)
2030	W	Non-Hispanic White	66.0 (65.7, 66.4)	2.4 (2.1, 2.7)	3.9 (3.6, 4.2)	-1.4 (-1.8, -1.1)	2.4 (2.2, 2.6)	-1.4 (-1.5, -1.4)	6.3 (5.8, 6.7)	-2.9 (-3.3, -2.5)
Scenario 4: Universal screening and brief intervention										
2030	M	Non-Hispanic Black	55.2 (55.0, 55.4)	1.4 (1.2, 1.6)	1.7 (1.5, 1.9)	-1.3 (-1.4, -1.1)	1.0 (0.9, 1.1)	-0.5 (-0.5, -0.4)	2.7 (2.5, 2.9)	-1.8 (-1.9, -1.5)
2030	M	Hispanic	60.3 (59.9, 60.5)	1.7 (1.4, 2.0)	2.7 (2.5, 3.0)	-1.5 (-1.7, -1.1)	2.3 (2.2, 2.4)	-0.7 (-0.8, -0.6)	5.0 (4.7, 5.4)	-2.1 (-2.5, -1.7)
2030	M	Non-Hispanic White	68.6 (68.0, 69.1)	3.6 (3.0, 4.0)	4.0 (3.8, 4.4)	-2.4 (-2.7, -2.0)	4.0 (3.7, 4.3)	-1.8 (-2.0, -1.5)	8.0 (7.5, 8.7)	-4.3 (-4.7, -3.5)
2030	W	Non-Hispanic Black	51.6 (51.4, 51.7)	1.1 (1.0, 1.3)	1.1 (1.1, 1.3)	-1.1 (-1.2, -1.0)	0.5 (0.4, 0.6)	-0.4 (-0.4, -0.4)	1.6 (1.5, 1.8)	-1.6 (-1.7, -1.4)
2030	W	Hispanic	56.0 (55.7, 56.2)	1.5 (1.3, 1.7)	1.6 (1.5, 1.8)	-1.4 (-1.4, -1.2)	1.1 (1.0, 1.2)	-0.6 (-0.6, -0.5)	2.7 (2.5, 3.0)	-1.9 (-2.0, -1.7)
2030	W	Non-Hispanic White	67.0 (66.6, 67.3)	3.4 (3.1, 3.6)	3.1 (3.0, 3.3)	-2.2 (-2.3, -2.0)	1.9 (1.8, 2.2)	-1.9 (-2.0, -1.7)	5.1 (4.8, 5.5)	-4.2 (-4.4, -3.8)

Note: CrI: Credible interval, 2.5^{th} and 97.5^{th} rank of the simulated expansion effect across 70 model iterations, M: Men, W: Women, PP: percentage points. Standard brief intervention effect: M = -2.86, SE = 0.58.

© 2026 Lemp, JM et al. JAMA Health Forum.

eTable 3. Simulated prevalence of heavy episodic drinking by sex and education (standard effect).

		High school degree or less			Some college			College degree or more		
**Year**	**Sex**	**Prevalence in % (CrI)**	**Change in PP (CrI)**	**Relative change in % (CrI)**	**Prevalence in % (CrI)**	**Change in PP (CrI)**	**Relative change in % (CrI)**	**Prevalence in % (CrI)**	**Change in PP (CrI)**	**Relative change in % (CrI)**
Reference scenario (no expansion)										
2000	M	19.3 (19.3, 19.3)	.	.	28.5 (28.5, 28.5)	.	.	16.4 (16.4, 16.4)	.	.
2000	M	5.7 (5.7, 5.7)	.	.	13.7 (13.7, 13.7)	.	.	8.3 (8.3, 8.3)	.	.
2030	M	16.9 (16.7, 17.0)	.	.	21.3 (21.1, 21.5)	.	.	16.2 (16.1, 16.3)	.	.
2030	W	5.8 (5.7, 5.9)	.	.	9.1 (8.9, 9.2)	.	.	7.8 (7.7, 7.9)	.	.
Scenario 1: Screening expansion by +20M										
2030	M	16.8 (16.7, 17.0)	0.0 (-0.1, 0.0)	-0.3 (-0.5, 0.0)	21.3 (21.0, 21.5)	0.0 (-0.1, 0.0)	-0.1 (-0.3, 0.1)	16.2 (16.0, 16.3)	-0.1 (-0.1, 0.0)	-0.4 (-0.6, -0.1)
2030	W	5.8 (5.6, 5.9)	0.0 (-0.1, 0.0)	-0.6 (-1.3, -0.1)	9.1 (8.9, 9.2)	0.0 (0.0, 0.0)	-0.1 (-0.5, 0.2)	7.8 (7.7, 7.8)	0.0 (-0.1, 0.0)	-0.3 (-0.7, 0.0)
Scenario 2: Screening expansion by +20M, brief intervention expansion by +4M										
2030	M	16.6 (16.4, 16.8)	-0.3 (-0.4, -0.2)	-1.7 (-2.1, -1.2)	21.1 (20.9, 21.3)	-0.2 (-0.3, -0.2)	-1.0 (-1.3, -0.7)	15.6 (15.4, 15.7)	-0.6 (-0.7, -0.5)	-3.8 (-4.4, -3.2)
2030	W	5.6 (5.4, 5.7)	-0.2 (-0.3, -0.2)	-3.9 (-4.8, -2.7)	8.9 (8.8, 9.1)	-0.1 (-0.2, -0.1)	-1.6 (-2.3, -1.1)	7.4 (7.3, 7.5)	-0.4 (-0.5, -0.3)	-5.7 (-6.7, -4.4)
Scenario 3: Screening expansion by +20M, brief intervention expansion by +8M										
2030	M	16.3 (16.1, 16.5)	-0.6 (-0.7, -0.5)	-3.6 (-3.9, -3.2)	21.0 (20.7, 21.2)	-0.4 (-0.4, -0.3)	-1.7 (-2.0, -1.3)	15.2 (15.0, 15.3)	-1.0 (-1.2, -0.9)	-6.4 (-7.3, -5.4)
2030	W	5.3 (5.1, 5.4)	-0.5 (-0.6, -0.4)	-8.8 (-9.9, -7.2)	8.7 (8.6, 8.9)	-0.4 (-0.4, -0.3)	-4.0 (-4.7, -3.2)	6.8 (6.7, 6.9)	-1.0 (-1.1, -0.9)	-13.3 (-14.6, -11.6)
Scenario 4: Universal screening and brief intervention										
2030	M	15.9 (15.7, 16.1)	-1.0 (-1.1, -0.9)	-5.8 (-6.5, -5.1)	20.9 (20.6, 21.1)	-0.4 (-0.5, -0.3)	-2.0 (-2.5, -1.5)	15.0 (14.8, 15.2)	-1.2 (-1.4, -1.0)	-7.6 (-8.5, -6.3)
2030	W	4.9 (4.8, 5.1)	-0.9 (-1.0, -0.8)	-15.3 (-16.4, -13.9)	8.5 (8.4, 8.6)	-0.5 (-0.6, -0.5)	-6.0 (-6.6, -5.2)	6.4 (6.3, 6.5)	-1.4 (-1.5, -1.3)	-18.2 (-19.5, -16.0)

Note: CrI: Credible interval, 2.5th and 97.5th rank of the simulated expansion effect across 70 model iterations, M: Men, W: Women, ≤High school: High school degree or less, ≥College: College degree or more. Standard brief intervention effect: M = -2.86, SE = 0.58.

© 2026 Lemp, JM et al. JAMA Health Forum.

eTable 4. Simulated prevalence of heavy episodic drinking by sex and race and ethnicity (standard effect).

		Non-Hispanic Black			Hispanic			Non-Hispanic White		
Year	Sex	Prevalence in % (CrI)	Change in PP (CrI)	Relative change in % (CrI)	Prevalence in % (CrI)	Change in PP (CrI)	Relative change in % (CrI)	Prevalence in % (CrI)	Change in PP (CrI)	Relative change in % (CrI)
Reference scenario (no expansion)										
2000	M	13.6 (13.6, 13.6)	.	.	25.3 (25.3, 25.3)	.	.	20.8 (20.8, 20.8)	.	.
2000	M	4.3 (4.3, 4.3)	.	.	5.0 (5.0, 5.0)	.	.	9.2 (9.2, 9.2)	.	.
2030	M	8.2 (8.2, 8.3)	.	.	16.5 (16.4, 16.6)	.	.	20.6 (20.5, 20.8)	.	.
2030	W	3.1 (3.0, 3.1)	.	.	4.9 (4.9, 5.0)	.	.	9.3 (9.2, 9.4)	.	.
Scenario 1: Screening expansion by +20M										
2030	M	8.2 (8.2, 8.3)	0.0 (-0.1, 0.0)	-0.3 (-1.0, 0.2)	16.5 (16.4, 16.6)	0.0 (-0.1, 0.0)	-0.2 (-0.5, 0.2)	20.6 (20.4, 20.7)	-0.1 (-0.1, 0.0)	-0.3 (-0.5, -0.1)
2030	W	3.1 (3.0, 3.1)	0.0 (0.0, 0.0)	-0.5 (-1.3, 0.3)	4.9 (4.8, 4.9)	0.0 (-0.1, 0.0)	-0.3 (-1.0, 0.4)	9.3 (9.2, 9.4)	0.0 (-0.1, 0.0)	-0.3 (-0.7, 0.1)
Scenario 2: Screening expansion by +20M, brief intervention expansion by +4M										
2030	M	8.1 (8.0, 8.2)	-0.2 (-0.2, -0.1)	-2.1 (-2.7, -1.4)	16.3 (16.2, 16.4)	-0.2 (-0.2, -0.1)	-1.2 (-1.5, -0.8)	20.1 (20.0, 20.3)	-0.5 (-0.5, -0.4)	-2.3 (-2.6, -2.1)
2030	W	2.9 (2.9, 3.0)	-0.1 (-0.2, -0.1)	-4.6 (-5.6, -3.2)	4.7 (4.7, 4.8)	-0.2 (-0.2, -0.1)	-3.6 (-4.3, -2.6)	9.0 (8.8, 9.1)	-0.4 (-0.4, -0.3)	-3.8 (-4.2, -3.2)
Scenario 3: Screening expansion by +20M, brief intervention expansion by +8M										
2030	M	7.9 (7.9, 8.0)	-0.3 (-0.4, -0.2)	-3.8 (-4.4, -2.9)	16.2 (16.1, 16.2)	-0.4 (-0.4, -0.3)	-2.2 (-2.6, -1.8)	19.7 (19.6, 19.9)	-0.9 (-0.9, -0.8)	-4.3 (-4.6, -3.9)
2030	W	2.7 (2.7, 2.8)	-0.3 (-0.4, -0.3)	-10.7 (-11.6, -9.4)	4.5 (4.4, 4.6)	-0.4 (-0.5, -0.4)	-8.8 (-10.0, -7.4)	8.5 (8.3, 8.6)	-0.8 (-0.9, -0.7)	-8.9 (-9.8, -7.9)
Scenario 4: Universal screening and brief intervention										
2030	M	7.8 (7.7, 7.9)	-0.5 (-0.5, -0.4)	-5.5 (-6.1, -4.6)	16.0 (15.9, 16.1)	-0.5 (-0.6, -0.4)	-3.2 (-3.7, -2.7)	19.4 (19.3, 19.6)	-1.2 (-1.3, -1.0)	-5.7 (-6.3, -5.0)
2030	W	2.6 (2.5, 2.6)	-0.5 (-0.6, -0.5)	-16.7 (-18.0, -15.3)	4.2 (4.2, 4.3)	-0.7 (-0.7, -0.6)	-13.6 (-14.5, -12.4)	8.1 (8.0, 8.2)	-1.2 (-1.3, -1.1)	-13.2 (-14.0, -11.7)

Note: CrI: Credible interval, 2.5th and 97.5th rank of the simulated expansion effect across 70 model iterations, M: Men, W: Women. Standard brief intervention effect: M = -2.86, SE = 0.58.

© 2026 Lemp, JM et al. JAMA Health Forum.

eTable 5. Simulated combined YLL per 100,000 by sex.

Year	Sex	Scenario	YLL per 100k (CrI)	Absolute change in YLL (CrI)	Relative change in % (CrI)
Main analysis: Standard effect size – key (5) alcohol-related causes					
2000	M	Reference	2627 (2624, 2629)	.	.
2000	W	Reference	768 (766, 769)	.	.
2030	M	Reference	1823 (1805, 1840)	.	.
2030	W	Reference	774 (765, 784)	.	.
2030	M	Scenario 1	1815 (1800, 1833)	-8.0 (-19.6, 2.4)	-0.4 (-1.1, 0.1)
2030	W	Scenario 1	772 (758, 783)	-1.9 (-8.8, 4.9)	-0.2 (-1.1, 0.6)
2030	M	Scenario 2	1794 (1772, 1810)	-29.0 (-42.9, -14.0)	-1.6 (-2.4, -0.8)
2030	W	Scenario 2	759 (747, 769)	-15.1 (-22.9, -5.8)	-2.0 (-3.0, -0.7)
2030	M	Scenario 3	1772 (1750, 1790)	-51.3 (-73.2, -32.8)	-2.8 (-4.0, -1.8)
2030	W	Scenario 3	740 (720, 757)	-34.1 (-54.5, -16.1)	-4.4 (-7.1, -2.1)
2030	M	Scenario 4	1754 (1721, 1775)	-68.9 (-102.2, -44.8)	-3.8 (-5.6, -2.5)
2030	W	Scenario 4	722 (697, 741)	-52.0 (-75.0, -30.2)	-6.7 (-9.7, -3.9)
Sensitivity analysis 1: Maximum effect size					
2030	M	Scenario 1	1815 (1796, 1833)	-8.3 (-20.4, 5.3)	-0.5 (-1.1, 0.3)
2030	W	Scenario 1	771 (761, 782)	-1.8 (-8.1, 6.0)	-0.2 (-1.0, 0.8)
2030	M	Scenario 2	1785 (1767, 1805)	-38.4 (-57.3, -18.3)	-2.1 (-3.1, -1.0)
2030	W	Scenario 2	753 (740, 765)	-19.9 (-35.0, -9.3)	-2.6 (-4.5, -1.2)
2030	M	Scenario 3	1756 (1728, 1776)	-67.8 (-98.0, -46.7)	-3.7 (-5.4, -2.6)
2030	W	Scenario 3	727 (702, 749)	-46.1 (-70.6, -25.2)	-6.0 (-9.1, -3.3)
2030	M	Scenario 4	1732 (1691, 1762)	-91.4 (-131.0, -63.6)	-5.0 (-7.2, -3.5)
2030	W	Scenario 4	707 (673, 733)	-66.2 (-99.7, -38.7)	-8.6 (-12.9, -5.0)
	Sensitivity analysis 1: Minimum effect size				
2030	M	Scenario 1	1818 (1798, 1835)	-6.0 (-16.7, 3.3)	-0.3 (-0.9, 0.2)
2030	W	Scenario 1	773 (761, 782)	-0.6 (-9.5, 6.7)	-0.1 (-1.2, 0.9)
2030	M	Scenario 2	1804 (1786, 1824)	-19.8 (-28.8, -8.5)	-1.1 (-1.6, -0.5)
2030	W	Scenario 2	765 (754, 776)	-8.7 (-19.5, 0.9)	-1.1 (-2.5, 0.1)
2030	M	Scenario 3	1793 (1769, 1813)	-31.3 (-45.2, -17.9)	-1.7 (-2.5, -1.0)
2030	W	Scenario 3	753 (739, 766)	-20.8 (-34.5, -9.4)	-2.7 (-4.4, -1.2)
2030	M	Scenario 4	1779 (1756, 1795)	-44.5 (-65.7, -27.3)	-2.4 (-3.6, -1.5)
2030	W	Scenario 4	739 (720, 756)	-34.5 (-53.4, -19.2)	-4.5 (-6.9, -2.5)
Sensitivity analysis 2: Inclusion of alcohol use category I					
2030	M	Scenario 4	1759 (1731, 1783)	-68.2 (-98.2, -49.7)	-3.7 (-5.4, -2.7)
2030	W	Scenario 4	723 (696, 745)	-53.9 (-78.5, -32.3)	-6.9 (-10.1, -4.2)
Main analysis: Standard effect size – all (9) alcohol-related causes					
2000	M	Reference	4737 (4735, 4739)	.	.
2000	W	Reference	1669 (1667, 1671)	.	.
2030	M	Reference	3171 (3150, 3192)	.	.
2030	W	Reference	1388 (1377, 1400)	.	.
2030	M	Scenario 1	3163 (3139, 3181)	-8.4 (-21.6, 4.0)	-0.3 (-0.7, 0.1)
2030	W	Scenario 1	1386 (1372, 1399)	-2.6 (-11.2, 8.6)	-0.2 (-0.8, 0.6)
2030	M	Scenario 2	3140 (3118, 3161)	-30.9 (-44.3, -14.1)	-1.0 (-1.4, -0.4)
2030	W	Scenario 2	1371 (1357, 1386)	-17.7 (-27.9, -6.9)	-1.3 (-2.0, -0.5)
2030	M	Scenario 3	3116 (3089, 3136)	-54.9 (-78.6, -35.2)	-1.7 (-2.5, -1.1)
2030	W	Scenario 3	1349 (1326, 1369)	-39.3 (-58.9, -19.6)	-2.8 (-4.3, -1.4)
2030	M	Scenario 4	3099 (3063, 3127)	-72.6 (-106.5, -45.4)	-2.3 (-3.4, -1.4)
2030	W	Scenario 4	1329 (1302, 1356)	-59.0 (-87.5, -34.2)	-4.2 (-6.3, -2.5)

Note: CrI: Credible interval, 2.5^{th} and 97.5^{th} rank of the simulated expansion effect across 70 model iterations, M: Men, W: Women. Standard brief intervention effect: M = -2.86, SE = 0.58. Maximum brief intervention effect: M = -4.00, SE = 0.82. Minimum brief intervention effect: M = -1.72, SE = 0.35. Key (5) alcohol-related causes of death are: alcohol use disorder (AUD, including alcohol poisonings), liver disease and cirrhosis (including hepatitis C-related liver cirrhosis), motor-vehicle injuries, other unintentional injuries, and suicide. Additional causes of deaths are: Diabetes mellitus, hypertensive heart disease, ischemic heart disease, and ischemic stroke.

© 2026 Lemp, JM et al. JAMA Health Forum.

eTable 6. Simulated combined YLL per 100,000 by sex and education (standard effect).

		High school degree or less			Some college			College degree or more		
**Year**	**Sex**	**YLL per 100k (CrI)**	**Absolute change in YLL (CrI)**	**Relative change in % (CrI)**	**YLL per 100k (CrI)**	**Absolute change in YLL (CrI)**	**Relative change in % (CrI)**	**YLL per 100k (CrI)**	**Absolute change in YLL (CrI)**	**Relative change in % (CrI)**
Reference scenario (no expansion)										
2000	M	3530 (3526, 3535)	.	.	2187 (2183, 2190)	.	.	1100 (1098, 1102)	.	.
2000	M	911 (909, 912)	.	.	755 (752, 757)	.	.	447 (445, 449)	.	.
2030	M	2642 (2539, 2742)	.	.	1703 (1625, 1780)	.	.	825 (804, 855)	.	.
2030	W	1068 (1035, 1112)	.	.	852 (806, 890)	.	.	430 (414, 447)	.	.
Scenario 1: Screening expansion by +20M										
2030	M	2630 (2533, 2727)	-11.5 (-28.8, 5.1)	-0.4 (-1.1, 0.2)	1692 (1617, 1772)	-10.5 (-31.6, 11.8)	-0.6 (-1.9, 0.7)	825 (802, 856)	-0.9 (-13.6, 10.5)	-0.1 (-1.7, 1.3)
2030	W	1064 (1028, 1101)	-3.9 (-18.1, 9.7)	-0.4 (-1.7, 0.9)	851 (812, 886)	-1.4 (-15.5, 12.4)	-0.2 (-1.8, 1.5)	430 (416, 444)	-0.4 (-9.6, 8.2)	-0.1 (-2.2, 1.9)
Scenario 2: Screening expansion by +20M, brief intervention expansion by +4M										
2030	M	2605 (2504, 2715)	-37.1 (-61.8, -13.8)	-1.4 (-2.3, -0.5)	1674 (1599, 1753)	-28.7 (-51.8, -2.9)	-1.7 (-3.1, -0.2)	807 (785, 839)	-18.3 (-30.1, -5.5)	-2.2 (-3.6, -0.7)
2030	W	1049 (1012, 1087)	-18.8 (-37.0, 2.0)	-1.8 (-3.5, 0.2)	837 (794, 873)	-15.3 (-29.3, 1.6)	-1.8 (-3.4, 0.2)	419 (404, 437)	-11.3 (-20.9, -3.6)	-2.6 (-4.8, -0.8)
Scenario 3: Screening expansion by +20M, brief intervention expansion by +8M										
2030	M	2574 (2475, 2683)	-68.0 (-96.2, -40.3)	-2.6 (-3.6, -1.5)	1654 (1570, 1732)	-48.3 (-70.9, -20.6)	-2.8 (-4.2, -1.2)	794 (767, 826)	-31.3 (-48.3, -16.7)	-3.8 (-5.9, -2.1)
2030	W	1027 (988, 1066)	-40.9 (-61.6, -19.7)	-3.8 (-5.7, -1.9)	817 (774, 857)	-34.9 (-64.4, -14.1)	-4.1 (-7.4, -1.7)	403 (380, 425)	-26.9 (-43.1, -9.3)	-6.3 (-10.0, -2.2)
Scenario 4: Universal screening and brief intervention										
2030	M	2540 (2437, 2637)	-101.8 (-144.4, -68.0)	-3.9 (-5.4, -2.6)	1648 (1572, 1727)	-54.4 (-86.2, -26.1)	-3.2 (-4.9, -1.6)	788 (758, 820)	-37.3 (-59.3, -20.1)	-4.5 (-6.9, -2.4)
2030	W	1000 (953, 1044)	-67.7 (-94.4, -38.2)	-6.3 (-8.9, -3.6)	799 (756, 846)	-52.8 (-77.6, -30.2)	-6.2 (-9.2, -3.5)	394 (371, 419)	-36.3 (-55.9, -19.3)	-8.4 (-13.1, -4.4)

Note: CrI: Credible interval, 2.5th and 97.5th rank of the simulated expansion effect across 70 model iterations, M: Men, W: Women, ≤High school: High school degree or less, ≥College: College degree or more. Standard brief intervention effect: M = -2.86, SE = 0.58.

© 2026 Lemp, JM et al. JAMA Health Forum.

eTable 7. Simulated combined YLL per 100,000 by sex and race and ethnicity (standard effect).

		Non-Hispanic Black			Hispanic			Non-Hispanic White		
**Year**	**Sex**	**YLL per 100k**	**Absolute change in YLL**	**Relative change in % (CrI)**	**YLL per 100k**	**Absolute change in YLL**	**Relative change in % (CrI)**	**YLL per 100k**	**Absolute change in YLL**	**Relative change in % (CrI)**
Reference scenario (no expansion)										
2000	M	3133 (3125, 3142)	.	.	2863 (2854, 2870)	.	.	2548 (2546, 2550)	.	.
2000	M	852 (849, 855)	.	.	644 (641, 646)	.	.	775 (773, 776)	.	.
2030	M	2048 (1979, 2103)	.	.	1897 (1853, 1935)	.	.	1786 (1755, 1808)	.	.
2030	W	853 (828, 876)	.	.	659 (632, 693)	.	.	805 (795, 818)	.	.
Scenario 1: Screening expansion by +20M										
2030	M	2038 (1973, 2097)	-10.1 (-45.8, 24.9)	-0.5 (-2.2, 1.2)	1891 (1846, 1934)	-5.6 (-27.9, 17.6)	-0.3 (-1.5, 0.9)	1778 (1751, 1799)	-7.8 (-21.5, 9.0)	-0.4 (-1.2, 0.5)
2030	W	850 (819, 874)	-3.5 (-22.1, 11.6)	-0.4 (-2.6, 1.4)	659 (629, 687)	-0.7 (-17.1, 16.7)	-0.1 (-2.5, 2.6)	803 (790, 816)	-2.1 (-11.6, 9.1)	-0.3 (-1.4, 1.1)
Scenario 2: Screening expansion by +20M, brief intervention expansion by +4M										
2030	M	2028 (1960, 2086)	-20.1 (-60.8, 18.3)	-1.0 (-2.9, 0.9)	1876 (1829, 1918)	-21.2 (-40.8, 5.9)	-1.1 (-2.2, 0.3)	1752 (1729, 1774)	-34.0 (-50.7, -15.8)	-1.9 (-2.8, -0.9)
2030	W	843 (807, 873)	-10.5 (-35.9, 14.2)	-1.2 (-4.1, 1.7)	652 (626, 685)	-7.0 (-24.2, 6.4)	-1.0 (-3.6, 1.0)	786 (774, 802)	-19.7 (-32.8, -6.3)	-2.4 (-4.1, -0.8)
Scenario 3: Screening expansion by +20M, brief intervention expansion by +8M										
2030	M	2018 (1963, 2077)	-30.4 (-64.7, 2.9)	-1.5 (-3.2, 0.1)	1860 (1813, 1908)	-36.5 (-59.1, -12.2)	-1.9 (-3.1, -0.6)	1724 (1689, 1750)	-61.8 (-87.7, -36.6)	-3.5 (-4.9, -2.1)
2030	W	836 (803, 868)	-17.5 (-39.7, 3.6)	-2.1 (-4.6, 0.4)	643 (617, 673)	-16.2 (-37.2, 0.3)	-2.5 (-5.6, 0.1)	760 (734, 781)	-45.1 (-76.2, -21.8)	-5.6 (-9.4, -2.7)
Scenario 4: Universal screening and brief intervention										
2030	M	2014 (1957, 2075)	-34.4 (-70.8, 6.5)	-1.7 (-3.4, 0.3)	1846 (1801, 1892)	-51.3 (-83.9, -21.3)	-2.7 (-4.4, -1.1)	1701 (1656, 1732)	-84.5 (-120.5, -49.7)	-4.7 (-6.8, -2.8)
2030	W	828 (788, 862)	-25.5 (-48.5, -0.3)	-3.0 (-5.8, 0.0)	634 (603, 672)	-25.1 (-48.6, -5.2)	-3.8 (-7.4, -0.8)	736 (711, 762)	-69.0 (-96.6, -42.0)	-8.6 (-11.9, -5.3)

Note: CrI: Credible interval, 2.5th and 97.5th rank of the simulated expansion effect across 70 model iterations, M: Men, W: Women. Standard brief intervention effect: M = -2.86, SE = 0.58.

© 2026 Lemp, JM et al. JAMA Health Forum.

eTable 8. Simulated YLL per 100,000 by sex and cause (standard effect).

		Alcohol use disorder			Liver disease			Motor vehicle injuries		
Year	Sex	YLL per 100k	Absolute change in YLL	Relative change in % (CrI)	YLL per 100k	Absolute change in YLL	Relative change in % (CrI)	YLL per 100k	Absolute change in YLL	Relative change in % (CrI)
Reference scenario (no expansion)										
2000	M	140 (139, 142)	.	.	390 (389, 391)	.	.	923 (920, 925)	.	.
2000	W	31 (31, 32)	.	.	151 (151, 152)	.	.	318 (317, 319)	.	.
2030	M	205 (198, 211)	.	.	353 (346, 361)	.	.	444 (435, 453)	.	.
2030	W	89 (85, 94)	.	.	237 (230, 242)	.	.	170 (165, 175)	.	.
Scenario 1: Screening expansion by +20M										
2030	M	203 (196, 209)	-1.9 (-5.3, 1.5)	-0.9 (-2.6, 0.7)	353 (345, 361)	-0.1 (-4.9, 4.1)	0.0 (-1.4, 1.2)	441 (431, 450)	-2.6 (-6.7, 3.4)	-0.6 (-1.5, 0.8)
2030	W	88 (85, 94)	-0.5 (-3.1, 1.7)	-0.5 (-3.4, 2.0)	237 (230, 243)	0.5 (-2.9, 4.5)	0.2 (-1.2, 1.9)	168 (163, 173)	-1.1 (-4.2, 2.6)	-0.6 (-2.5, 1.6)
Scenario 2: Screening expansion by +20M, brief intervention expansion by +4M										
2030	M	194 (188, 200)	-10.4 (-13.1, -7.0)	-5.1 (-6.4, -3.4)	345 (335, 355)	-8.1 (-21.5, 0.7)	-2.3 (-6.1, 0.2)	440 (431, 449)	-4.4 (-11.0, 0.7)	-1.0 (-2.4, 0.2)
2030	W	86 (83, 91)	-2.8 (-5.2, -0.1)	-3.1 (-5.8, -0.1)	231 (222, 237)	-6.0 (-11.5, -1.6)	-2.5 (-4.9, -0.7)	168 (163, 173)	-1.3 (-5.0, 3.5)	-0.8 (-2.9, 2.1)
Scenario 3: Screening expansion by +20M, brief intervention expansion by +8M										
2030	M	186 (180, 193)	-18.7 (-23.3, -15.2)	-9.1 (-11.3, -7.4)	337 (316, 349)	-16.7 (-41.6, -2.4)	-4.7 (-11.6, -0.7)	437 (427, 446)	-6.5 (-11.5, -0.8)	-1.5 (-2.6, -0.2)
2030	W	83 (78, 88)	-5.7 (-8.6, -2.6)	-6.4 (-9.8, -3.0)	222 (212, 231)	-15.0 (-25.7, -6.4)	-6.3 (-10.7, -2.7)	167 (162, 172)	-2.4 (-5.6, 2.5)	-1.4 (-3.3, 1.5)
Scenario 4: Universal screening and brief intervention										
2030	M	178 (169, 185)	-26.6 (-33.8, -21.4)	-13.0 (-16.5, -10.6)	328 (300, 346)	-24.9 (-58.1, -4.9)	-7.0 (-16.2, -1.4)	437 (428, 446)	-6.8 (-13.1, -0.2)	-1.5 (-2.9, 0.0)
2030	W	80 (75, 86)	-8.9 (-12.5, -5.6)	-10.0 (-13.7, -6.3)	213 (198, 226)	-23.8 (-39.3, -11.2)	-10.1 (-16.4, -4.7)	167 (162, 171)	-2.9 (-7.3, 1.1)	-1.7 (-4.2, 0.7)
		Unintentional injuries			Suicide			Hypertensive heart disease		
Year	Sex	YLL per 100k	Absolute change in YLL	Relative change in % (CrI)	YLL per 100k	Absolute change in YLL	Relative change in % (CrI)	.	.	.
Reference scenario (no expansion)										
2000	M	473 (470, 476)	.	.	701 (699, 703)	.	.	111 (110, 112)	.	.
2000	W	118 (116, 119)	.	.	150 (149, 151)	.	.	56 (55, 56)	.	.
2030	M	253 (246, 259)	.	.	568 (558, 578)	.	.	223 (216, 229)	.	.
2030	W	107 (104, 111)	.	.	171 (166, 176)	.	.	105 (102, 108)	.	.

© 2026 Lemp, JM et al. JAMA Health Forum.

		Unintentional injuries			Suicide			Hypertensive heart disease		
Year	Sex	YLL per 100k	Absolute change in YLL	Relative change in % (CrI)	YLL per 100k	Absolute change in YLL	Relative change in % (CrI)	YLL per 100k	Absolute change in YLL	Relative change in % (CrI)
Scenario 1: Screening expansion by +20M										
2030	M	253 (247, 259)	-0.6 (-4.9, 3.2)	-0.2 (-1.9, 1.3)	566 (557, 576)	-2.6 (-9.9, 3.9)	-0.5 (-1.7, 0.7)	222 (216, 229)	-0.4 (-3.7, 3.2)	-0.2 (-1.6, 1.5)
2030	W	107 (103, 112)	-0.3 (-3.1, 2.7)	-0.3 (-2.8, 2.6)	171 (166, 174)	-0.6 (-3.6, 2.7)	-0.4 (-2.1, 1.6)	105 (101, 108)	-0.3 (-2.2, 2.2)	-0.3 (-2.1, 2.1)
Scenario 2: Screening expansion by +20M, brief intervention expansion by +4M										
2030	M	251 (244, 258)	-1.8 (-7.0, 2.2)	-0.7 (-2.8, 0.9)	564 (555, 575)	-4.3 (-10.0, 3.2)	-0.7 (-1.8, 0.6)	222 (217, 228)	-0.9 (-4.0, 3.1)	-0.4 (-1.8, 1.4)
2030	W	107 (104, 111)	-0.4 (-2.8, 2.9)	-0.3 (-2.6, 2.7)	167 (160, 172)	-4.7 (-11.6, 2.1)	-2.7 (-6.6, 1.3)	105 (101, 107)	-0.4 (-2.7, 1.9)	-0.4 (-2.6, 1.8)
Scenario 3: Screening expansion by +20M, brief intervention expansion by +8M										
2030	M	250 (242, 259)	-2.9 (-7.3, 0.5)	-1.2 (-2.9, 0.2)	562 (551, 573)	-6.6 (-14.2, 1.4)	-1.2 (-2.5, 0.2)	221 (216, 228)	-1.4 (-4.9, 2.8)	-0.6 (-2.2, 1.3)
2030	W	106 (103, 110)	-1.0 (-3.8, 1.2)	-0.9 (-3.5, 1.1)	161 (148, 170)	-10.0 (-23.5, -0.1)	-5.8 (-13.7, 0.0)	104 (100, 107)	-0.9 (-3.0, 1.6)	-0.8 (-2.9, 1.6)
Scenario 4: Universal screening and brief intervention										
2030	M	250 (244, 258)	-3.1 (-7.3, 1.2)	-1.2 (-2.9, 0.5)	561 (551, 572)	-7.6 (-15.0, -1.0)	-1.3 (-2.6, -0.2)	220 (215, 226)	-2.2 (-5.9, 0.9)	-1.0 (-2.6, 0.4)
2030	W	106 (103, 111)	-1.0 (-3.4, 1.5)	-0.9 (-3.1, 1.5)	156 (132, 168)	-15.4 (-37.0, -1.8)	-9.0 (-21.6, -1.1)	104 (101, 108)	-1.0 (-2.9, 1.1)	-0.9 (-2.7, 1.0)
		Diabetes mellitus			Ischemic heart disease			Ischemic stroke		
Year	Sex	YLL per 100k	Absolute change in YLL	Relative change in % (CrI)	YLL per 100k	Absolute change in YLL	Relative change in % (CrI)	YLL per 100k	Absolute change in YLL	Relative change in % (CrI)
2000	M	275 (273, 277)	.	.	1686 (1685, 1688)	.	.	38 (37, 40)	.	.
2000	W	205 (204, 207)	.	.	612 (611, 614)	.	.	28 (27, 29)	.	.
2030	M	314 (304, 321)	.	.	698 (687, 706)	.	.	114 (109, 120)	.	.
2030	W	168 (164, 172)	.	.	263 (259, 267)	.	.	78 (75, 82)	.	.
Scenario 1: Screening expansion by +20M										
2030	M	314 (305, 321)	0.0 (-6.0, 5.1)	0.0 (-1.9, 1.7)	697 (689, 706)	-0.3 (-6.7, 6.8)	0.0 (-0.9, 1.0)	114 (109, 121)	0.3 (-2.6, 2.9)	0.3 (-2.2, 2.6)
2030	W	168 (163, 171)	-0.6 (-3.6, 2.5)	-0.4 (-2.2, 1.5)	263 (260, 268)	0.1 (-4.0, 3.7)	0.0 (-1.5, 1.4)	79 (76, 82)	0.1 (-2.5, 2.3)	0.1 (-3.2, 3.1)
Scenario 2: Screening expansion by +20M, brief intervention expansion by +4M										
2030	M	314 (306, 321)	0.2 (-4.4, 5.3)	0.1 (-1.4, 1.7)	696 (689, 704)	-1.2 (-7.7, 6.8)	-0.2 (-1.1, 1.0)	114 (108, 120)	-0.1 (-2.8, 2.5)	0.0 (-2.4, 2.3)
2030	W	166 (160, 171)	-1.8 (-8.5, 3.1)	-1.0 (-5.0, 1.9)	263 (258, 268)	-0.4 (-4.7, 3.9)	-0.1 (-1.8, 1.5)	78 (75, 82)	0.0 (-2.6, 2.6)	0.0 (-3.3, 3.3)

© 2026 Lemp, JM et al. JAMA Health Forum.

		Diabetes mellitus			Ischemic heart disease			Ischemic stroke		
**Year**	**Sex**	**YLL per 100k**	**Absolute change in YLL**	**Relative change in % (CrI)**	**YLL per 100k**	**Absolute change in YLL**	**Relative change in % (CrI)**	**YLL per 100k**	**Absolute change in YLL**	**Relative change in % (CrI)**
Scenario 3: Screening expansion by +20M, brief intervention expansion by +8M										
2030	M	314 (305, 319)	0.0 (-4.6, 4.5)	0.0 (-1.5, 1.4)	696 (688, 702)	-2.0 (-10.7, 4.5)	-0.3 (-1.5, 0.6)	114 (108, 121)	-0.1 (-2.6, 2.5)	-0.1 (-2.3, 2.2)
2030	W	165 (151, 171)	-3.5 (-18.2, 3.5)	-2.0 (-10.6, 2.1)	263 (258, 268)	-0.4 (-4.2, 3.8)	-0.1 (-1.6, 1.5)	78 (75, 82)	-0.5 (-3.4, 2.4)	-0.7 (-4.1, 3.1)
Scenario 4: Universal screening and brief intervention										
2030	M	314 (306, 322)	0.3 (-3.6, 4.1)	0.1 (-1.1, 1.3)	696 (687, 704)	-1.9 (-10.2, 4.0)	-0.3 (-1.5, 0.6)	114 (108, 121)	0.2 (-3.1, 3.4)	0.2 (-2.7, 3.0)
2030	W	163 (143, 172)	-4.9 (-26.0, 4.9)	-2.9 (-15.1, 2.9)	262 (257, 267)	-0.8 (-5.9, 2.6)	-0.3 (-2.2, 1.0)	78 (75, 82)	-0.4 (-2.7, 2.2)	-0.4 (-3.4, 2.9)

Note: CrI: Credible interval, 2.5^{th} and 97.5^{th} rank of the simulated expansion effect across 70 model iterations, M: Men, W: Women. Standard brief intervention effect: M = -2.86, SE = 0.58.

© 2026 Lemp, JM et al. JAMA Health Forum.

eTable 9. Simulated YLL per 100,000 by sex comparing different liver disease aetiologies (standard effect).

		Liver disease and cirrhosis (combined)			Chronic pathway			Hepatitis C-related pathway		
Year	Sex	YLL per 100k	Absolute change in YLL	Relative change in % (CrI)	YLL per 100k	Absolute change in YLL	Relative change in % (CrI)	YLL per 100k	Absolute change in YLL	Relative change in % (CrI)
Reference scenario (no expansion)										
2000	M	390 (389, 391)	.	.	330 (328, 331)	.	.	60 (59, 61)	.	.
2000	W	151 (151, 152)	.	.	127 (127, 128)	.	.	24 (23, 25)	.	.
2030	M	353 (346, 361)	.	.	332 (325, 340)	.	.	21 (18, 23)	.	.
2030	W	237 (230, 242)	.	.	228 (221, 233)	.	.	8 (7, 9)	.	.
Scenario 1: Screening expansion by +20M										
2030	M	353 (345, 361)	-0.1 (-4.9, 4.1)	0.0 (-1.4, 1.2)	332 (326, 340)	-0.2 (-5.0, 4.3)	0.0 (-1.5, 1.3)	21 (18, 24)	0.0 (-1.4, 1.5)	0.2 (-6.7, 7.6)
2030	W	237 (230, 243)	0.5 (-2.9, 4.5)	0.2 (-1.2, 1.9)	229 (222, 235)	0.5 (-3.4, 4.1)	0.2 (-1.5, 1.8)	8 (7, 9)	0.0 (-0.7, 0.8)	0.4 (-8.5, 9.7)
Scenario 2: Screening expansion by +20M, brief intervention expansion by +4M										
2030	M	345 (335, 355)	-8.1 (-21.5, 0.7)	-2.3 (-6.1, 0.2)	324 (314, 334)	-8.0 (-21.2, 0.7)	-2.4 (-6.4, 0.2)	21 (18, 23)	-0.1 (-1.4, 1.2)	-0.6 (-6.3, 5.6)
2030	W	231 (222, 237)	-6.0 (-11.5, -1.6)	-2.5 (-4.9, -0.7)	222 (214, 228)	-6.0 (-11.8, -1.5)	-2.6 (-5.2, -0.6)	8 (7, 9)	0.0 (-0.9, 0.9)	0.4 (-10.8, 12.6)
Scenario 3: Screening expansion by +20M, brief intervention expansion by +8M										
2030	M	337 (316, 349)	-16.7 (-41.6, -2.4)	-4.7 (-11.6, -0.7)	316 (296, 328)	-16.4 (-41.1, -2.5)	-4.9 (-12.2, -0.7)	21 (18, 23)	-0.3 (-1.4, 0.8)	-1.2 (-6.5, 4.1)
2030	W	222 (212, 231)	-15.0 (-25.7, -6.4)	-6.3 (-10.7, -2.7)	214 (203, 222)	-15.0 (-25.6, -6.4)	-6.5 (-11.1, -2.8)	8 (7, 9)	-0.1 (-0.8, 0.7)	-0.6 (-9.1, 9.4)
Scenario 4: Universal screening and brief intervention										
2030	M	328 (300, 346)	-24.9 (-58.1, -4.9)	-7.0 (-16.2, -1.4)	308 (280, 325)	-24.4 (-58.1, -3.9)	-7.3 (-17.2, -1.2)	20 (18, 23)	-0.5 (-2.0, 0.7)	-2.4 (-9.7, 3.5)
2030	W	213 (198, 226)	-23.8 (-39.3, -11.2)	-10.1 (-16.4, -4.7)	205 (190, 218)	-23.7 (-39.4, -11.0)	-10.4 (-17.0, -4.8)	8 (7, 9)	-0.1 (-0.8, 0.6)	-1.6 (-8.8, 7.5)

Note: CrI: Credible interval, 2.5^{th} and 97.5^{th} rank of the simulated expansion effect across 70 model iterations, M: Men, W: Women. Standard brief intervention effect: M = -2.86, SE = 0.58.

© 2026 Lemp, JM et al. JAMA Health Forum.

eTable 10. Simulated YLL per 100,000 by sex, education, and cause (standard effect).

			Alcohol use disorder			Liver disease			Motor vehicle injuries		
Year	Sex	Education	YLL per 100k	Absolute change in YLL	Relative change in % (CrI)	YLL per 100k	Absolute change in YLL	Relative change in % (CrI)	YLL per 100k	Absolute change in YLL	Relative change in % (CrI)
Reference scenario (no expansion)											
2000	M	≤High school	197 (194, 199)	.	.	526 (525, 529)	.	.	1276 (1272, 1278)	.	.
2000	M	Some college	98 (96, 101)	.	.	306 (302, 308)	.	.	772 (768, 776)	.	.
2000	M	≥College	58 (56, 59)	.	.	173 (172, 175)	.	.	309 (305, 312)	.	.
2000	W	≤High school	40 (39, 40)	.	.	197 (197, 199)	.	.	374 (372, 376)	.	.
2000	W	Some college	26 (24, 27)	.	.	124 (123, 125)	.	.	336 (333, 341)	.	.
2000	W	≥College	17 (15, 18)	.	.	69 (69, 70)	.	.	168 (166, 171)	.	.
2030	M	≤High school	271 (255, 285)	.	.	499 (475, 517)	.	.	740 (707, 766)	.	.
2030	M	Some college	192 (177, 212)	.	.	331 (312, 355)	.	.	366 (348, 387)	.	.
2030	M	≥College	127 (117, 139)	.	.	176 (164, 186)	.	.	113 (105, 122)	.	.
2030	W	≤High school	109 (101, 119)	.	.	326 (311, 339)	.	.	281 (271, 294)	.	.
2030	W	Some college	104 (94, 118)	.	.	266 (250, 283)	.	.	171 (160, 181)	.	.
2030	W	≥College	58 (53, 67)	.	.	128 (119, 138)	.	.	61 (55, 67)	.	.
Scenario 1: Screening expansion by +20M											
2030	M	≤High school	268 (253, 282)	-2.7 (-8.8, 4.4)	-1.0 (-3.2, 1.7)	498 (476, 519)	-0.9 (-9.2, 8.3)	-0.2 (-1.8, 1.7)	736 (706, 761)	-3.9 (-17.4, 7.6)	-0.5 (-2.3, 1.0)
2030	M	Some college	190 (174, 209)	-1.7 (-9.9, 7.2)	-0.8 (-4.7, 3.9)	332 (314, 354)	0.3 (-9.0, 10.2)	0.1 (-2.8, 3.2)	363 (342, 386)	-2.9 (-13.6, 9.4)	-0.8 (-3.8, 2.5)
2030	M	≥College	126 (117, 138)	-1.2 (-6.7, 2.5)	-0.9 (-5.2, 2.0)	177 (166, 188)	0.5 (-4.2, 5.7)	0.3 (-2.4, 3.3)	112 (104, 122)	-0.5 (-5.8, 3.9)	-0.4 (-5.2, 3.6)
2030	W	≤High school	108 (100, 117)	-0.7 (-5.1, 4.2)	-0.6 (-4.4, 3.9)	326 (312, 339)	-0.1 (-9.0, 7.6)	0.0 (-2.7, 2.4)	279 (267, 293)	-1.6 (-9.2, 6.5)	-0.6 (-3.1, 2.3)
2030	W	Some college	103 (93, 116)	-0.4 (-5.1, 3.9)	-0.4 (-4.5, 3.7)	267 (251, 285)	1.6 (-5.5, 8.1)	0.6 (-2.0, 3.0)	170 (158, 182)	-1.4 (-9.7, 4.8)	-0.8 (-5.4, 2.9)
2030	W	≥College	58 (51, 66)	-0.3 (-3.9, 2.7)	-0.5 (-6.6, 4.7)	128 (120, 138)	0.4 (-3.0, 4.9)	0.3 (-2.3, 3.8)	61 (56, 67)	-0.3 (-4.3, 3.1)	-0.4 (-7.0, 5.2)
Scenario 2: Screening expansion by +20M, brief intervention expansion by +4M											
2030	M	≤High school	259 (244, 276)	-11.7 (-17.7, -5.5)	-4.3 (-6.7, -2.0)	488 (465, 509)	-10.9 (-30.4, 0.5)	-2.2 (-6.1, 0.1)	733 (703, 758)	-6.8 (-22.4, 3.3)	-0.9 (-3.0, 0.4)

© 2026 Lemp, JM et al. JAMA Health Forum.

			Alcohol use disorder			Liver disease			Motor vehicle injuries		
**Year**	**Sex**	**Education**	**YLL per 100k**	**Absolute change in YLL**	**Relative change in % (Crl)**	**Year**	**Sex**	**Education**	**YLL per 100k**	**Absolute change in YLL**	**Relative change in % (Crl)**
2030	M	Some college	182 (167, 197)	-10.0 (-17.2, -3.5)	-5.2 (-8.6, -1.9)	324 (304, 346)	-7.5 (-21.6, 10.1)	-2.2 (-6.5, 3.2)	362 (342, 386)	-3.4 (-12.3, 8.0)	-0.9 (-3.3, 2.3)
2030	M	≥College	118 (110, 130)	-8.9 (-14.2, -3.8)	-7.0 (-10.9, -3.2)	171 (160, 184)	-5.0 (-14.7, 3.7)	-2.9 (-8.3, 2.2)	111 (103, 122)	-1.9 (-6.2, 3.3)	-1.7 (-5.6, 3.0)
2030	W	≤High school	106 (97, 115)	-3.1 (-8.4, 2.8)	-2.8 (-7.3, 2.6)	318 (303, 333)	-7.9 (-16.9, 1.6)	-2.4 (-5.2, 0.5)	278 (267, 294)	-2.4 (-9.8, 5.8)	-0.8 (-3.5, 2.2)
2030	W	Some college	101 (91, 113)	-3.1 (-7.7, 0.7)	-2.9 (-7.2, 0.7)	260 (243, 276)	-6.0 (-15.7, 1.6)	-2.2 (-5.8, 0.6)	170 (159, 184)	-0.8 (-8.8, 6.4)	-0.5 (-4.9, 3.6)
2030	W	≥College	56 (50, 64)	-2.3 (-5.0, 0.7)	-3.8 (-8.6, 1.2)	124 (114, 133)	-4.2 (-9.7, 1.5)	-3.2 (-7.2, 1.2)	60 (55, 66)	-0.6 (-5.1, 1.8)	-0.9 (-7.6, 3.1)
Scenario 3: Screening expansion by +20M, brief intervention expansion by +8M											
2030	M	≤High school	248 (235, 263)	-22.8 (-30.2, -15.6)	-8.4 (-10.9, -6.0)	476 (447, 505)	-22.9 (-54.1, -2.8)	-4.6 (-10.9, -0.5)	730 (701, 756)	-10.1 (-20.2, 0.9)	-1.4 (-2.7, 0.1)
2030	M	Some college	175 (158, 192)	-16.8 (-25.0, -9.9)	-8.7 (-12.7, -5.3)	316 (292, 339)	-14.8 (-39.0, 5.0)	-4.4 (-11.6, 1.6)	360 (338, 383)	-5.7 (-17.3, 6.4)	-1.6 (-4.8, 1.7)
2030	M	≥College	113 (103, 125)	-14.8 (-21.0, -8.3)	-11.6 (-16.7, -6.8)	166 (151, 178)	-9.8 (-24.0, -0.2)	-5.6 (-13.6, -0.1)	110 (103, 119)	-2.3 (-8.0, 2.8)	-2.0 (-6.9, 2.5)
2030	W	≤High school	102 (95, 113)	-6.4 (-11.2, -1.4)	-5.9 (-10.5, -1.3)	307 (292, 327)	-19.5 (-34.8, -7.8)	-6.0 (-10.5, -2.4)	277 (264, 293)	-3.9 (-11.6, 5.5)	-1.4 (-4.0, 1.9)
2030	W	Some college	98 (87, 111)	-6.0 (-11.1, -0.1)	-5.7 (-10.4, -0.1)	251 (232, 269)	-15.2 (-30.3, -1.3)	-5.7 (-11.5, -0.5)	169 (156, 181)	-1.9 (-10.7, 6.1)	-1.1 (-6.2, 3.5)
2030	W	≥College	54 (48, 61)	-4.8 (-8.7, -1.4)	-8.1 (-14.9, -2.5)	118 (108, 128)	-10.6 (-19.4, -3.6)	-8.2 (-14.0, -2.8)	60 (54, 65)	-1.3 (-4.8, 1.5)	-2.0 (-7.6, 2.5)
Scenario 4: Universal screening and brief intervention											
2030	M	≤High school	234 (219, 248)	-36.7 (-49.4, -26.4)	-13.5 (-17.9, -10.3)	460 (415, 495)	-38.5 (-81.4, -11.8)	-7.7 (-16.5, -2.4)	728 (695, 761)	-11.8 (-22.6, 0.1)	-1.6 (-3.1, 0.0)
2030	M	Some college	170 (152, 185)	-21.7 (-31.3, -13.1)	-11.3 (-15.7, -7.0)	313 (282, 336)	-18.6 (-51.2, 1.8)	-5.6 (-15.4, 0.6)	362 (341, 383)	-4.3 (-15.6, 8.7)	-1.2 (-4.3, 2.4)
2030	M	≥College	110 (102, 121)	-17.3 (-22.6, -10.7)	-13.5 (-17.5, -9.2)	164 (149, 180)	-11.9 (-26.8, 0.5)	-6.8 (-15.2, 0.3)	111 (104, 119)	-2.1 (-7.8, 3.9)	-1.8 (-6.6, 3.5)
2030	W	≤High school	97 (89, 108)	-11.7 (-16.5, -5.7)	-10.7 (-15.2, -5.3)	293 (270, 315)	-33.3 (-53.4, -16.8)	-10.2 (-16.2, -5.1)	276 (262, 290)	-4.8 (-11.2, 3.4)	-1.7 (-4.0, 1.2)
2030	W	Some college	94 (84, 107)	-9.4 (-14.5, -3.6)	-9.0 (-13.3, -3.4)	243 (224, 260)	-23.3 (-39.6, -10.7)	-8.7 (-15.1, -4.1)	169 (158, 182)	-2.0 (-10.2, 4.7)	-1.2 (-5.9, 2.8)
2030	W	≥College	52 (47, 60)	-5.9 (-9.9, -2.6)	-10.1 (-17.0, -4.4)	113 (103, 126)	-15.1 (-25.9, -6.0)	-11.8 (-19.6, -4.7)	59 (54, 65)	-1.7 (-6.7, 1.7)	-2.7 (-9.9, 2.9)
			**Unintentional injuries**			**Suicide**			**Hypertensive heart disease**		
**Year**	**Sex**	**Education**	**YLL per 100k**	**Absolute change in YLL**	**Relative change in % (Crl)**	**YLL per 100k**	**Absolute change in YLL**	**Relative change in % (Crl)**	**YLL per 100k**	**Absolute change in YLL**	**Relative change in % (Crl)**
Reference scenario (no expansion)											
2000	M	≤High school	631 (627, 635)	.	.	901 (897, 903)	.	.	137 (135, 140)	.	.

© 2026 Lemp, JM et al. JAMA Health Forum.

Year	Sex	Education	Unintentional injuries			Suicide			Hypertensive heart disease		
			YLL per 100k	Absolute change in YLL	Relative change in % (CrI)	YLL per 100k	Absolute change in YLL	Relative change in % (CrI)	YLL per 100k	Absolute change in YLL	Relative change in % (CrI)
2000	M	Some college	374 (370, 378)	.	.	636 (634, 640)	.	.	97 (94, 100)	.	.
2000	M	≥College	223 (221, 227)	.	.	337 (335, 339)	.	.	67 (66, 69)	.	.
2000	W	≤High school	144 (142, 145)	.	.	156 (154, 158)	.	.	74 (73, 75)	.	.
2000	W	Some college	99 (97, 101)	.	.	170 (166, 173)	.	.	45 (44, 47)	.	.
2000	W	≥College	76 (73, 78)	.	.	117 (114, 119)	.	.	24 (22, 25)	.	.
2030	M	≤High school	367 (352, 385)	.	.	766 (734, 799)	.	.	311 (297, 325)	.	.
2030	M	Some college	222 (208, 238)	.	.	592 (557, 621)	.	.	209 (194, 225)	.	.
2030	M	≥College	127 (118, 136)	.	.	282 (269, 299)	.	.	115 (105, 123)	.	.
2030	W	≤High school	155 (148, 166)	.	.	197 (188, 210)	.	.	154 (147, 164)	.	.
2030	W	Some college	106 (98, 116)	.	.	205 (192, 222)	.	.	113 (103, 122)	.	.
2030	W	≥College	62 (57, 66)	.	.	121 (113, 131)	.	.	52 (47, 57)	.	.
Scenario 1: Screening expansion by +20M											
2030	M	≤High school	366 (350, 383)	-0.6 (-6.5, 6.4)	-0.2 (-1.8, 1.8)	762 (728, 798)	-3.4 (-14.8, 8.2)	-0.4 (-1.9, 1.1)	310 (293, 324)	-0.5 (-7.5, 7.1)	-0.2 (-2.4, 2.3)
2030	M	Some college	220 (204, 235)	-1.7 (-8.9, 6.8)	-0.8 (-3.9, 3.1)	588 (558, 621)	-4.5 (-20.1, 9.6)	-0.8 (-3.4, 1.6)	209 (193, 223)	-0.5 (-7.6, 7.3)	-0.2 (-3.6, 3.6)
2030	M	≥College	127 (118, 135)	0.2 (-5.6, 5.6)	0.2 (-4.5, 4.7)	282 (267, 299)	0.0 (-6.6, 8.8)	0.0 (-2.4, 3.1)	115 (106, 124)	-0.1 (-5.4, 5.3)	-0.1 (-4.7, 5.0)
2030	W	≤High school	155 (146, 166)	-0.4 (-5.2, 5.4)	-0.3 (-3.4, 3.6)	196 (187, 207)	-1.0 (-5.4, 5.5)	-0.5 (-2.5, 2.8)	154 (146, 164)	-0.3 (-5.3, 4.6)	-0.2 (-3.4, 3.0)
2030	W	Some college	106 (99, 115)	-0.5 (-5.9, 4.8)	-0.4 (-5.5, 4.8)	204 (194, 220)	-0.7 (-7.1, 7.2)	-0.3 (-3.2, 3.6)	113 (105, 121)	-0.5 (-6.0, 4.3)	-0.4 (-5.2, 3.9)
2030	W	≥College	62 (58, 67)	0.0 (-3.0, 3.0)	0.0 (-4.9, 5.0)	121 (113, 129)	-0.2 (-5.9, 4.3)	-0.1 (-4.8, 3.7)	52 (47, 57)	-0.2 (-3.2, 2.8)	-0.3 (-6.6, 5.4)
Scenario 2: Screening expansion by +20M, brief intervention expansion by +4M											
2030	M	≤High school	365 (346, 381)	-1.7 (-9.1, 6.0)	-0.5 (-2.5, 1.6)	760 (729, 793)	-5.9 (-18.1, 6.5)	-0.8 (-2.3, 0.9)	310 (295, 322)	-1.0 (-6.7, 4.9)	-0.3 (-2.2, 1.6)
2030	M	Some college	219 (203, 235)	-3.2 (-10.2, 4.0)	-1.4 (-4.5, 1.8)	587 (553, 622)	-4.7 (-18.5, 6.9)	-0.8 (-3.1, 1.2)	208 (194, 227)	-1.2 (-7.7, 7.3)	-0.5 (-3.8, 3.4)
2030	M	≥College	126 (117, 134)	-0.8 (-6.0, 4.3)	-0.6 (-4.6, 3.4)	281 (264, 296)	-1.7 (-9.5, 5.6)	-0.6 (-3.3, 2.0)	115 (105, 123)	-0.5 (-5.5, 5.0)	-0.4 (-4.6, 4.5)
2030	W	≤High school	154 (146, 164)	-0.8 (-6.9, 4.0)	-0.5 (-4.3, 2.7)	193 (181, 202)	-4.6 (-13.8, 6.7)	-2.3 (-6.6, 3.5)	153 (146, 162)	-0.6 (-4.6, 4.0)	-0.4 (-2.9, 2.6)

© 2026 Lemp, JM et al. JAMA Health Forum.

			Unintentional injuries			Suicide			Hypertensive heart disease		
Year	Sex	Education	YLL per 100k	Absolute change in YLL	Relative change in % (CrI)	YLL per 100k	Absolute change in YLL	Relative change in % (CrI)	YLL per 100k	Absolute change in YLL	Relative change in % (CrI)
2030	W	Some college	106 (98, 115)	-0.2 (-6.1, 5.7)	-0.1 (-5.3, 5.4)	200 (184, 215)	-5.2 (-13.5, 5.0)	-2.5 (-6.4, 2.6)	113 (103, 121)	-0.2 (-5.5, 4.7)	-0.1 (-4.6, 4.5)
2030	W	≥College	62 (57, 67)	-0.1 (-3.3, 4.4)	0.0 (-5.1, 7.8)	117 (109, 127)	-4.3 (-12.7, 2.4)	-3.5 (-10.3, 2.0)	51 (47, 57)	-0.5 (-3.8, 2.5)	-0.9 (-7.1, 4.9)
Scenario 3: Screening expansion by +20M, brief intervention expansion by +8M											
2030	M	≤High school	363 (346, 381)	-4.1 (-12.5, 4.1)	-1.1 (-3.4, 1.1)	758 (726, 796)	-8.2 (-22.3, 4.7)	-1.1 (-2.9, 0.6)	309 (292, 322)	-2.0 (-8.4, 4.2)	-0.6 (-2.8, 1.4)
2030	M	Some college	219 (204, 232)	-3.0 (-9.3, 4.3)	-1.4 (-4.2, 2.1)	584 (543, 616)	-8.0 (-21.6, 2.8)	-1.3 (-3.7, 0.5)	208 (194, 223)	-1.3 (-8.5, 5.4)	-0.6 (-4.1, 2.6)
2030	M	≥College	126 (118, 134)	-1.3 (-6.5, 5.2)	-1.0 (-5.0, 4.4)	279 (265, 297)	-3.1 (-10.4, 6.3)	-1.1 (-3.5, 2.2)	115 (106, 122)	-0.6 (-6.7, 4.2)	-0.5 (-5.7, 3.8)
2030	W	≤High school	154 (146, 164)	-1.3 (-5.9, 4.0)	-0.9 (-3.7, 2.6)	187 (171, 199)	-9.7 (-22.1, 3.0)	-4.9 (-11.0, 1.6)	153 (145, 162)	-1.2 (-5.7, 3.9)	-0.8 (-3.5, 2.6)
2030	W	Some college	106 (100, 112)	-0.7 (-5.8, 5.2)	-0.6 (-5.2, 5.1)	194 (172, 209)	-11.1 (-27.7, 4.0)	-5.4 (-13.8, 2.0)	112 (101, 120)	-0.9 (-5.8, 5.8)	-0.7 (-5.2, 5.2)
2030	W	≥College	61 (56, 65)	-0.8 (-5.8, 2.2)	-1.3 (-8.9, 3.7)	111 (99, 124)	-9.5 (-25.8, 0.6)	-7.8 (-20.0, 0.5)	51 (46, 56)	-0.6 (-3.5, 2.9)	-1.2 (-6.9, 5.8)
Scenario 4: Universal screening and brief intervention											
2030	M	≤High school	362 (346, 376)	-5.3 (-12.3, 0.4)	-1.4 (-3.4, 0.1)	756 (721, 789)	-9.5 (-23.8, 1.9)	-1.2 (-3.0, 0.2)	308 (294, 322)	-3.0 (-8.6, 4.9)	-1.0 (-2.8, 1.6)
2030	M	Some college	220 (204, 236)	-1.7 (-9.0, 5.6)	-0.8 (-4.0, 2.5)	584 (544, 616)	-8.0 (-20.8, 4.0)	-1.3 (-3.5, 0.7)	207 (193, 223)	-2.3 (-9.6, 5.1)	-1.1 (-4.4, 2.4)
2030	M	≥College	126 (118, 135)	-1.4 (-8.2, 5.0)	-1.1 (-6.2, 4.0)	278 (260, 296)	-4.6 (-15.8, 5.1)	-1.6 (-5.5, 1.8)	114 (106, 123)	-1.2 (-6.5, 3.7)	-1.0 (-5.7, 3.4)
2030	W	≤High school	153 (146, 164)	-1.6 (-8.4, 2.8)	-1.0 (-5.2, 1.8)	181 (154, 199)	-16.3 (-42.2, 0.8)	-8.2 (-20.0, 0.4)	152 (145, 162)	-1.6 (-7.2, 2.8)	-1.0 (-4.6, 1.8)
2030	W	Some college	106 (99, 113)	-0.7 (-4.8, 5.4)	-0.6 (-4.2, 5.1)	188 (160, 209)	-17.5 (-43.3, -1.7)	-8.5 (-21.3, -0.8)	112 (104, 121)	-0.7 (-6.2, 4.0)	-0.6 (-5.2, 3.6)
2030	W	≥College	61 (57, 67)	-0.7 (-3.9, 2.8)	-1.0 (-6.1, 4.4)	108 (89, 121)	-12.9 (-32.3, 1.0)	-10.6 (-26.6, 0.8)	51 (46, 57)	-0.6 (-4.2, 1.9)	-1.1 (-8.1, 4.0)
			**Diabetes mellitus**			**Ischemic heart disease**			**Ischemic stroke**		
**Year**	**Sex**	**Education**	**YLL per 100k**	**Absolute change in YLL**	**Relative change in % (CrI)**	**YLL per 100k**	**Absolute change in YLL**	**Relative change in % (CrI)**	**YLL per 100k**	**Absolute change in YLL**	**Relative change in % (CrI)**
2000	M	≤High school	358 (354, 363)	.	.	2165 (2162, 2168)	.	.	51 (49, 53)	.	.
2000	M	Some college	217 (212, 221)	.	.	1289 (1286, 1293)	.	.	27 (24, 29)	.	.
2000	M	≥College	147 (144, 151)	.	.	1014 (1010, 1018)	.	.	21 (18, 23)	.	.

© 2026 Lemp, JM et al. JAMA Health Forum.

			Diabetes mellitus			Ischemic heart disease			Ischemic stroke		
Year	Sex	Education	YLL per 100k	Absolute change in YLL	Relative change in % (Crl)	YLL per 100k	Absolute change in YLL	Relative change in % (Crl)	YLL per 100k	Absolute change in YLL	Relative change in % (Crl)
2000	W	≤High school	284 (282, 287)	.	.	860 (857, 862)	.	.	38 (36, 39)	.	.
2000	W	Some college	150 (146, 154)	.	.	424 (422, 427)	.	.	21 (19, 23)	.	.
2000	W	≥College	77 (75, 79)	.	.	223 (221, 226)	.	.	13 (11, 14)	.	.
2030	M	≤High school	443 (425, 461)	.	.	975 (941, 1004)	.	.	165 (155, 175)	.	.
2030	M	Some college	308 (292, 324)	.	.	646 (616, 676)	.	.	100 (84, 117)	.	.
2030	M	≥College	144 (137, 154)	.	.	369 (354, 386)	.	.	57 (49, 66)	.	.
2030	W	≤High school	255 (240, 270)	.	.	387 (373, 402)	.	.	121 (113, 130)	.	.
2030	W	Some college	187 (178, 196)	.	.	290 (274, 307)	.	.	80 (72, 88)	.	.
2030	W	≥College	70 (64, 76)	.	.	123 (116, 129)	.	.	37 (33, 42)	.	.
Scenario 1: Screening expansion by +20M											
2030	M	≤High school	443 (426, 462)	0.0 (-10.6, 7.3)	0.0 (-2.4, 1.7)	976 (946, 1011)	0.7 (-9.8, 14.0)	0.1 (-1.0, 1.4)	166 (154, 175)	0.3 (-4.6, 5.3)	0.2 (-2.7, 3.2)
2030	M	Some college	308 (289, 327)	0.1 (-8.6, 9.9)	0.1 (-2.7, 3.3)	644 (615, 680)	-1.5 (-14.0, 10.3)	-0.2 (-2.2, 1.7)	100 (86, 114)	0.4 (-5.3, 6.0)	0.5 (-5.0, 6.3)
2030	M	≥College	144 (134, 152)	-0.2 (-5.6, 5.9)	-0.1 (-3.9, 4.1)	368 (353, 382)	-0.7 (-10.2, 11.0)	-0.2 (-2.8, 3.1)	57 (49, 65)	0.4 (-4.7, 5.5)	0.8 (-7.6, 10.4)
2030	W	≤High school	254 (240, 265)	-1.5 (-7.1, 5.2)	-0.6 (-2.8, 2.0)	388 (372, 405)	0.8 (-6.6, 10.1)	0.2 (-1.7, 2.6)	120 (113, 129)	-0.4 (-5.8, 4.1)	-0.3 (-4.6, 3.5)
2030	W	Some college	187 (175, 197)	-0.4 (-7.7, 7.3)	-0.2 (-4.0, 3.9)	290 (272, 307)	-0.9 (-9.3, 8.6)	-0.3 (-3.2, 3.1)	80 (73, 88)	0.5 (-4.7, 5.8)	0.7 (-5.9, 7.7)
2030	W	≥College	70 (64, 75)	0.1 (-4.3, 3.6)	0.2 (-5.8, 5.1)	123 (115, 130)	0.1 (-6.1, 5.0)	0.1 (-5.0, 4.0)	37 (32, 42)	0.2 (-1.9, 2.5)	0.6 (-5.2, 6.7)
Scenario 2: Screening expansion by +20M, brief intervention expansion by +4M											
2030	M	≤High school	444 (425, 460)	0.3 (-8.0, 7.5)	0.1 (-1.7, 1.7)	975 (942, 1008)	-0.2 (-12.6, 10.8)	0.0 (-1.3, 1.1)	165 (155, 175)	-0.1 (-4.9, 4.9)	-0.1 (-2.9, 3.0)
2030	M	Some college	308 (292, 328)	0.5 (-8.1, 8.9)	0.2 (-2.6, 2.9)	643 (614, 675)	-2.3 (-13.9, 9.7)	-0.4 (-2.1, 1.5)	100 (86, 117)	0.0 (-6.2, 5.3)	0.0 (-6.3, 5.7)
2030	M	≥College	144 (137, 153)	-0.2 (-7.2, 7.1)	-0.1 (-4.9, 5.1)	368 (353, 387)	-1.4 (-12.7, 9.5)	-0.4 (-3.4, 2.6)	57 (50, 65)	0.0 (-4.5, 4.2)	0.1 (-7.4, 8.0)
2030	W	≤High school	252 (237, 265)	-3.3 (-15.8, 6.0)	-1.3 (-6.3, 2.4)	387 (374, 401)	-0.6 (-8.3, 8.2)	-0.1 (-2.1, 2.1)	121 (113, 128)	0.1 (-5.2, 5.0)	0.2 (-4.2, 4.1)
2030	W	Some college	186 (172, 199)	-1.4 (-11.8, 7.7)	-0.7 (-6.2, 4.1)	290 (273, 312)	-0.9 (-7.9, 8.3)	-0.3 (-2.8, 2.7)	79 (72, 90)	-0.5 (-4.7, 4.9)	-0.5 (-5.8, 6.7)
2030	W	≥College	69 (64, 75)	-0.6 (-5.2, 3.7)	-0.8 (-7.2, 5.4)	123 (116, 129)	0.2 (-5.1, 5.4)	0.2 (-4.2, 4.5)	37 (33, 42)	0.1 (-2.3, 3.0)	0.4 (-6.0, 8.0)

© 2026 Lemp, JM et al. JAMA Health Forum.

			Diabetes mellitus			Ischemic heart disease			Ischemic stroke		
**Year**	**Sex**	**Education**	**YLL per 100k**	**Absolute change in YLL**	**Relative change in % (Crl)**	**YLL per 100k**	**Absolute change in YLL**	**Relative change in % (Crl)**	**YLL per 100k**	**Absolute change in YLL**	**Relative change in % (Crl)**
Scenario 3: Screening expansion by +20M, brief intervention expansion by +8M											
2030	M	≤High school	444 (427, 461)	0.3 (-7.5, 7.6)	0.1 (-1.7, 1.7)	973 (938, 1000)	-1.5 (-16.8, 10.2)	-0.2 (-1.7, 1.0)	165 (155, 176)	-0.1 (-5.3, 4.4)	-0.1 (-3.1, 2.7)
2030	M	Some college	308 (289, 327)	-0.2 (-10.2, 11.5)	-0.1 (-3.2, 3.9)	643 (613, 672)	-2.1 (-15.9, 14.7)	-0.3 (-2.5, 2.3)	100 (84, 118)	-0.3 (-5.7, 4.9)	-0.3 (-5.5, 4.6)
2030	M	≥College	144 (137, 152)	-0.2 (-6.1, 4.5)	-0.1 (-4.1, 3.3)	367 (355, 380)	-2.5 (-11.9, 6.2)	-0.7 (-3.2, 1.7)	57 (49, 66)	0.0 (-3.2, 4.6)	0.0 (-5.5, 8.1)
2030	W	≤High school	249 (231, 264)	-5.8 (-26.5, 6.4)	-2.2 (-10.3, 2.6)	387 (373, 404)	-0.3 (-7.5, 8.3)	-0.1 (-1.9, 2.2)	120 (113, 128)	-1.0 (-6.6, 4.0)	-0.8 (-5.2, 3.5)
2030	W	Some college	184 (171, 196)	-3.3 (-16.4, 6.2)	-1.8 (-8.7, 3.3)	290 (274, 308)	-0.5 (-6.4, 7.6)	-0.2 (-2.2, 2.6)	79 (72, 88)	-0.4 (-5.8, 4.4)	-0.5 (-7.1, 5.4)
2030	W	≥College	68 (60, 76)	-1.3 (-11.7, 4.6)	-1.9 (-16.2, 6.8)	122 (115, 129)	-0.2 (-5.5, 4.8)	-0.2 (-4.4, 3.8)	36 (33, 42)	-0.2 (-2.8, 2.1)	-0.4 (-7.2, 5.8)
Scenario 4: Universal screening and brief intervention											
2030	M	≤High school	444 (424, 461)	0.6 (-7.2, 7.3)	0.1 (-1.6, 1.7)	973 (939, 1007)	-1.5 (-13.5, 9.1)	-0.2 (-1.4, 0.9)	166 (155, 176)	0.2 (-5.2, 4.6)	0.1 (-3.1, 2.8)
2030	M	Some college	308 (291, 329)	0.2 (-8.9, 9.2)	0.1 (-2.9, 3.1)	643 (615, 674)	-2.1 (-17.0, 9.8)	-0.3 (-2.7, 1.5)	100 (83, 117)	0.3 (-4.8, 7.1)	0.3 (-4.8, 7.6)
2030	M	≥College	144 (136, 154)	-0.1 (-7.5, 7.7)	0.0 (-5.1, 5.5)	367 (354, 382)	-2.4 (-13.1, 9.0)	-0.6 (-3.5, 2.4)	57 (49, 65)	0.2 (-4.0, 4.2)	0.4 (-6.7, 7.5)
2030	W	≤High school	247 (216, 266)	-7.9 (-37.6, 5.4)	-3.1 (-14.9, 2.1)	387 (370, 403)	-0.4 (-10.6, 8.2)	-0.1 (-2.8, 2.1)	120 (110, 129)	-0.8 (-5.2, 4.7)	-0.7 (-4.3, 4.0)
2030	W	Some college	183 (161, 197)	-4.5 (-26.2, 6.8)	-2.4 (-13.8, 3.7)	289 (273, 304)	-1.2 (-9.9, 6.8)	-0.4 (-3.4, 2.4)	80 (73, 88)	-0.2 (-4.9, 4.5)	-0.2 (-5.8, 5.9)
2030	W	≥College	68 (55, 74)	-2.2 (-14.4, 3.7)	-3.1 (-20.5, 5.7)	122 (115, 128)	-0.9 (-6.6, 3.7)	-0.7 (-5.3, 3.1)	37 (33, 42)	0.0 (-2.9, 2.7)	0.1 (-7.7, 7.8)

Note: CrI: Credible interval, 2.5^{th} and 97.5^{th} rank of the simulated expansion effect across 70 model iterations, M: Men, W: Women, ≤High school: High school degree or less, ≥College: College degree or more. Standard brief intervention effect: M = -2.86, SE = 0.58.

© 2026 Lemp, JM et al. JAMA Health Forum.

eTable 11. Simulated combined YLL per 100,000 by sex and education (sensitivity analysis 1, maximum effect).

		High school degree or less			Some college			College degree or more		
**Year**	**Sex**	**YLL per 100k (CrI)**	**Absolute change in YLL (CrI)**	**Relative change in % (CrI)**	**YLL per 100k (CrI)**	**Absolute change in YLL (CrI)**	**Relative change in % (CrI)**	**YLL per 100k (CrI)**	**Absolute change in YLL (CrI)**	**Relative change in % (CrI)**
Reference scenario (no expansion)										
2000	M	3540 (3536, 3544)	.	.	2188 (2185, 2191)	.	.	1098 (1096, 1100)	.	.
2000	M	912 (909, 914)	.	.	750 (746, 753)	.	.	450 (448, 453)	.	.
2030	M	2642 (2539, 2744)	.	.	1702 (1617, 1771)	.	.	826 (804, 858)	.	.
2030	W	1065 (1027, 1107)	.	.	853 (815, 889)	.	.	430 (414, 446)	.	.
Scenario 1: Screening expansion by +20M										
2030	M	2631 (2529, 2737)	-10.8 (-34.9, 14.7)	-0.4 (-1.3, 0.6)	1692 (1619, 1775)	-10.6 (-34.1, 14.7)	-0.6 (-2.0, 0.9)	823 (798, 855)	-2.9 (-14.3, 7.9)	-0.3 (-1.7, 1.0)
2030	W	1061 (1028, 1102)	-3.2 (-18.3, 12.2)	-0.3 (-1.7, 1.1)	850 (809, 888)	-3.0 (-18.6, 13.5)	-0.3 (-2.1, 1.6)	430 (413, 447)	0.3 (-6.8, 8.2)	0.1 (-1.6, 1.9)
Scenario 2: Screening expansion by +20M, brief intervention expansion by +4M										
2030	M	2594 (2490, 2702)	-48.5 (-72.6, -13.6)	-1.8 (-2.7, -0.5)	1666 (1588, 1749)	-36.3 (-65.3, -13.5)	-2.1 (-3.8, -0.8)	799 (779, 839)	-26.5 (-42.3, -12.4)	-3.2 (-5.1, -1.5)
2030	W	1041 (1006, 1080)	-23.1 (-43.9, -1.0)	-2.2 (-4.1, -0.1)	832 (790, 871)	-20.6 (-35.8, -5.6)	-2.4 (-4.1, -0.6)	414 (395, 434)	-16.4 (-29.9, -4.9)	-3.8 (-6.8, -1.1)
Scenario 3: Screening expansion by +20M, brief intervention expansion by +8M										
2030	M	2551 (2454, 2655)	-90.7 (-130.2, -59.9)	-3.4 (-5.0, -2.2)	1641 (1564, 1727)	-60.9 (-99.1, -31.2)	-3.6 (-5.9, -1.8)	783 (753, 816)	-42.8 (-63.6, -24.8)	-5.2 (-7.6, -3.1)
2030	W	1011 (972, 1052)	-54.0 (-86.2, -25.8)	-5.1 (-8.1, -2.4)	804 (762, 847)	-49.1 (-80.7, -27.5)	-5.8 (-9.3, -3.2)	394 (370, 418)	-36.1 (-56.5, -19.1)	-8.4 (-13.1, -4.5)
Scenario 4: Universal screening and brief intervention										
2030	M	2509 (2415, 2606)	-132.8 (-194.7, -87.1)	-5.0 (-7.4, -3.3)	1629 (1541, 1709)	-73.6 (-109.3, -42.2)	-4.3 (-6.3, -2.5)	775 (744, 807)	-51.0 (-77.4, -33.6)	-6.2 (-9.1, -4.1)
2030	W	978 (933, 1022)	-86.4 (-128.8, -48.3)	-8.1 (-12.1, -4.6)	786 (739, 830)	-67.0 (-106.2, -39.1)	-7.9 (-12.6, -4.6)	384 (357, 411)	-46.2 (-71.7, -27.2)	-10.7 (-16.4, -6.3)

Note: CI: Credible interval, 2.5th and 97.5th rank of the simulated expansion effect across 70 model iterations, M: Men, W: Women, ≤High school: High school degree or less, ≥College: College degree or more. Maximum brief intervention effect: M = -4.00, SE = 0.82.

© 2026 Lemp, JM et al. JAMA Health Forum.

eTable 12. Simulated combined YLL per 100,000 by sex and education (sensitivity analysis 1, minimum effect).

		High school degree or less			Some college			College degree or more		
Year	Sex	YLL per 100k (CrI)	Absolute change in YLL (CrI)	Relative change in % (CrI)	YLL per 100k (CrI)	Absolute change in YLL (CrI)	Relative change in % (CrI)	YLL per 100k (CrI)	Absolute change in YLL (CrI)	Relative change in % (CrI)
Reference scenario (no expansion)										
2000	M	3538 (3533, 3545)	.	.	2185 (2180, 2190)	.	.	1091 (1089, 1093)	.	.
2000	M	915 (913, 917)	.	.	752 (750, 755)	.	.	457 (456, 459)	.	.
2030	M	2643 (2532, 2742)	.	.	1703 (1613, 1781)	.	.	825 (800, 854)	.	.
2030	W	1068 (1033, 1108)	.	.	851 (805, 885)	.	.	430 (415, 445)	.	.
Scenario 1: Screening expansion by +20M										
2030	M	2633 (2530, 2726)	-10.1 (-32.0, 9.7)	-0.4 (-1.2, 0.4)	1697 (1614, 1777)	-6.2 (-33.0, 12.5)	-0.4 (-2.0, 0.7)	825 (802, 856)	-0.4 (-12.8, 10.5)	0.0 (-1.5, 1.3)
2030	W	1065 (1025, 1106)	-3.2 (-21.5, 8.8)	-0.3 (-2.0, 0.8)	851 (813, 887)	0.1 (-11.1, 12.2)	0.0 (-1.3, 1.5)	431 (414, 446)	1.3 (-8.0, 11.6)	0.3 (-1.8, 2.7)
Scenario 2: Screening expansion by +20M, brief intervention expansion by +4M										
2030	M	2616 (2517, 2719)	-26.3 (-44.4, -4.3)	-1.0 (-1.7, -0.2)	1683 (1602, 1760)	-20.0 (-48.0, -0.8)	-1.2 (-2.7, 0.0)	815 (792, 850)	-10.8 (-24.6, 1.8)	-1.3 (-2.9, 0.2)
2030	W	1057 (1018, 1099)	-11.5 (-29.3, 6.3)	-1.1 (-2.7, 0.6)	843 (810, 878)	-7.7 (-25.9, 6.9)	-0.9 (-3.0, 0.8)	423 (409, 440)	-6.7 (-17.3, 5.6)	-1.6 (-4.0, 1.3)
Scenario 3: Screening expansion by +20M, brief intervention expansion by +8M										
2030	M	2601 (2500, 2708)	-41.5 (-66.9, -16.0)	-1.6 (-2.6, -0.6)	1674 (1599, 1745)	-29.0 (-55.9, -0.7)	-1.7 (-3.2, 0.0)	806 (783, 841)	-19.4 (-39.8, -5.7)	-2.3 (-4.8, -0.7)
2030	W	1042 (1002, 1086)	-26.5 (-46.0, -6.6)	-2.5 (-4.3, -0.6)	830 (788, 867)	-20.8 (-39.2, -5.0)	-2.4 (-4.6, -0.6)	414 (396, 431)	-15.3 (-27.3, -3.6)	-3.6 (-6.3, -0.8)
Scenario 4: Universal screening and brief intervention										
2030	M	2578 (2479, 2681)	-64.8 (-91.6, -35.6)	-2.4 (-3.5, -1.4)	1666 (1587, 1748)	-37.2 (-65.6, -13.3)	-2.2 (-3.8, -0.8)	802 (775, 832)	-23.7 (-41.5, -11.3)	-2.9 (-5.0, -1.4)
2030	W	1023 (984, 1064)	-45.5 (-71.7, -22.0)	-4.3 (-6.7, -2.0)	817 (771, 862)	-34.4 (-55.1, -12.3)	-4.0 (-6.4, -1.5)	406 (389, 429)	-23.9 (-40.6, -7.7)	-5.6 (-9.3, -1.8)

Note: CrI: Credible interval, 2.5th and 97.5th rank of the simulated expansion effect across 70 model iterations, M: Men, W: Women, ≤High school: High school degree or less, ≥College: College degree or more. Minimum brief intervention effect: M = -1.72, SE = 0.35.

© 2026 Lemp, JM et al. JAMA Health Forum.

eTable 13. Simulated combined YLL per 100,000 by sex and race and ethnicity (sensitivity analysis 1, maximum effect).

		Non-Hispanic Black			Hispanic			Non-Hispanic White		
**Year**	**Sex**	**YLL per 100k (CrI)**	**Absolute change in YLL (CrI)**	**Relative change in % (CrI)**	**YLL per 100k (CrI)**	**Absolute change in YLL (CrI)**	**Relative change in % (CrI)**	**YLL per 100k (CrI)**	**Absolute change in YLL (CrI)**	**Relative change in % (CrI)**
Reference scenario (no expansion)										
2000	M	3160 (3153, 3170)	.	.	2909 (2896, 2916)	.	.	2544 (2541, 2546)	.	.
2000	M	855 (852, 858)	.	.	638 (635, 643)	.	.	773 (771, 774)	.	.
2030	M	2048 (1968, 2116)	.	.	1896 (1854, 1949)	.	.	1787 (1763, 1811)	.	.
2030	W	851 (816, 878)	.	.	661 (635, 692)	.	.	804 (794, 818)	.	.
Scenario 1: Screening expansion by +20M										
2030	M	2041 (1983, 2107)	-7.3 (-51.0, 29.1)	-0.3 (-2.4, 1.4)	1887 (1843, 1939)	-9.5 (-36.5, 16.8)	-0.5 (-1.9, 0.9)	1779 (1751, 1804)	-8.1 (-23.5, 7.2)	-0.5 (-1.3, 0.4)
2030	W	850 (816, 881)	-1.2 (-23.5, 22.9)	-0.1 (-2.7, 2.8)	659 (635, 687)	-1.4 (-18.2, 17.4)	-0.2 (-2.7, 2.7)	802 (790, 814)	-2.4 (-13.0, 7.9)	-0.3 (-1.6, 1.0)
Scenario 2: Screening expansion by +20M, brief intervention expansion by +4M										
2030	M	2025 (1968, 2089)	-23.7 (-70.0, 18.0)	-1.1 (-3.4, 0.9)	1867 (1823, 1911)	-29.5 (-64.1, 4.2)	-1.6 (-3.3, 0.2)	1741 (1717, 1764)	-45.8 (-67.9, -21.4)	-2.6 (-3.8, -1.2)
2030	W	844 (814, 873)	-6.9 (-26.4, 16.0)	-0.8 (-3.0, 1.9)	651 (626, 676)	-10.3 (-28.2, 6.8)	-1.5 (-4.1, 1.0)	777 (758, 795)	-26.9 (-46.4, -10.1)	-3.3 (-5.8, -1.2)
Scenario 3: Screening expansion by +20M, brief intervention expansion by +8M										
2030	M	2011 (1942, 2067)	-36.8 (-83.1, 13.8)	-1.8 (-4.0, 0.7)	1845 (1803, 1894)	-50.9 (-84.5, -19.0)	-2.7 (-4.4, -1.0)	1704 (1662, 1732)	-82.8 (-117.8, -53.7)	-4.6 (-6.6, -3.0)
2030	W	831 (802, 860)	-20.2 (-47.8, 3.1)	-2.4 (-5.5, 0.4)	637 (610, 667)	-23.8 (-42.6, -6.2)	-3.6 (-6.4, -1.0)	743 (712, 768)	-61.5 (-94.1, -32.7)	-7.6 (-11.7, -4.1)
Scenario 4: Universal screening and brief intervention										
2030	M	2002 (1945, 2062)	-45.9 (-82.0, -4.5)	-2.2 (-4.0, -0.2)	1826 (1766, 1880)	-69.9 (-105.8, -28.1)	-3.7 (-5.6, -1.5)	1675 (1622, 1710)	-111.7 (-157.5, -76.7)	-6.2 (-8.8, -4.3)
2030	W	821 (782, 853)	-30.0 (-56.3, -4.6)	-3.5 (-6.6, -0.6)	628 (599, 661)	-32.8 (-58.6, -12.1)	-5.0 (-8.8, -1.9)	716 (678, 750)	-88.1 (-129.3, -52.2)	-10.9 (-16.1, -6.4)

Note: CrI: Credible interval, 2.5th and 97.5th rank of the simulated expansion effect across 70 model iterations, M: Men, W: Women. Maximum brief intervention effect: M = -4.00, SE = 0.82.

© 2026 Lemp, JM et al. JAMA Health Forum.

eTable 14. Simulated combined YLL per 100,000 by sex and race and ethnicity (sensitivity analysis 1, minimum effect).

		Non-Hispanic Black			Hispanic			Non-Hispanic White		
**Year**	**Sex**	**YLL per 100k (CrI)**	**Absolute change in YLL (CrI)**	**Relative change in % (CrI)**	**YLL per 100k (CrI)**	**Absolute change in YLL (CrI)**	**Relative change in % (CrI)**	**YLL per 100k (CrI)**	**Absolute change in YLL (CrI)**	**Relative change in % (CrI)**
Reference scenario (no expansion)										
2000	M	3162 (3155, 3171)	.	.	2867 (2860, 2875)	.	.	2544 (2542, 2549)	.	.
2000	M	848 (843, 853)	.	.	654 (650, 657)	.	.	779 (778, 780)	.	.
2030	M	2049 (1993, 2111)	.	.	1896 (1858, 1940)	.	.	1787 (1762, 1808)	.	.
2030	W	853 (819, 881)	.	.	662 (636, 688)	.	.	804 (791, 816)	.	.
Scenario 1: Screening expansion by +20M										
2030	M	2044 (1978, 2103)	-4.9 (-39.5, 33.2)	-0.2 (-2.0, 1.6)	1891 (1841, 1933)	-5.3 (-36.9, 16.6)	-0.3 (-2.0, 0.9)	1781 (1753, 1805)	-6.3 (-19.7, 4.3)	-0.4 (-1.1, 0.2)
2030	W	852 (817, 878)	-1.2 (-30.3, 19.1)	-0.1 (-3.6, 2.3)	662 (635, 686)	-0.3 (-16.6, 18.0)	0.0 (-2.5, 2.7)	804 (789, 818)	-0.4 (-10.3, 10.1)	-0.1 (-1.3, 1.3)
Scenario 2: Screening expansion by +20M, brief intervention expansion by +4M										
2030	M	2037 (1975, 2092)	-12.6 (-48.2, 18.3)	-0.6 (-2.3, 0.9)	1880 (1835, 1929)	-15.7 (-41.8, 7.0)	-0.8 (-2.2, 0.4)	1764 (1736, 1785)	-23.2 (-37.1, -8.5)	-1.3 (-2.1, -0.5)
2030	W	848 (820, 878)	-4.8 (-22.8, 15.1)	-0.6 (-2.6, 1.8)	657 (630, 683)	-5.0 (-19.3, 10.1)	-0.8 (-2.8, 1.5)	793 (782, 809)	-11.1 (-26.4, 3.6)	-1.4 (-3.2, 0.4)
Scenario 3: Screening expansion by +20M, brief intervention expansion by +8M										
2030	M	2032 (1970, 2087)	-17.4 (-63.0, 25.7)	-0.8 (-3.0, 1.3)	1874 (1831, 1923)	-21.9 (-49.7, 8.6)	-1.2 (-2.6, 0.5)	1749 (1724, 1771)	-38.2 (-57.9, -18.7)	-2.1 (-3.2, -1.1)
2030	W	842 (814, 868)	-11.1 (-30.9, 9.0)	-1.3 (-3.6, 1.1)	651 (623, 681)	-10.7 (-26.1, 7.4)	-1.6 (-3.9, 1.1)	777 (759, 795)	-27.2 (-45.3, -8.6)	-3.4 (-5.6, -1.1)
Scenario 4: Universal screening and brief intervention										
2030	M	2026 (1964, 2084)	-23.0 (-53.5, 12.9)	-1.1 (-2.6, 0.6)	1866 (1821, 1917)	-30.4 (-59.7, -8.3)	-1.6 (-3.1, -0.4)	1732 (1700, 1753)	-55.6 (-83.9, -32.1)	-3.1 (-4.7, -1.8)
2030	W	837 (802, 868)	-16.3 (-38.0, 7.6)	-1.9 (-4.4, 0.9)	644 (619, 675)	-17.6 (-37.1, -3.0)	-2.7 (-5.4, -0.5)	759 (740, 780)	-45.5 (-68.3, -22.0)	-5.6 (-8.4, -2.8)

Note: CrI: Credible interval, 2.5th and 97.5th rank of the simulated expansion effect across 70 model iterations, M: Men, W: Women. Minimum brief intervention effect: M = -1.72, SE = 0.35.

© 2026 Lemp, JM et al. JAMA Health Forum.

eTable 15. Simulated combined YLL per 100,000 (sensitivity analysis 2).

		High school degree or less			Some college			College degree or more		
Year	Sex	YLL per 100k (CrI)	Absolute change in YLL (CrI)	Relative change in % (CrI)	YLL per 100k (CrI)	Absolute change in YLL (CrI)	Relative change in % (CrI)	YLL per 100k (CrI)	Absolute change in YLL (CrI)	Relative change in % (CrI)
Reference scenario (no expansion)										
2000	M	3516 (3511, 3520)	.	.	2180 (2175, 2185)	.	.	1096 (1093, 1099)	.	.
2000	W	912 (911, 914)	.	.	756 (753, 759)	.	.	456 (454, 458)	.	.
2030	M	2644 (2541, 2750)	.	.	1706 (1631, 1783)	.	.	830 (807, 859)	.	.
2030	W	1071 (1031, 1107)	.	.	856 (818, 889)	.	.	432 (415, 449)	.	.
Scenario 4: Universal screening and brief intervention										
2030	M	2547 (2449, 2647)	-96.8 (-147.1, -67.5)	-3.7 (-5.4, -2.5)	1650 (1580, 1737)	-56.6 (-90.2, -33.4)	-3.3 (-5.3, -2.0)	791 (760, 820)	-39.6 (-56.9, -17.4)	-4.8 (-6.7, -2.1)
2030	W	1000 (960, 1047)	-70.6 (-101.2, -41.1)	-6.6 (-9.5, -3.8)	803 (759, 844)	-52.8 (-87.4, -25.2)	-6.2 (-10.3, -2.9)	393 (368, 419)	-38.6 (-60.2, -21.5)	-8.9 (-13.9, -5.0)
		Non-Hispanic Black			Hispanic			Non-Hispanic White		
Year	Sex	YLL per 100k (CrI)	Absolute change in YLL (CrI)	Relative change in % (CrI)	YLL per 100k (CrI)	Absolute change in YLL (CrI)	Relative change in % (CrI)	YLL per 100k (CrI)	Absolute change in YLL (CrI)	Relative change in % (CrI)
Reference scenario (no expansion)										
2000	M	3144 (3135, 3152)	.	.	2877 (2862, 2887)	.	.	2532 (2530, 2534)	.	.
2000	W	857 (853, 861)	.	.	643 (640, 647)	.	.	777 (775, 778)	.	.
2030	M	2045 (1981, 2102)	.	.	1902 (1855, 1943)	.	.	1791 (1765, 1817)	.	.
2030	W	857 (820, 879)	.	.	664 (641, 689)	.	.	808 (796, 820)	.	.
Scenario 4: Universal screening and brief intervention										
2030	M	2010 (1947, 2071)	-34.7 (-77.7, -3.6)	-1.7 (-3.7, -0.2)	1851 (1805, 1897)	-50.2 (-88.2, -17.4)	-2.6 (-4.7, -0.9)	1708 (1667, 1740)	-83.6 (-114.1, -59.4)	-4.7 (-6.4, -3.3)
2030	W	832 (792, 868)	-24.2 (-49.7, -7.7)	-2.8 (-5.7, -0.9)	636 (608, 669)	-27.4 (-53.2, -7.8)	-4.1 (-7.9, -1.2)	737 (705, 766)	-71.5 (-104.7, -43.4)	-8.8 (-12.9, -5.4)

Note: CrI: Credible interval, 2.5th and 97.5th rank of the simulated expansion effect across 70 model iterations, M: Men, W: Women. Standard brief intervention effect: M = -2.86, SE = 0.58.

© 2026 Lemp, JM et al. JAMA Health Forum.

eTable 16. Cumulative YLL reductions per 100,000 across the 2025–2030 period (sensitivity analysis 3).

		Reference	Scenario 3: Screening expansion by +20M, brief intervention expansion by +8M							
			Roll-out in 2025		Roll-out in 2026		Roll-out in 2027		Roll-out in 2028	
Year	Sex	Cumulative YLL per 100k	Absolute cumulative change in YLL per 100k (CrI)	Relative cumulative change in % (CrI)	Absolute cumulative change in YLL per 100k (CrI)	Relative cumulative change in % (CrI)	Absolute cumulative change in YLL per 100k (CrI)	Relative cumulative change in % (CrI)	Absolute cumulative change in YLL per 100k (CrI)	Relative cumulative change in % (CrI)
2024	M	2063 (2048, 2076)	0.0 (0.0, 0.0)	.	.	.	.	.	.	.
2025	M	4082 (4052, 4111)	-8.5 (-19.8, 3.7)	-0.2 (-0.5, 0.1)	.	.	.	.	.	.
2026	M	6053 (6004, 6102)	-27.7 (-45.8, -13.9)	-0.5 (-0.8, -0.2)	-6.8 (-16.5, 1.3)	-0.1 (-0.3, 0.0)	.	.	.	.
2027	M	7980 (7909, 8042)	-47.0 (-79.8, -25.5)	-0.6 (-1.0, -0.3)	-19.1 (-35.6, -5.0)	-0.2 (-0.4, -0.1)	-4.7 (-13.5, 6.1)	-0.1 (-0.2, 0.1)	.	.
2028	M	9874 (9786, 9953)	-79.2 (-130.1, -51.4)	-0.8 (-1.3, -0.5)	-43.9 (-75.1, -24.6)	-0.4 (-0.8, -0.2)	-20.3 (-37.0, -4.7)	-0.2 (-0.4, 0.0)	-2.7 (-12.2, 6.4)	0.0 (-0.1, 0.1)
2029	M	11731 (11623, 11831)	-115.9 (-190.2, -78.1)	-1.0 (-1.6, -0.7)	-76.8 (-119.6, -46.0)	-0.7 (-1.0, -0.4)	-44.6 (-73.8, -20.0)	-0.4 (-0.6, -0.2)	-20.9 (-38.9, -6.9)	-0.2 (-0.3, -0.1)
2030	M	13557 (13428, 13675)	-169.0 (-261.3, -121.2)	-1.2 (-1.9, -0.9)	-121.9 (-181.6, -81.9)	-0.9 (-1.3, -0.6)	-76.9 (-117.0, -45.3)	-0.6 (-0.9, -0.3)	-52.2 (-77.1, -30.4)	-0.4 (-0.6, -0.2)
2024	W	789 (782, 796)	.	.	.	.	.	.	.	.
2025	W	1577 (1565, 1589)	-7.4 (-14.9, 1.0)	-0.5 (-0.9, 0.1)	.	.	.	.	.	.
2026	W	2357 (2340, 2376)	-16.7 (-28.4, -4.4)	-0.7 (-1.2, -0.2)	-2.9 (-7.8, 4.2)	-0.1 (-0.3, 0.2)	.	.	.	.
2027	W	3134 (3113, 3161)	-33.2 (-52.6, -14.0)	-1.1 (-1.7, -0.4)	-14.3 (-29.6, -3.6)	-0.5 (-0.9, -0.1)	-6.0 (-14.4, 2.1)	.	.	.
2028	W	3911 (3880, 3945)	-55.5 (-87.8, -26.8)	-1.4 (-2.2, -0.7)	-32.0 (-56.7, -15.3)	-0.8 (-1.4, -0.4)	-16.5 (-30.7, -3.2)	-0.4 (-0.8, -0.1)	-2.9 (-10.3, 4.5)	-0.1 (-0.3, 0.1)
2029	W	4685 (4649, 4726)	-81.4 (-126.9, -39.0)	-1.7 (-2.7, -0.8)	-55.4 (-89.6, -29.9)	-1.2 (-1.9, -0.6)	-31.4 (-51.3, -15.1)	-0.7 (-1.1, -0.3)	-14.0 (-26.2, -1.4)	-0.3 (-0.6, 0.0)
2030	W	5461 (5417, 5510)	-118.2 (-175.8, -64.0)	-2.2 (-3.2, -1.2)	-87.3 (-139.4, -49.7)	-1.6 (-2.5, -0.9)	-54.7 (-82.0, -28.8)	-1.0 (-1.5, -0.5)	-35.7 (-55.4, -16.4)	-0.7 (-1.0, -0.3)

Note: CrI: Credible interval, 2.5th and 97.5th rank of the simulated expansion effect across 70 model iterations, M: Men, W: Women. Standard brief intervention effect: M = -2.86, SE = 0.58.

© 2026 Lemp, JM et al. JAMA Health Forum.

eFigure 1. Simulated percentage of adults with hazardous alcohol use who received a brief intervention by sex and race and ethnicity.

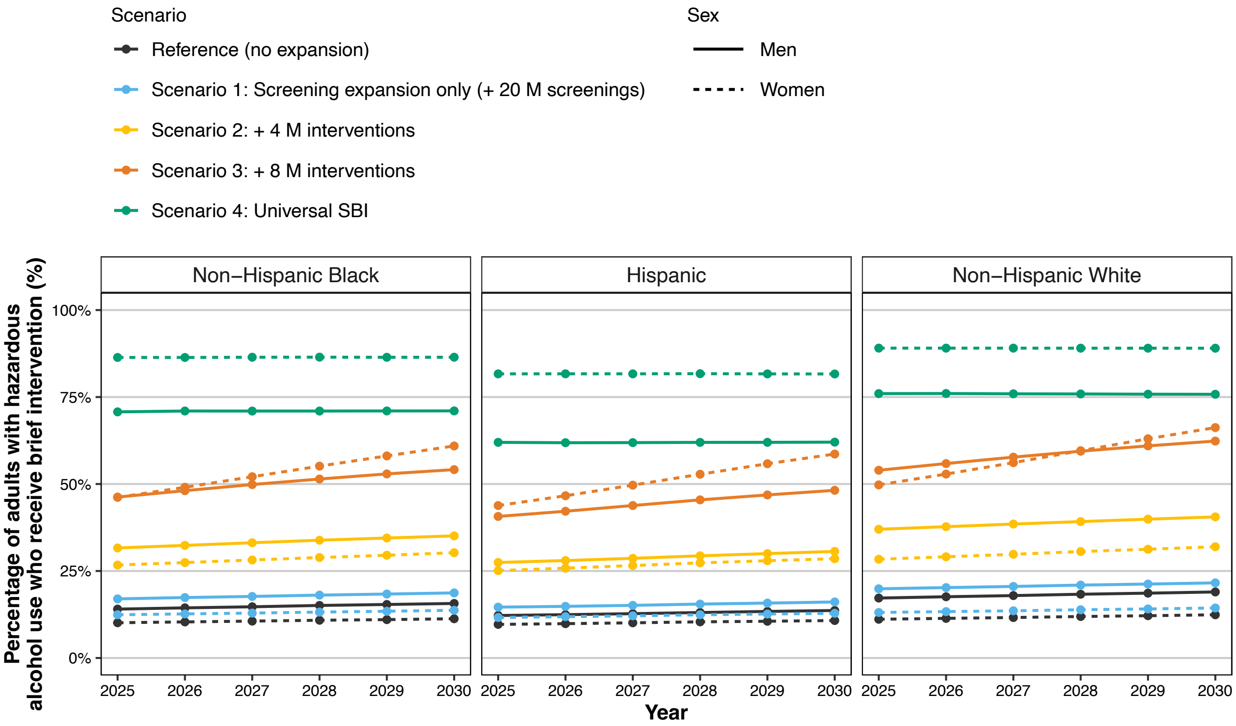

eFigure 1. Simulated mean percentage of adults with hazardous alcohol use (>20/40 GPD for women/men) who received a brief intervention each year, by sex and race and ethnicity, for each modelled scenario. Results are not reported for the 'Other' race and ethnicity category, as this group is both relatively small and highly heterogeneous, meaning model parameters could not be reliably informed by empirical data. Abbreviations: M = Million. SBI = Screening and brief intervention.

© 2026 Lemp, JM et al. JAMA Health Forum.

eFigure 2. Simulated mean prevalence of hazardous alcohol use by sex and race and ethnicity.

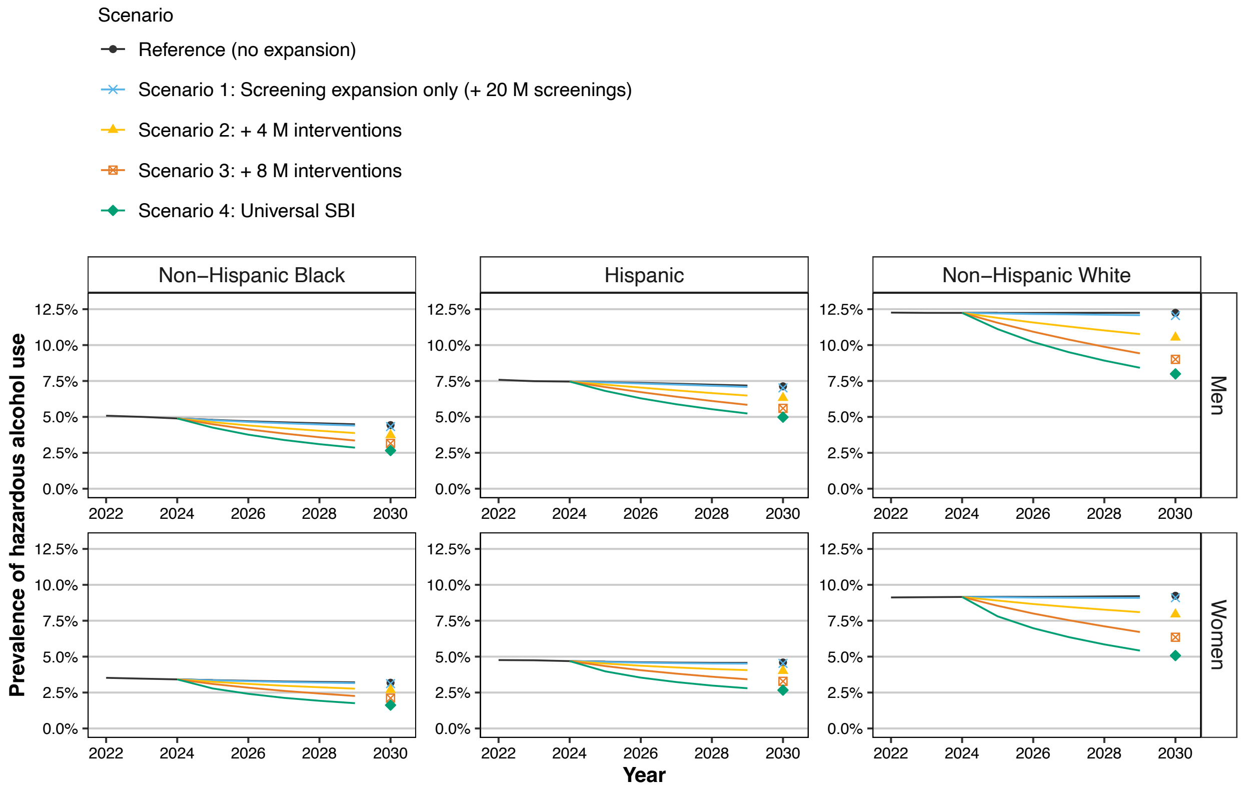

eFigure 2. Simulated mean prevalence of hazardous alcohol use (>20/40 GPD for women/men) by sex and race and ethnicity, for each modelled scenario. Results are not reported for the 'Other' race and ethnicity category as model parameters could not be reliably informed by empirical data. Abbreviations: M = Million. SBI = Screening and brief intervention.

© 2026 Lemp, JM et al. JAMA Health Forum.

eFigure 3. Simulated mean change in heavy episodic drinking prevalence by subgroup.

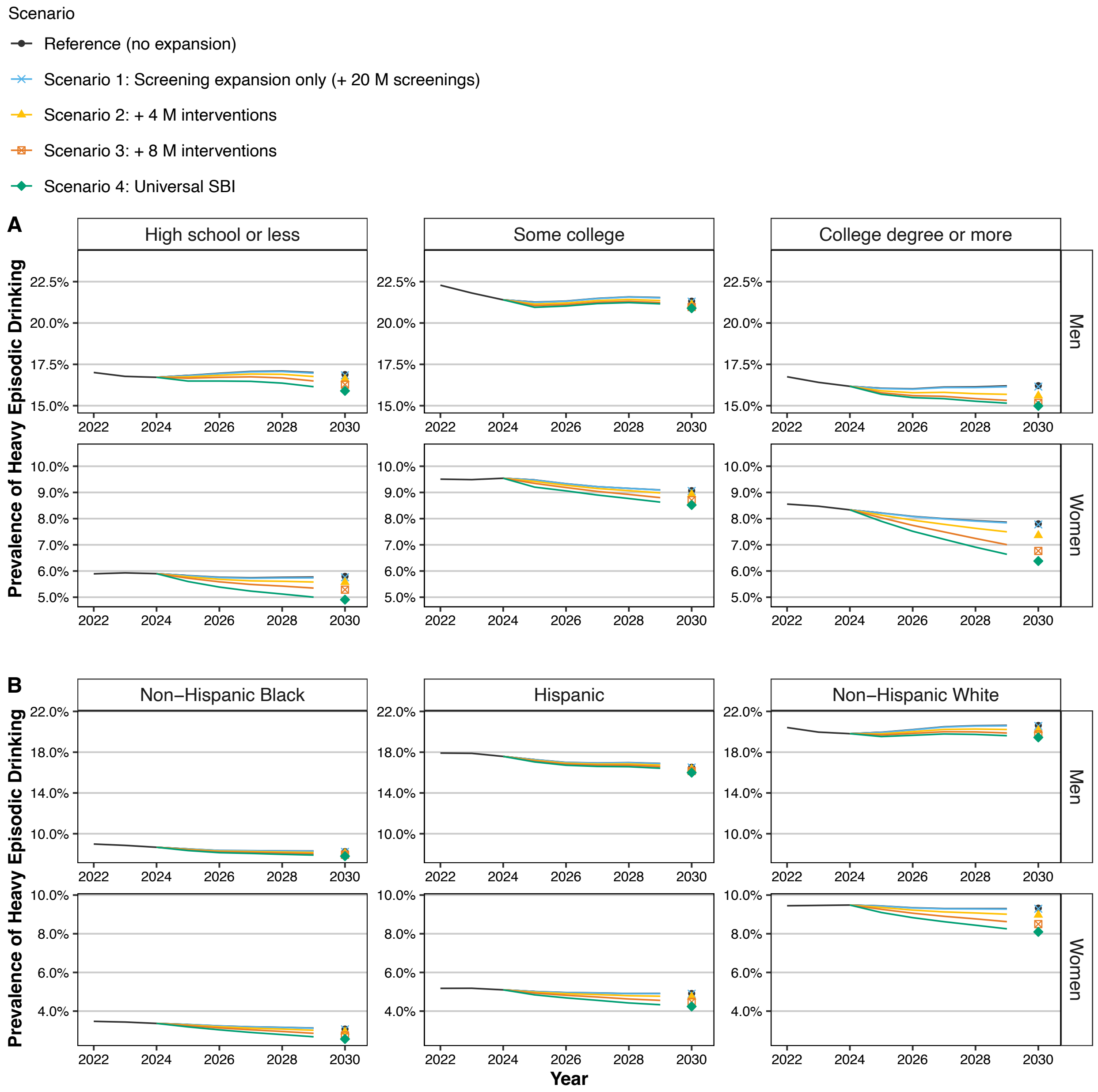

eFigure 3. Simulated mean prevalence of heavy episodic drinking (defined as ≥60 g of pure alcohol on at least one occasion in the past 30 days) by (A) sex and education and by (B) sex and race and ethnicity, for each modelled scenario. Results are not reported for the 'Other' race and ethnicity category as model parameters could not be reliably informed by empirical data. Abbreviations: M = Million. SBI = Screening and brief intervention.

© 2026 Lemp, JM et al. JAMA Health Forum.

eFigure 4. Simulated mean change in YLL per 100,000 by sex.

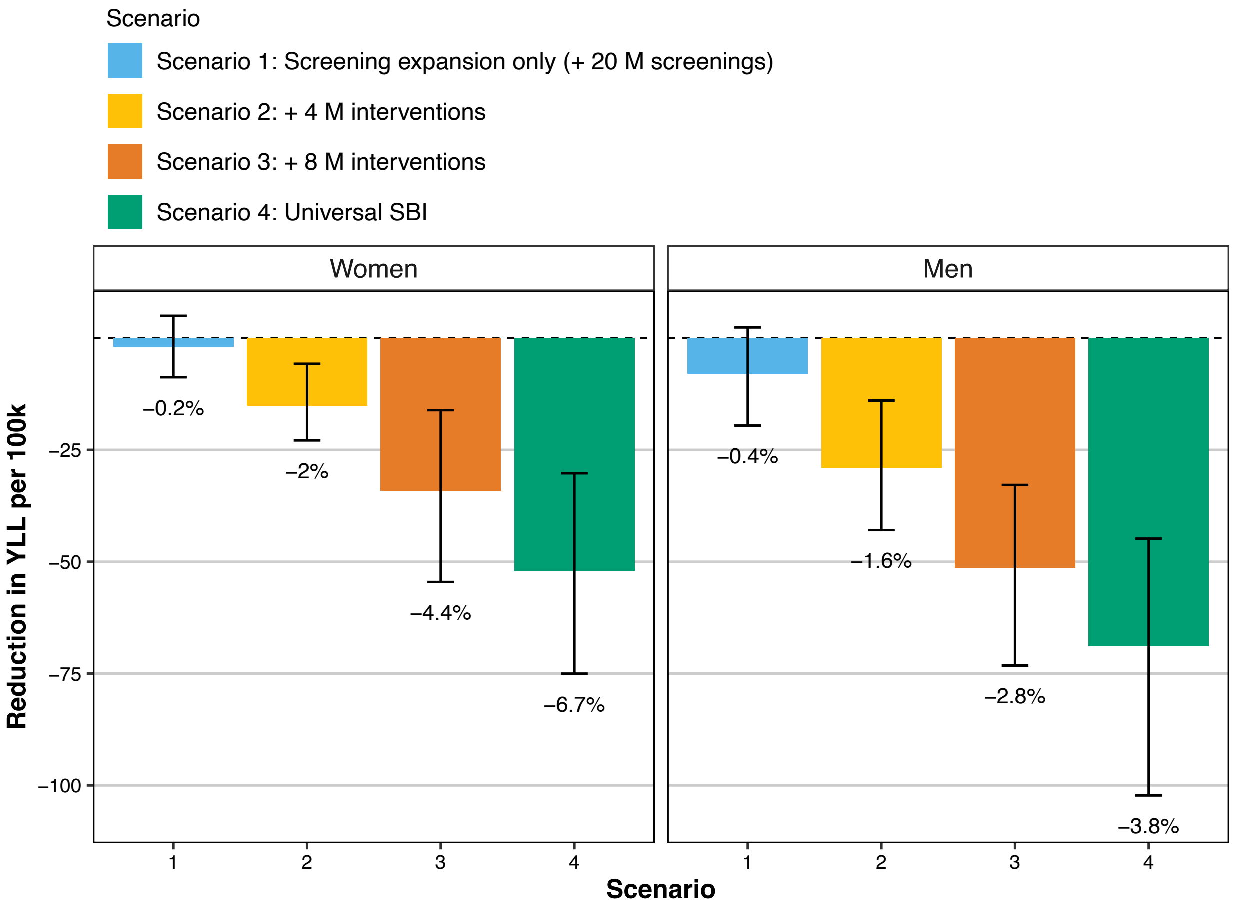

eFigure 4. Simulated mean change in YLL per 100,000 from five key alcohol-related causes for each modelled expansion scenario compared to the reference scenario (no expansion), in 2030, by sex. Abbreviations: M = Million. SBI = Screening and brief intervention. YLL per 100,000 = Years of Potential Life Lost per 100 000. Key alcohol-related causes of death are: alcohol use disorder (AUD, including alcohol poisonings), liver disease and cirrhosis (including hepatitis C-related liver cirrhosis), motor-vehicle injuries, other unintentional injuries, and suicide.

© 2026 Lemp, JM et al. JAMA Health Forum.

eFigure 5. Simulated mean change in combined YLL per 100,000 by sex, education and average brief intervention effect (sensitivity analysis 1).

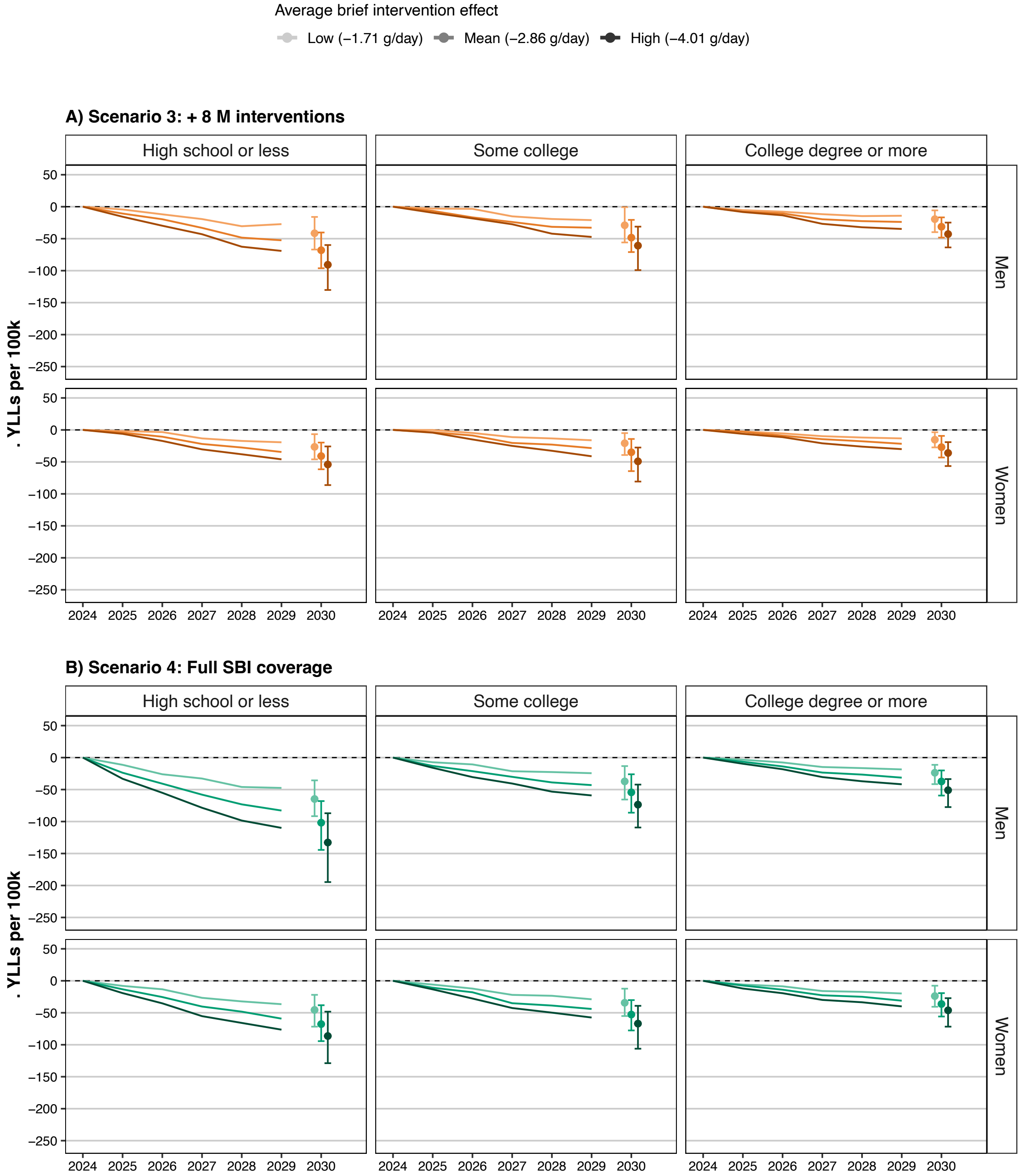

eFigure 5. Simulated mean change in YLL per 100,000 from key alcohol-related causes for expansion (A) scenario 3 and (B) scenario 4, compared to the reference scenario (no expansion), by sex, education and average brief intervention effect (sensitivity analysis 1). Abbreviations: M = Million. SBI = Screening and brief intervention. YLL per 100,000 = Years of Potential Life Lost per 100 000.

© 2026 Lemp, JM et al. JAMA Health Forum.

eFigure 6. Cumulative changes in YLL per 100,000 by sex across the 2025–2030 period (sensitivity analysis 3).

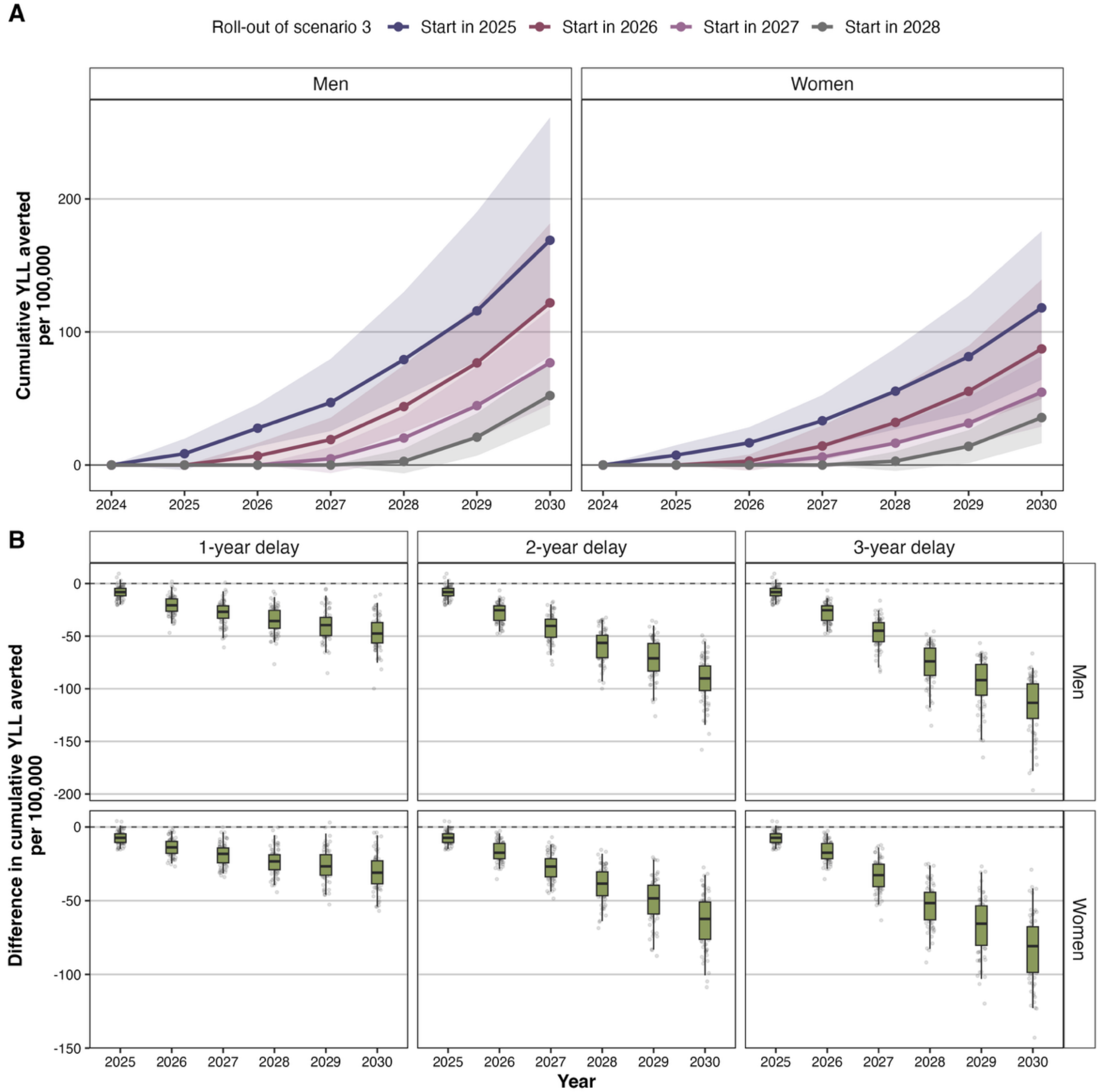

eFigure 6. Simulated cumulative changes in YLL per 100,000 from key alcohol-related causes for expansion scenario 3, (A) cumulative YLL per 100,000 averted compared to the reference scenario (no expansion) (B), cost of delaying the expansion by 1 year (start in 2026), 2 years (start in 2027), and 3 years (start in 2027) compared to roll-out in 2025, by sex (sensitivity analysis 3). Abbreviations: M = Million. SBI = Screening and brief intervention. YLL per 100,000 = Years of Potential Life Lost per 100 000. Key alcohol-related causes of death are: alcohol use disorder (AUD, including alcohol poisonings), liver disease and cirrhosis (including hepatitis C-related liver cirrhosis), motor-vehicle injuries, other unintentional injuries, and suicide.

© 2026 Lemp, JM et al. JAMA Health Forum.
