# Supplementary material for: Expanded Alcohol Screening and Brief Intervention to Address Premature Mortality
Source: JAMA Health Forum. 2026 Jul 31;7(7):e262348. doi: 10.1001/jamahealthforum.2026.2348 (PMC13428287; doi:10.1001/jamahealthforum.2026.2348)
Supplement: Supplement 2. — Data Sharing Statement [file jamahealthforum-e262348-s002.pdf]

Data Sharing Statement

Lemp. Expanded Alcohol Screening and Brief Intervention to Address Premature Mortality. *JAMA Health Forum*. Published July 31, 2026. doi:10.1001/jamahealthforum.2026.2348

Data
Data available: Yes
Data types: Other (please specify)
Additional Information: Several publicly available, secondary data sources were used, all of which are described and referenced in the overview, design concepts, and details (ODD) protocol in eMethods 1.
How to access data: Model parameters, including transition probabilities for modelling changes in education over time and beta distributions for grams per day within drinking categories, are shared via figshare (https://figshare.com/s/1355ee5cf3c8bd81b0dd). The model source code used in this publication will be made publicly available via GitHub (link to be included with final manuscript).
When available: With publication

Supporting Documents
Document types: Statistical/analytic code
How to access documents: The model source code used in this publication will be made publicly available via GitHub (https://doi.org/10.5281/zenodo.20862280). Mock data for running the source code will be included in the upcoming software release.
When available: With publication

Additional Information
Who can access the data: Anyone requesting the data
Types of analyses: Publicly available data can be used in line with respective regulations. Mock data will be made available upon publication of the SIMAH software for the purpose of performing simple model runs.
Mechanisms of data availability: Without investigator support.
